# Global identification and characterization of lncRNAs that control inflammation in malignant cholangiocytes

**DOI:** 10.1186/s12864-018-5133-8

**Published:** 2018-10-11

**Authors:** Bo-Wei Han, Hua Ye, Pan-Pan Wei, Bo He, Cai Han, Zhen-Hua Chen, Yue-Qin Chen, Wen-Tao Wang

**Affiliations:** 1 MOE Key Laboratory of Gene Function and Regulation, State Key Laboratory for Biocontrol, Sun Yat-sen University, Guangzhou, 510275 People’s Republic of China; 2 Department of Hepatobiliary, and Department of Anesthesiology, Sun Yat-sen Memorial Hospital, Sun Yat-sen University, Guangzhou, 510120 China

**Keywords:** CCA, lncRNA, Genome-wide, Diagnostic value, Inflammatory pathway

## Abstract

**Background:**

Long noncoding RNAs (lncRNAs) are known to play important roles in different cell contexts, including cancers. However, little is known about lncRNAs in cholangiocarcinoma (CCA), a cholangiocyte malignancy with poor prognosis, and associated with chronic inflammation and damage to the biliary epithelium. This study determined whether lncRNAs were dysregulated and participated in disease diagnosis or pivotal inflammation pathways through a genome-wide lncRNA screening and functional analysis.

**Results:**

We firstly identified a large number of lncRNAs abnormally expressed between 9 pairs of cancerous and adjacent tissues of CCA, and between intra-hepatic CCA and extra-hepatic CCA through a genome-wide profiling. A set of aberrant differentially expressed lncRNAs were further validated in a training set (16 pairs) and a test set (11 pairs) of CCA patient samples. Following assessment of the diagnostic value of the 7 differentially expressed lncRNAs, we confirmed the optimal combination of H19, C3P1, AC005550.3, PVT1, and LPAL2 with area under the curve of 0.8828 [95% CI: 0.7441–1.021, *P* < 0.001], with 93.75% sensitivity and 81.25% specificity, at the cutoff point of − 0.2884 to distinguish the CCA tissue from the normal ones, suggesting that specific lncRNAs may have potential for detecting CCA. More importantly, the genome-wide locus and lncRNA/mRNA co-expression analyses revealed a set of lncRNAs that participated in inflammation and oxidative stress response pathways by regulating genes *in cis* or *in trans*. Finally, APOC1P1, PVT1, and LPAL2 were validated to regulate the migration and some pivotal inflammation genes under the CCA pathogenesis.

**Conclusions:**

Our findings are the first to show that lncRNAs may not only be potential biomarkers of CCA progression but also respond to inflammation in CCA.

**Electronic supplementary material:**

The online version of this article (10.1186/s12864-018-5133-8) contains supplementary material, which is available to authorized users.

## Background

It has been shown that noncoding RNAs could serve as ideal diagnostic biomarkers and potential therapeutic targets for cancers [1–5]. Small non-coding RNAs, especially microRNAs (miRNAs), are widely reported to play vital roles in disease pathogenesis in a post-transcriptionally regulating manner [6], suggesting that these small noncoding RNAs could be served as therapeutic targets for the disease. In addition to miRNAs, a set of lncRNAs, that are more than 200 nucleotides in length, have also been reported to play pivotal roles in a variety of diseases. For instance, ANRIL [7], HOTAIR [8, 9], and H19 [10, 11], have been reported to influence many processes in various cancers, including chromatin remodeling, transcriptional regulation, molecular trafficking [12], inflammation responses and oxidative stress [1–3, 5]. These observations suggest potential pathophysiological contributions from lncRNAs. Recently, the mechanisms and functions of several lncRNAs, such as lncRNA-HEIH [13], HULC [14] and HOTAIR [15], were uncovered in hepatic carcinoma, the most common hepatic malignancy, and these findings led to the deeper understanding of the regulation networks and thetumorigenesis in hepatic carcinoma.

CCA is the second most common primary hepatic malignancy, and is broadly believed to be caused by the malignant transformation of cholangiocytes, the epithelial cells lining the intra-hepatic and extra-hepatic biliary ducts. CCA is difficult to diagnose and is usually fatal because of late clinical presentation and lack of effective targeting chemotherapeutic regimen [16, 17], which is partly due to the limited understanding of the molecular mechanisms underlying the development of CCA. Previous studies have reported that malignant transformation of cholangiocytes was associated with chronic inflammation in the biliary epithelium; however, detailed molecular mechanisms of CCA promotion and progression are still unclear.

In this study, we sought to determine whether lncRNAs were dysregulated and participated in molecular pathways associated with malignant transformation in CCA through genome-wide screening of lncRNA profiles in the CCA patients and evaluation of potential pathways that may involve dysregulated lncRNAs*.* We identified a set of lncRNAs that participated in inflammation and oxidative stress response pathways by regulating gene expression *in cis* or *in trans*. Our findings may provide potential biomarkers for CCA progression and potential CCA therapeutic targets.

## Methods

### Patient samples and cell line

Matched pairs of cancerous and adjacent tissue from 36 (9 for genome-wide profiling, 16 for training set, and 11 for test set independently) patients with CCA were obtained with informed consent from Sun Yat-sen Memorial Hospital. Sample collection was approved by the Hospital’s Protection of Human Subjects Committee. The clinicopathological characteristics of the CCA patients are summarized in Additional file 1: Table S1.

RBE human CCA cells were purchased from the Type Culture Collection of the Chinese Academy of Sciences (Shanghai, China). QBC939 human cholangiocarcinoma cells were obtained from Shuguang Wang (The Third Military Medical University, China). The CCA cell line was cultured in RPMI-1640 medium with 10% fetal calf serum, and maintained in a 37 °C humidified incubator with 5% CO_2_.

### LncRNA microarray profiling and qRT-PCR analysis

Tissues were porphyrized in liquid nitrogen, and cells were collected in EP tubes. Then, total RNA was extracted from tissue or cells using TRIzol reagent (Invitrogen, Carlsbad, CA, USA) following the manufacturer’s instructions.

Microarray analysis was performed with the Agilent Expression Array v2.0 platform (Capitalbio, Beijing, China), and gene expression data loaded into GEO database. The series number is GSE103909. The results represent differentially expressed lncRNAs and mRNAs with statistical significance that passed Volcano Plot filtering (Fold Change > 2.0, *P* < 0.05, t-test).

RNA was reverse-transcribed into cDNA with the ReverTra Ace® qPCR RT Kit (TOYOBO, Japan) or PrimeScript® RT reagent Kit with gDNA Eraser (Takara, Japan). Real-time PCR for lncRNA was performed using the SYBR Premix ExTaq real-time PCR kit (Takara, Japan) according to the manufacturer’s instructions with GAPDH as normalization control. The expression level for each lncRNA and mRNA was determined using the 2^-△△Ct^ method. The specificity and reliability of the PCR were confirmed by sequencing the PCR product fragments. All primers are shown in Additional file 2: Table S2.

### Cell transfection

We plated 1.6 × 10^5^ RBE and QBC939 cells in 12-well-plates. Then, 20 nM siRNAs (GenePharma, Shanghai, China) were transfected with Lipofectamine® 3000 (Invitrogen Corporation, Carlsbad, CA, USA). Each experiment was repeated at least three times. The siRNA sequences are: si-APOC1P1–1: 5’GGGAAUUCAUCAACCGCAUTT3’; si-APOC1P1–2: 5’AGUUUCUCCUUCACUUUCCGA3’; si-PVT1–1: 5’GCUUGGAGGCUGAGGAGUUTT3’, si-PVT1–2: 5’AGCUUUAGGUCACGUAAGGAC3’; and si-LPAL2:5’UCAAUCAGUGCUUGUUUGGAA3’, 5’AAGUAUGU GCCUCGAUAACUC3’.

#### Vector construction and transfetion

pcDNA 3.1 vector was used for lncRNA overexpression were using. Primers and oligonucleotides for full-length APOC1P1 amplification are list in Additional file 2: Table S2. Then, 500 ng full-length APOC1P1-pcDNA 3.1 vector were transfected with Lipofectamine® 3000 (Invitrogen Corporation, Carlsbad, CA, USA) into the 1.6 × 105 RBE cells and QBC939 in 12-well-plates.

### Migration assays

Migration assays were conducted using Transwell chambers (8 μm, Corning Costar Co., Newyork, USA) according to the manufacturer’s instructions. Firstly, 8 × 10^4^ RBE and QBC939 cells transfected after 24 h suspended in 200 μl RPMI-1640 solution without serum were plated in the top chambers lined with a non-coated membrane. Then, 750 μl RPMI-1640 with 10% fetal bovine serum was added into the lower chamber as a chemoattractant. Cells located in the lower chamber were fixed with 100% methanol and stained with 0.1% crystal violet after a 24 h incubation at 37 °C. We counted the migrated cells in ten fields for triplicate membranes at 10× magnification under a microscope (Zeiss,Oberkochen, Germany). Finally, five random sights in each sample were selected to analyze cell count, and the mean of triplicate experiments was calculated.

### Co-expression networks and gene ontology analysis

Pearson’s correlation coefficient was used to determine the levels of correlation. The correlation coefficients that were greater than 0.3 or less than − 0.3 were regared to be significant. LncRNAs and mRNAs that have significant pearson’s correlation coefficients were used to construct the coexpression networks using Cytoscape v2.83. DAVID Bioinformatics Resources v6.7 was used to identify enriched functionally related gene groups. For Gene Ontology (GO) analysis, we used the same biological processes and molecular function categories as the Gene Ontology Consortium database (http://www.geneontology.org).

### Statistical analysis in clinical samples

All statistical calculations were performed using SPSS PASW Statistics (version 17.0) and figures were imaged using GraphPad Prism (version 5.0). Fisher’s exact test and Mann-Whitney U test were used to determine the significance of differentially expressed lncRNA levels between two groups. Receiver operating characteristic (ROC) curves, analyzed after logarithmic transformation of all the data, were used to determine the diagnostic utility of lncRNAs. The optimized combination of lncRNAs to build a model of predicted probability analyzed by Discriminant analysis. The optimal cutoff point was chosen at which the Youden’s index was maximal. All *P* values were two tailed, and a *P* value < 0.05 was considered statistically significant.

The diagrammatic sketch of the study is shown in Additional file 3: Figure S1.

## Results

### LncRNA expression patterns are altered in malignant CCA

**Fig. 1:**
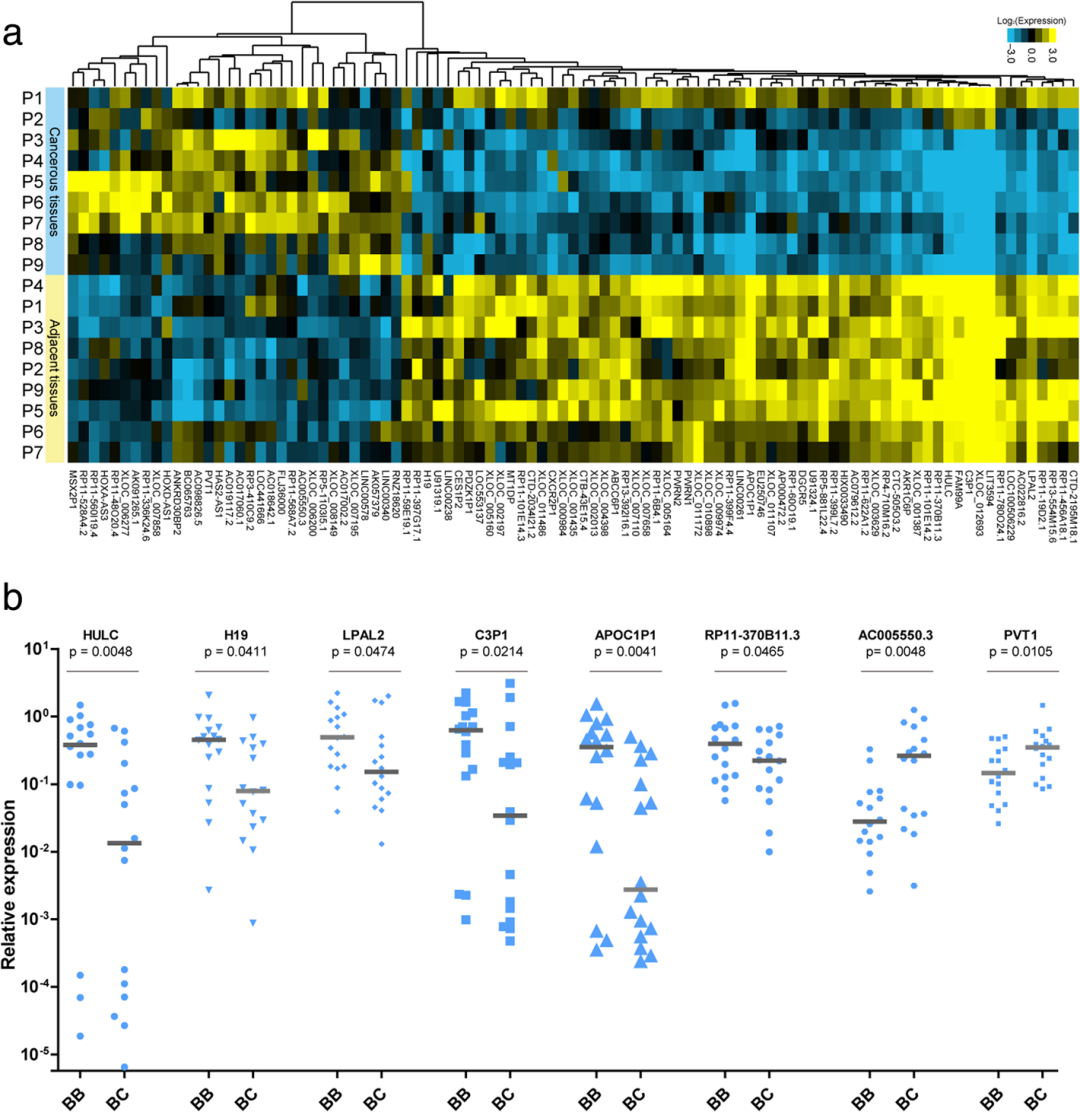
LncRNA profiles in CCA patients. **a** Cluster analysis of lncRNA expression in cancerous and adjacent tissues of CCA patients. **b** LncRNA expression validation using qPCR in training set. The relative expression levels of lncRNAs were normalized to GAPDH

**Fig. 2:**
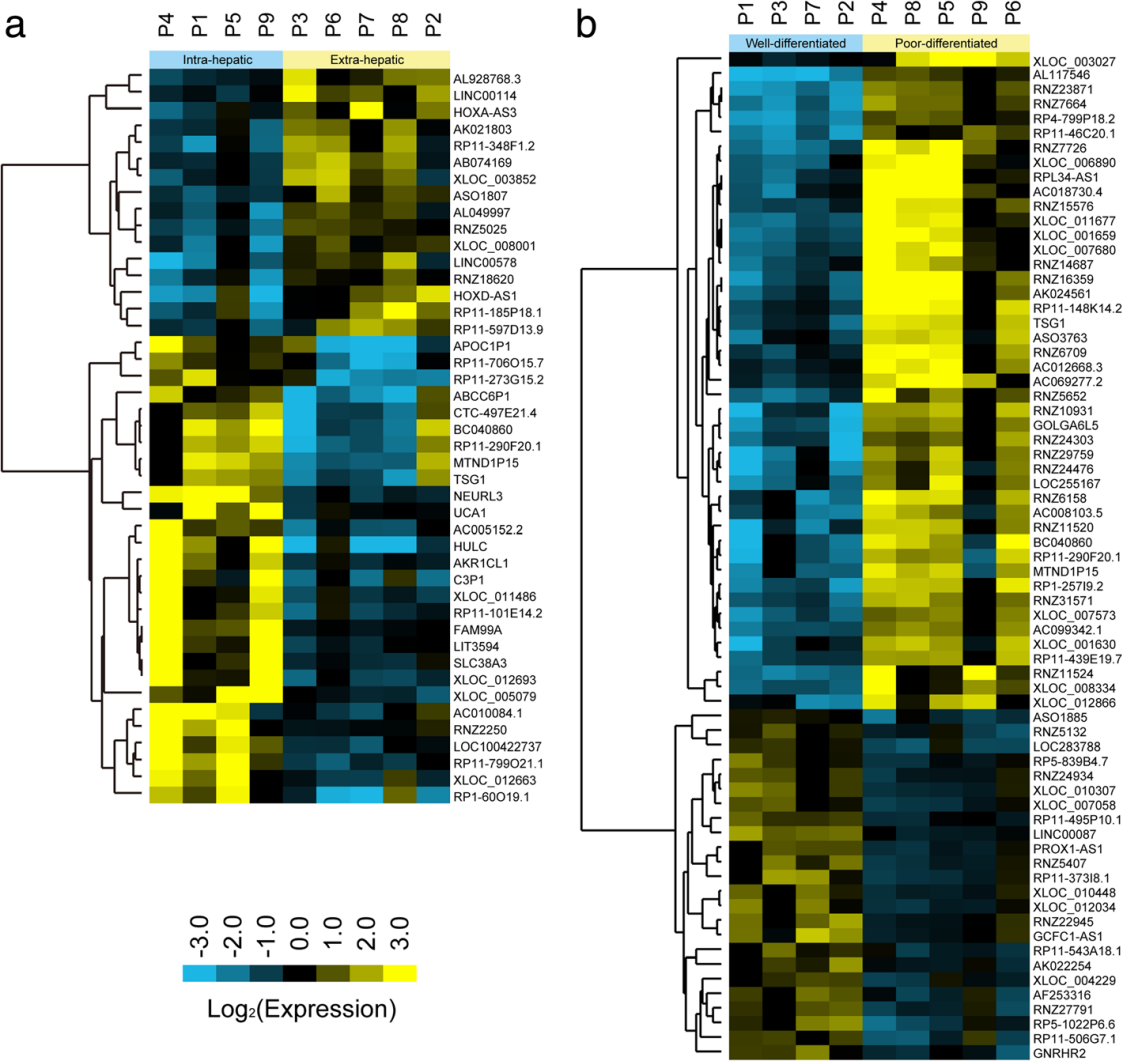
Differential expression of lncRNAs in subtypes of CCA. **a** Heat maps are based on differential lncRNA profiles between intra- and extra-hepatic cholangiocarcinoma patients. **b** Differentially expressed lncRNAs between well- and poorly-differentiated cholangiocarcinoma patients

To identify the aberrantly expressed lncRNAs that are linked to malignant transformation in CCA, a genome-wide lncRNA study was performed in the matched pairs of cancerous and adjacent tissues from patients with CCA. Unsupervised analysis showed that the samples were largely clustered into their respective biological subtypes, and all cancerous samples clustered together and were distinct from adjacent samples. We found that 583 lncRNAs were expressed abnormally in CCA tissue (*p*-value< 0.05 and fold change > 2). The Fig. 1a showed the most differentially expressed ones (*p* < 0.01, fold-change> 3.0) between cancerous and adjacent tissues, which include 32 significantly up-regulated lncRNAs and 65 significantly down-regulated lncRNAs in CCA tissues. Notably, a set of lncRNAs showed distinct expression patterns in intra-hepatic CCA and extra-hepatic CCA samples, which had different clinical characteristics and manifestations, suggesting that lncRNAs could potentially identify the location of cancerous lesions in CCA cells and discriminate different CCA subtypes (Fig. 2a). More importantly, in contrast to the patients with well-differentiated levels, there were 45 lncRNAs up-regulated and 24 lncRNAs down-regulated in the CCA patient sampleswith severe differentiation block (*p* < 0.05, fold-change> 3.0, Fig. 2b), and these lncRNAs might be associated with CCA cell differentiation.

To validate the accuracy of the lncRNA microarray platform, we performed qPCR assay for several lncRNAs in a training set of CCA patients (16 pairs of cancerous and adjacent tissues). First, we validated 3 well-known lncRNAs (H19, HULC, and PVT1), which have been reported to be involved in a variety of cancers [10, 14, 18–20]. Consistent with the microarray results, all of these lncRNAs showed significant differences between cancerous and adjacent tissues (*p* < 0.05, Fig. 1b). We also verified two up-regulated lncRNAs (RP11-528A4.2 and AC005550.3) and four down-regulated lncRNAs (C3P1, LPAL2, RP11–370B11.3, and APOC1P1) using qPCR(p < 0.05, Fig. 1b). The results confirmed the expression profiles of lncRNA array, except for RP11-528A4.2 (Additional file 4: Figure S2a) which did not show a significant difference. These data suggest that differentially expressed lncRNAs have the potential to serve as biomarkers for CCA and play important roles in the initiation or progression of CCA.

### Assessing the diagnostic value of lncRNAs in CCA

**Fig. 3:**
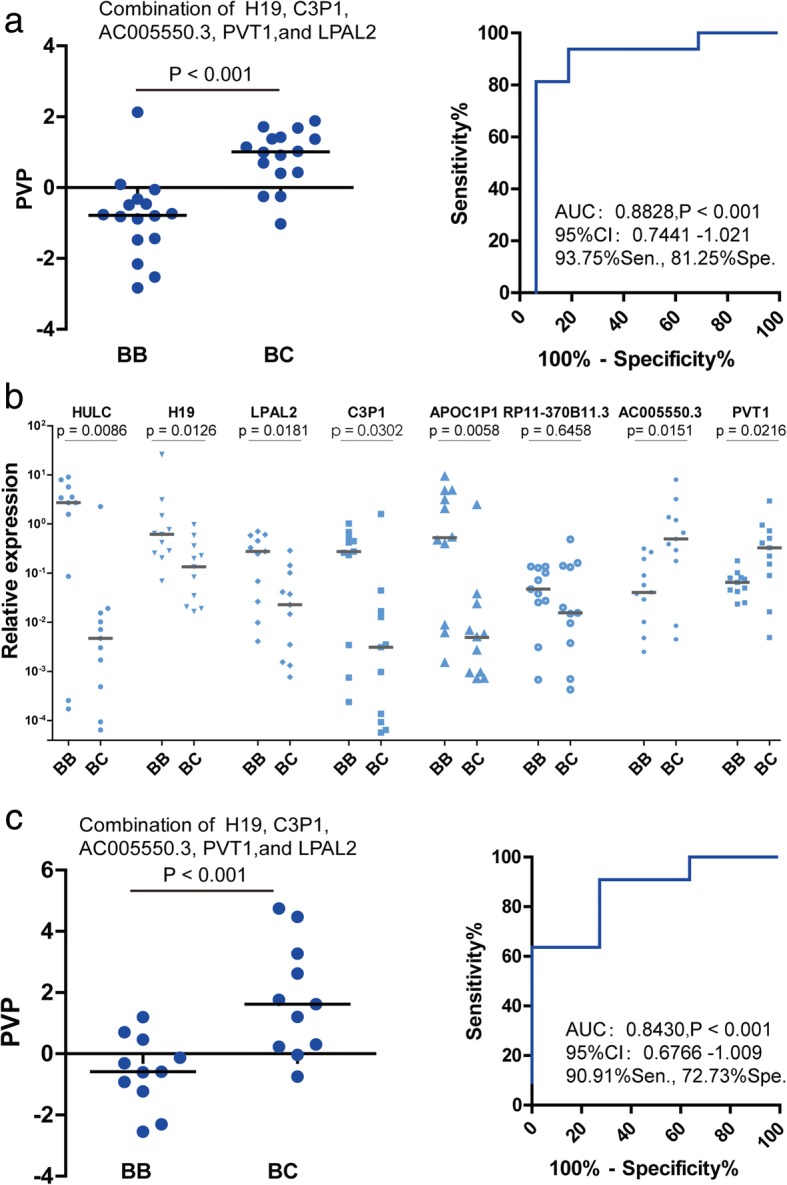
Diagnostic value of lncRNAs in CCA. **a** The optimal combination of H19, C3P1, AC005550.3, PVT1, and LPAL2 to distinguish the CCA tissue from the normal ones (*p* < 0.001,left); and the AUC of PVP was up to 0.8828 [95% CI: 0.7441–1.021, *P* < 0.001], with 93.75% sensitivity and 81.25% specificity, at the cutoff point of − 0.2884 (right). **b** Another 11 pairs of patient samples (11 cancerous and 11 adjacent tissues) were further validate the selected lncRNAs . **c** An AUC value of 0.8430 [95% CI: 0.6766–1.009, *P* < 0.001], as well as 90.91% sensitivity and 72.73% specificity was revealed when these test data was plugged into the PVP equation

To analyze the diagnostic value of these selected lncRNAs validated in the training set, ROC curve analysis was performed with the relative expression of these lncRNAs in logs, and the associated area under the ROC curve (AUC), as well as the sensitivity and specificity, was used to evaluate the diagnostic potency. As shown in Additional file 4: Figure S2, the highest AUC of the lncRNAs was AC005550.3, which reached 0.7695 [95% CI: 0.6015–0.9375, *P* < 0.01]. We also found that APOC1P1 had the greatest sensitivity, which was 87.5% at the cutoff point, whereas the highest was 93.75% for AC005550.3 at the cutoff point. Subsequently, using the discriminant analysis, we revealed the optimal combination of H19, C3P1, AC005550.3, PVT1, and LPAL2 to distinguish the CCA tissue from the normal ones. The following discriminant equation was determined: predicted value of probability (PVP) = 0.106 *ln*H19 + 0.237 *ln*C3P1 + 0.496 *ln*AC005550.3 + 0.699 *ln*PVT1–0.931 *ln*LPAL2 + 2.224. The result has shown more significant differences between cancerous and adjacent tissues (*p* < 0.001, Fig. 3a, left), and the enhanced AUC was up to 0.8828 [95% CI: 0.7441–1.021, *P* < 0.001], with 93.75% sensitivity and 81.25% specificity, at the cutoff point of − 0.2884 (Fig. 3a, right).

To explore the accuracy of the PVP equation, another 11 pairs of patient were recruited as a test set to further validate the outcomes. The results showed that the validities of the selected lncRNAs except for RP11–370B11.3 were confirmed in the validation set (Fig. 3b). Moreover, we provided these test data into the PVP equation, and got a AUC value of 0.8430 [95% CI: 0.6766–1.009, *P* < 0.001], as well as 90.91% sensitivity and 72.73% specificity, which is similar to that of training set (Fig. 3c). Although a large cohort of samples is necessary for further validation, these data suggested that these differential lncRNAs could serve as potential biomarkers of CCA.

### Dysregulated lncRNAs might be involved in inflammatory pathways by targeting adjacent genes *in cis*

To further investigate the potential functions of dysregulated lncRNAs, we performed a genome-wide evaluation for 583 dysregulated lncRNAs involved in potential pathways for the malignant transformation of CCA (*p* < 0.05, fold-change> 2.0). Many lncRNAs were reported to target their neighboring coding genes and shared similar functions with target genes [21], and these gene loci might play pivotal roles in the disease. Therefore, we screened the adjacent genes of the dysregulated lncRNAs with stringent selection criteria(intergenic distance< 10 kb, Additional file 5: Table S3), and then gene functions were analyzed using GO clustering(Additional file 6: Table S4.) [22]. It is worth noting that some GO annotations were related to inflammation and immunization (“activation of plasma proteins involved in acute inflammatory response,” “positive regulation of immune response,” “response to wounding”). Considering the close correlation between CCA and inflammation, the lncRNAs adjacent to inflammatory genes might participate in CCA through inflammatory pathways.

**Fig. 4:**
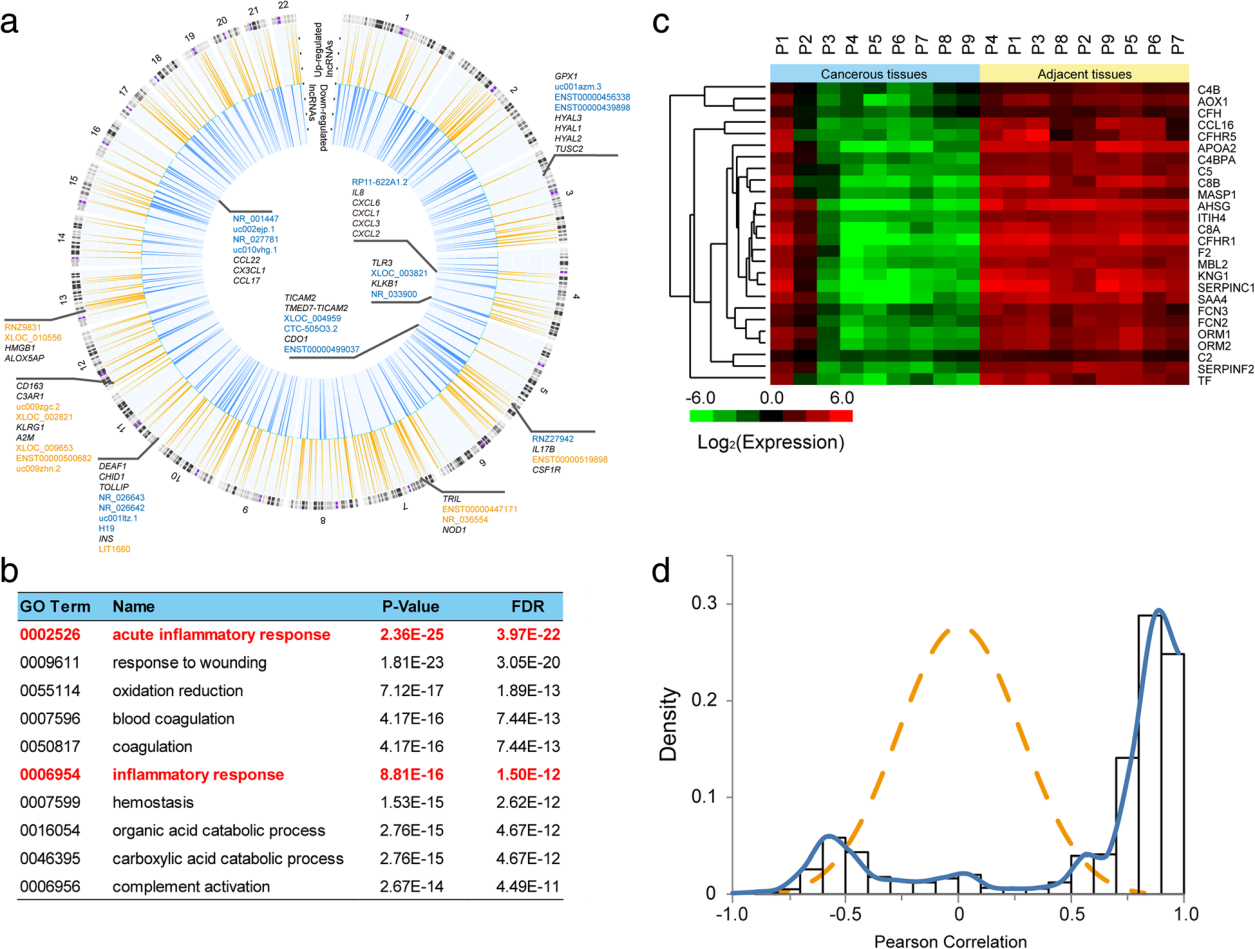
A set of lncRNAs potentially involved in inflammation-associated cytokine pathways. **a** Genome sites with a set of inflammation related genes and lncRNAs. Up-regulated lncRNAs are denoted in yellow, and down-regulated lncRNAs are denoted in blue. **b** GO clustering of dysregulated genes in CCA. **c** Abnormally expressed inflammation-related genes in CCA. **d** Pearson correlation of inflammation-related genes in cholangiocarcinoma, the dot dash in yellow is the normal distribution curve

To further investigate the correlation between inflammation and dysregulated lncRNAs in CCA, we extended the search coverage to ±100 kb for the dysregulation lncRNAs to improve the sensitivity of the screening. We found that 184 of the 583 lncRNAs were located nearby inflammation related genes (Additional file 7: Table S5), and several lncRNAs and inflammation related genes were found located in the same genome region (Fig. 4a). For example, three lncRNAs, XLOC_004959, CTC-505O3.2, and ENST00000499037, were adjacent to *TICAM2*, *TMED7-TICAM2*, and *CDO1*. Two of these genes (TICAM2 and TMED7-TICAM2) are members of the Toll-like receptor signaling pathway, which is important for host antiviral responses [23], lipopolysaccharide responses [24], and oxidative stress [25]. CDO1 is a cysteine dioxygenase involved in oxidation and inflammation [26], suggesting that the lncRNAs nearby might also be important for stress responses. *TLR3* is pivotal gene in the Toll-like receptor signaling pathway that controls the activation of inflammatory pathways [27], and another gene *KLKB1* [28] encodes a serine protease named kallikrein, which induces inflammatory reactions by producing pro-inflammatory peptides. We found that two lncRNAs, XLOC_003821 and NR_033900, were located near the respective gene locus of *TLR3* and *KLKB1*, suggesting that they might share similar functions. Further study of these genome regions within the set of inflammation related genes might provide more knowledge regarding the relationship between lncRNAs and inflammation in CCA.

### mRNA/lncRNA co-expression networks identified a set of lncRNAs involved in inflammation-associated cytokine pathways

Subsequently, we performed co-expression analyses of lncRNAs and mRNAs to screen potential target genes of lncRNA both *in cis* and *in trans*, which might reveal additional clues on the function of dysregulated lncRNAs [13, 29, 30]. Therefore, we performed mRNA microarrays using the same samples from the lncRNAs arrays, and the most dysregulated genes between CCA cancerous and adjacent tissues are shown in Additional file 8: Figure S3 (*P* < 0.01, fold-change> 3.0). Then, we used GO “biological process” to annotate gene functions and clustered the modules of genes (Fig. 4b). “Oxidation reduction” and other stress or inflammation-related GO terms were enriched, which is consistent with previous studies [31, 32]. Notably, the expression level of these inflammation-related genes was higher in adjacent tissues compared with cancerous tissues (Fig. 4c), which was similar to previous gene expression profile studies in CCA [33, 34].

In the genome-wide mRNA/lncRNA co-expression analysis, there are 299 lncRNAs that correlated with 522 mRNAs (Pearson correlation coefficient > 0.95), and most of these lncRNAs are positively correlated with mRNAs (Additional file 9: Table S6). We further focused on the lncRNAs that are closely related to these inflammatory genes from the GO term shown in Fig. 4c using co-expression networks. As expected, most inflammation-related genes showed positive correlations (Fig. 4d), implying that the lncRNAs involved in inflammatory responses might be co-expressed with these genes. Additional file 10: Figure S4 shows the dysregulated lncRNAs (red nodes) that were highly co-expressed (Pearson correlation *r* > 0.85) with inflammatory genes (green nodes), several well-studied lncRNAs and genes adjacent to inflammatory genes (e.g., CTC-505O3.2, an lncRNA located near *TICAM2*, *TMED7-TICAM2*, and *CDO1* in Fig. 4a) are co-expressed with a set of inflammatory genes. These candidate lncRNAs may take a part in known inflammatory pathways and participate in a variety of biological processes related to CCA.

### Functional validation of a set of lncRNAs involved in inflammation-associated cytokine pathways and carcinogenesis

**Fig. 5:**
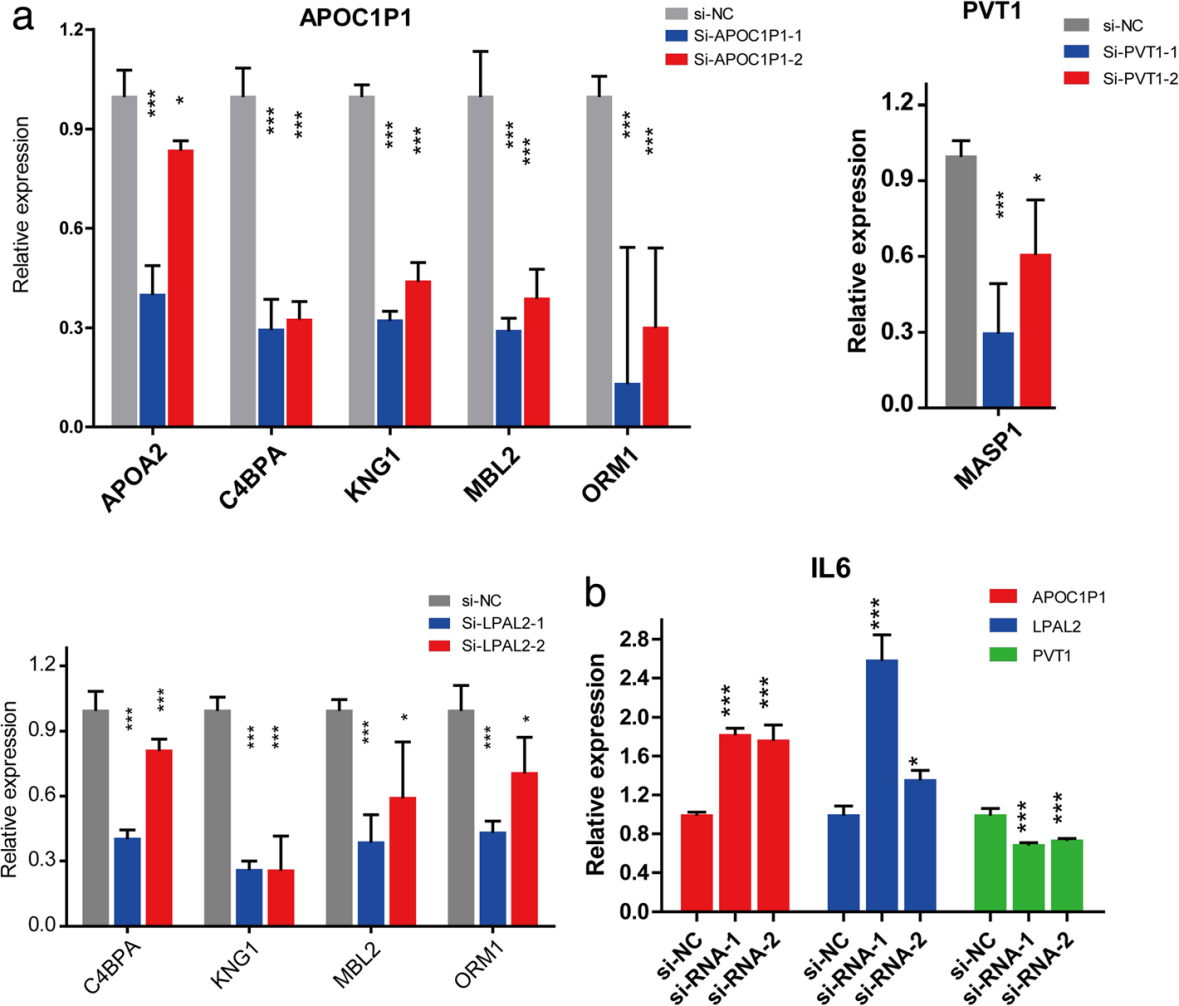
Functional validation of lncRNAs downreguated inflammation-associated genes in malignant cholangiocytes. **a** A set of the potentially targeted gens, like C4BPA, KNG1, MASP1, MBL2, etc., are significantly downregulated when the selected lncRNAs were knocked down by the siRNAs in RBE cells. **b** The qpcR test for the IL6 mRNA under the knockdown the selected lncRNAs targeted by siRNAs in CCA cell lines, and the triplicate experiments were analyzed by Mean ± SD, *p* value< 0.001, ***, < 0.01, **,< 0.05,*

**Fig. 6:**
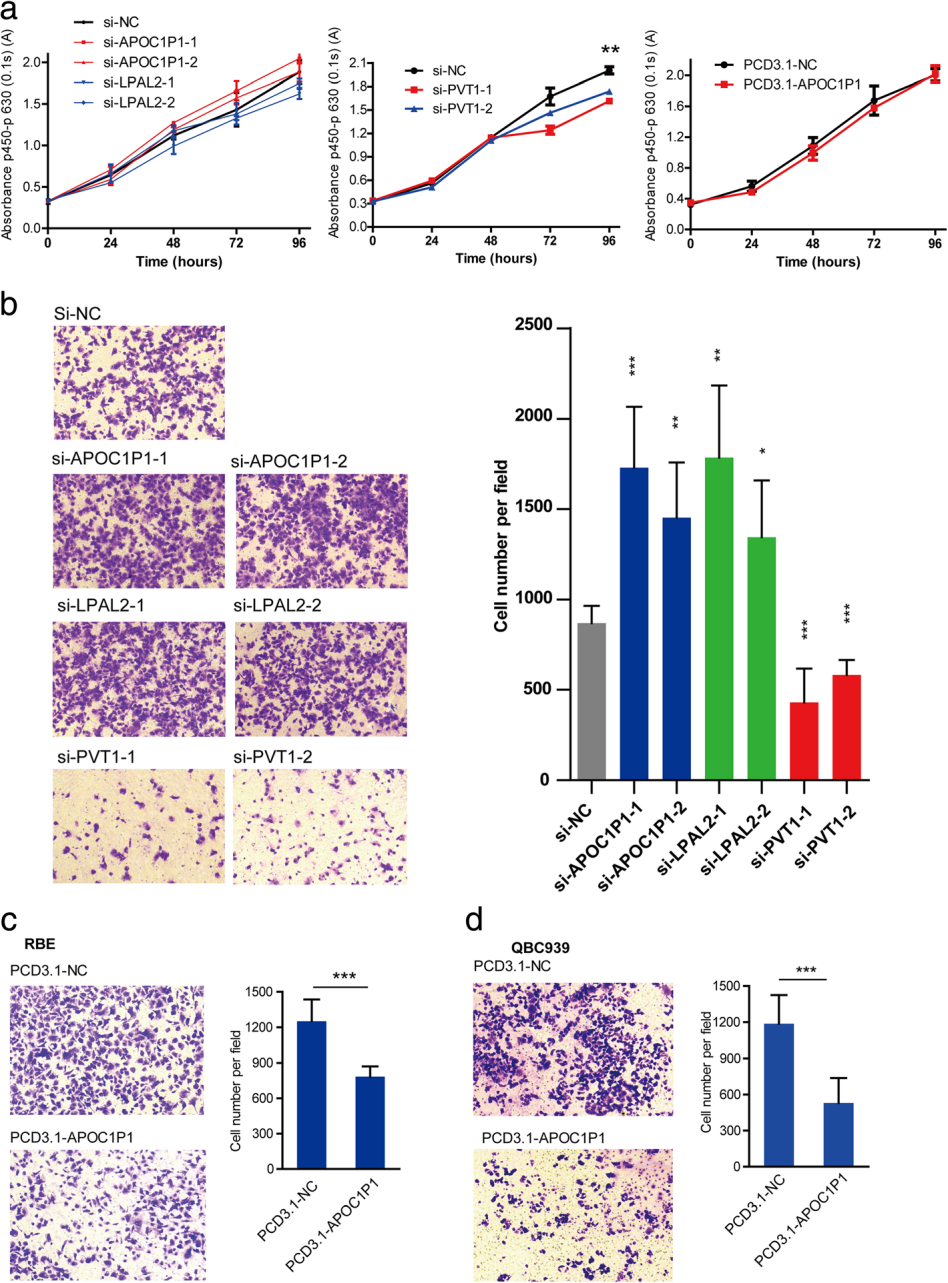
Functional validation of lncRNAs in malignant cholangiocytes. **a** Cell proliferation assay detected by CCK-8 kit in in malignant cholangiocytes. **b** Migration assay of these selected lncRNAs. RBE Cells were counted in ten fields for triplicate membranes at 10× magnification. Five random sights in each sample were selected to analyze cell count, and the triplicate experiments were analyzed by Mean ± SD, *p* value < 0.001, ***, < 0.01, **, < 0.05,*. Migration assay of overexpressing APOC1P1 in CCA cells (**c** for RBE, **d** for QBC939) were counted in ten fields for triplicate membranes at 10× magnification. Five random sights in each sample were selected to analyze cell count, and the triplicate experiments were analyzed by Mean ± SD, *p* value < 0.001, ***, < 0.01, **

To validate the regulation of the lncRNAs on the inflammatory genes shown in the network(Additional file 10: Figure S4), we next performed experiments to explore the their effect on the expression of these correlative inflammatory genes. Three differentially expressed lncRNAs were chosen, which are PVT1, LPAL2 and APOC1P1. Based on the PVP equation(Fig.3a and 3c), PVT1 showed a higher expression level in CCA tissue when compared with that in adjacent tissues, while the expression pattern of LPAL2 was quite opposite(Fig. 1b). APOC1P1 showed the lowest expression both in training set (Fig. 1b) and validation set (Fig. 3b) in CCA tissues and thus was also chosen for functional analysis. We knocked down APOC1P1, LPAL2, and PVT1 by RNA interference respectively. The expression levels were shown in Additional file 11: Figure S5. The results indicated that some of the potentially targeted genes, like C4BPA [35], MASP1 [36], MBL2 [37], ORM1 [38], APOA2 [39], KNG1 [40], are significantly downregulated upon knocking down the lncRNAs (Fig.5a and Additional file 12: Figure S6a),.On the contrary, the expression of these genes except for ORM1 appeared significantly increased when we overexpressed APOC1P1 in CCA cell line (Additional file 12: Figure S6b and c), suggesting that the inflammatory genes could be regulated by the selected lncRNAs in CCA cells. To confirm that the lncRNAs indeed regulate the inflammation pathway, we further detected the IL6 mRNA level, an important cytokine in inflammatory response and CCA progression [16, 17]. The IL6 levels displayed a considerable change upon knockdown of APOC1P1, LPAL2, and PVT1, indicating that these lncRNAs could affect the CAA progression by regulating IL6 level (Fig.5b and Additional file 12: Figure S6d). We next investigated the functions of these lncRNAs in CCA cells. As shown in Fig. 6a, the result of CCk-8 assay indicated that only PVT1 was shown to have an effect o the proliferation of CCA cellsa. We further conducted migration assays to demonstrate the function of these lncRNAs on CCA progression. We designed and synthetized two siRNAs to knock down these selected lncRNAs APOC1P1 and LPAL2, respectively. As shown in Fig. 6b, knocking down APOC1P1 and LPAL2 could significantly enhanced the ability of migration in RBE cells. This result was further validated when applying another CCA cell line QBC939 (Additional file 13: Figure S7). We also forced expression of APOC1P1 into two CCA cell lines, the results clearly showed that the migration abilities of RBE and QBC939 cells were decreased (Fig. 6c and 6d). These data imply that APOC1P1 and LPAL2 may be served as tumor suppressor genes in the CAA progression. On the other hand, when knocking down the PVT1, a lncRNA with a higher expression level in CCA (Fig. 1b& 3b**)**, it was found that the migration of both CCA cells significantly slowed down **(**Fig. 6b and Additional file 13: Figure S7), suggesting that PVT1 functions as a oncogene that promote the CAA progression. These lncRNAs dispayed different expression pattern in CCA, and may regulate different inflammation factors to affect the migration process, which led to CCA advancement .

## Discussion

The importance of dysregulated lncRNAs has been implicated in developmental regulation and disease pathogenesis, and the function of many dysregulated lncRNAs have been reported in various cancers [15, 29, 41]. However, few studies on lncRNAs associated with CCA have been reported. In this study, we screened dysregulated lncRNAs in CCA tissue and identified differential lncRNA patterns between cancerous and adjacent tissues. In addition, these lncRNAs could be involved in CCA promotion and serve as potential biomarkers for CCA. We further performed comparative analysis of the differential lncRNAs between CCA and other cancers and identified a set of lncRNAs specifically differentially expressed in CCA, and built a PVP equation to distinguish cancerous from adjacent tissues in CCA. We also found a set of lncRNAs potentially involved in inflammation pathways. Our study highlights the potential of lncRNAs in CCA clinical diagnosis and in understanding disease pathogenesis and development.

The malignant transformation of cholangiocytesis is associated with chronic inflammation in the biliary epithelium [16, 42]. Several lncRNAs, such as lncRNA-MIAT, lnc-IL7R and RP5-833A20.1, have been reported to regulate inflammation factors in other cancers [1, 43, 44]; however, lncRNAs that have been reported to regulate inflammation in CCA is limited. Thus, in this study, we performed a genome-wide screening and functional analysis to identify lncRNAs that control inflammation in malignant CCA. Our previously study reported that H19 and HULC, activated by oxidative stress, promote cell migration and invasion in cholangiocarcinoma through a ceRNA manner [45]. We also found treatment of CAA cells with IL6 can activate paracrine IL6/STAT3 pathway in inflammation and CCA initiation [46]. IL6 is a multifunctional inflammatory cytokine that plays a major role in the response of cholangiocytes to inflammation [47, 48], and increased concentrations of IL6 during inflammation in the biliary tract stimulate several pathways, including the JAK-STAT pathway, the p38 MAPK pathway, etc., and is involved in survival and growth of malignant cholangiocytes [48, 49]. CXCR4 is a chemokine receptor involved in several inflammatory processes and diseases, including CCA [50], and induces CCA cell migration and invasion via the ERK 1/2 and Akt pathways [51, 52]. However, a single lncRNA can regulate multiple target genes, for example, H19 targets TGFβ1 in prostate cancer cells [53], and TGFβ1 is a key regulator in CCA inflammation [16], suggesting the pivotal role of inflammation regulation by special lncRNAs. Our study on inflammation-related lncRNAs provides alternative lncRNA-based therapy targets for CCA.

Although some lncRNAs function through ceRNA pattern by sponging miRNAs [54, 55], other dysregulated lncRNAs in CCA might regulate coding genes by other methods. Many lncRNAs are shown to act *in cis* and control the expression level of neighboring genes through RNA-DNA, RNA-RNA or RNA-protein interactions [21]. To gain further insight into the biology of dysregulated lncRNAs in CCA, we screened the adjacent genes of these lncRNAs and tried to annotate their potential functions using GO biological process clusters. The most enriched annotations regard embryonic and organization development likely because of the high proportion of known lncRNAs located near the developmental genes [3, 5, 21, 56], and many developmental genes are also involve in carcinoma genesis [22]. For example, the lncRNAs HOXA-AS3 and HOXB-AS4 are adjacent to the *HOXA* and *HOXB* gene clusters, which encode essential proteins for developmental processes, and are also involved in numerous cancers, including CCA [57, 58]. Another example is PVT1, a lncRNA near *MYC*, which is a developmental gene involved in most cancers [59, 60]. There are also several lncRNAs located near transcription factors, including *FOXA2*, *FOXD4*, *RUNX1*, *WT1*, and *MEIS1*, which are involved in numerous cancers [59, 61–63], and the nearby lncRNAs might also control the level of these transcription factors, dysregulate gene patterns in bile duct cells, and induce tumorigenesis in CCA. The most attracted annotations were the inflammation related ones, and considering the importance of inflammatory responses in CCA pathogenesis, these lncRNAs, especially the lncRNAs located near the cluster of inflammatory factors listed in Fig. 5a, may potentially participate in inflammatory pathways that serve as pivotal regulators in CCA. However, aberrant epigenetic modifications of certain genomic sites are also major causes of carcinoma genesis, such as the aberrant DNA methylation level of chr13q14.3. in chronic lymphocytic leukemia [64] and the abnormal H3K79 histone methylation in chr7p15.2 in *MLL*-rearranged leukemia [29], and the observation that these lncRNAs act *in cis* in genome sites with inflammatory genes and dysregulate lncRNAs might provide new insight for suppressing or activating these genomic locations by imprinting modifications or transcriptional factor binding adjustments in CCA treatment.

Some lncRNAs might also regulate genes *in trans*, these lncRNAs transcriptionally and post-transcriptionally regulate their target genes. We also established co-expression networks in CCA samples to find potential functions for these lncRNAs. We found a set of lncRNAs, APOC1P1, LPAL2, and RP11–370B11.3, that is co-expressed with inflammatory factors without neighboring inflammatory genes, which implies that these lncRNAs might regulate inflammation pathways *in trans* or with ceRNA methods, although the detailed mechanisms remain unknown. For example, APOC1P1 could directly bind to tubulin to decrease α-tubulin acetylation, to inactivate caspase-3, and to inhibit apoptosis in breast cancer [65]. However, in this work, APOC1P1 was predicted and validated to correlate with a set of inflammatory genes, suggesting that APOC1P1 function the pivotal role of inflammation pathway under the CCA pathogenesis. Further studies on the mechanisms of the lncRNAs acting *in trans*, including identifying the protein partners they bind and screening the genes they directly target, might clarify the pathogenesis of inflammation induced by CCA and further provide strategies for developing lncRNA-based therapy methods for CCA.

Accumulating evidences have indicated that intra-hepatic and extra-hepatic CA have distinct differences in etiologies, origin of cell types and pathogenesis. Previously, Yang et al. [66] and Lv er al. [67] investigated the profiles of mRNAs and lncRNAs between intra-hepatic cholangiocarcinoma tissues and nomal controls, and found a set of dysreguated lncRNAs and mRNAs in intra-hepatic CCA samples. Some of the aberrantly expressed lncRNAs and mRNAs found in this study are similar with the reports in their analysis, such as NR_024470, uc010vdn.1, TMEM27, LAMC2, etc.. Moreover, some of the GO terms are also similar, like organic acid metabolic process and carboxylic acid catabolic process, indicating these lncRNAs may regulate the metabolic pathway in the CCA. However, some GO terms with high enrichmet are different. For example, Yang et al. revealed that lncRNAs with high enrichmet were associated with cholesterol homeostasis and sterol homeostasis, while inflammatory pathway was found the most enrichment in our study. The differences may indicate the complex biological mechanisms relevant to CCA progression and need further investigation. In addition to the comparison between cholangiocarcinoma tissues and nomal controls, we also performed a genome wide analysis between intra-hepatic and extra-hepatic CCA, and found several lncRNAs including HULC differentially expressed between intra-hepatic and extra-hepatic CCA. HULC has been reported to promote cell migration and invasion in cholangiocarcinoma through a ceRNA manner regulating CXCR4 [45]. CXCR4 was also reported important in the progression of intra-hepatic CCA [68]. These together suggest that HULC/CXCR4 might be a promising therapeutic target for intra-hepatic cholangiocarcinoma. Further studies are necessary to inviestigate the function of these differentially expressed lncRNAs in CCA formation and metastasis or the transformation between intra-hepatic and extra-hepatic cholangiocarcinoma.

## Conclusions

Our study revealed lncRNA profiles in CCA and the unique lncRNA expression patterns between intra- and extra-hepatic CCA patient samples with distinct differentiation levels. Based on the expression pattern analysis of the adjacent genes and co-expressed genes of lncRNAs, we identified a set of dysregulated lncRNAs correlated with inflammation, which may play key roles in the pathogenesis of CCA. We finally confirmed the optimal combination of H19, C3P1, AC005550.3, PVT1, and LPAL2 to differentiate the CCA tissue from the normal ones, suggesting that specific lncRNAs may have potential for detecting CCA. Our results provide new insight into the mechanism linking lncRNA function with CCA and may serve as novel targets for the development of new countermeasures of CCA.

## Additional files

Additional file 1: **Table S1.** Patient demographics and clinicopathologic features. (DOCX 22 kb)

Additional file 2: **Table S2.** Primer sequence for qPCR and vector construction. (DOCX 23 kb)

Additional file 3: **Figure S1.** The framework of the study. (TIF 450 kb)

Additional file 4: **Figure S2.** Assessment of the diagnostic accuracy of these special lncRNAs for Cholangiocarcinoma. (a) the expression of RP11-528A4.2. Diagnostic value of lncRNAs for Cholangiocarcinoma: HULC(b), H19(c), LPAL2(d), C3P1(e), AC005550.3(f), APOC1P1(g), PVT1(h),and sensitivity: Sen. for short, specificity: Spe. for short. (TIF 638 kb)

Additional file 5: **Table S3.** Adjacent genes of dysregulated lncRNAs. (DOCX 80 kb)

Additional file 6: **Table S4.** GO clusters of adjacent genes of dysregulated lncRNAs. (DOCX 33 kb)

Additional file 7: **Table S5.** Dysregulated lncRNAs adjacent to inflammatory genes. (DOCX 50 kb)

Additional file 8: **Figure S3.** Cluster analysis of mRNA expression in cancerous and adjacent tissues of cholangiocarcinoma patients (TIF 1712 kb)

Additional file 9: **Table S6.** Genome-wide correlation between lncRNA and mRNA levels (Pearson correlation coefficient > 0.95). (DOCX 221 kb)

Additional file 10: **Figure S4.** The network of dysregulated lncRNA and the co-expressed inflammatory genes. The dysregulated lncRNAs (red nodes) that were highly co-expressed (Pearson correlation *r* > 0.85) with inflammatory genes (green nodes). (TIF 740 kb)

Additional file 11: **Figure S5.** The qpcR test for knockdown efficiency of the selected lncRNAs targeted by siRNAs in CCA cell lines. (a) RBE and (b)QBC939 cells. (TIF 537 kb)

Additional file 12: **Figure S6.** Functional validation of selected lncRNAs in malignant cholangiocytes. (a) A set of the potentially targeted gens, like C4BPA, KNG1, MASP1, MBL2, etc., are significantly downregulated when APOC1P1, PVT1, and LPAL2 were knocked down by the siRNAs in CCA cells. (b) The qpcR test for the IL6 mRNA under the knockdown the selected lncRNAs targeted by siRNAs in QBC939. (c) The qpcR test for overexpression efficiency of APOC1P1 in CCA cell lines. (d) A set of the potentially targeted gens, like C4BPA, KNG1, MASP1, MBL2, etc., are significantly upregulated when APOC1P1 were overexpressed in QBC939. (TIF 929 kb)

Additional file 13: **Figure S7.** Migration of QBC939 knocking down of selected lncRNAs in malignant cholangiocytes. QBC939 Cells were counted in ten fields for triplicate membranes at 10× magnification. Five random sights in each sample were selected to analyze cell count, and the triplicate experiments were analyzed by Mean ± SD, *p* value< 0.001, ***, < 0.01, **. (TIF 3990 kb)

## Abbreviations

AUC: Associated area under the ROC curve
CCA: Cholangiocarcinoma
CI: Confidence interval
GO: Gene Ontology
lncRNAs: Long noncoding RNAs
MiRNAs: MicroRNAs
PVP: Predicted value of probability
ROC: Receiver operating characteristic

## References

[1] Cui H, Xie N, Tan Z, Banerjee S, Thannickal VJ, Abraham E (2014). The human long noncoding RNA lnc-IL7R regulates the inflammatory response. Eur J Immunol.

[2] Krawczyk M, Emerson BM (2014). p50-associated COX-2 extragenic RNA (PACER) activates COX-2 gene expression by occluding repressive NF-kappaB complexes. elife.

[3] Liu Y, Luo F, Xu Y, Wang B, Zhao Y, Xu W (2015). Epithelial-mesenchymal transition and cancer stem cells, mediated by a long non-coding RNA, HOTAIR, are involved in cell malignant transformation induced by cigarette smoke extract. Toxicol Appl Pharmacol.

[4] Manca S, Magrelli A, Cialfi S, Lefort K, Ambra R, Alimandi M (2011). Oxidative stress activation of miR-125b is part of the molecular switch for Hailey-Hailey disease manifestation. Exp Dermatol.

[5] Yarmishyn AA, Batagov AO, Tan JZ, Sundaram GM, Sampath P, Kuznetsov VA (2014). HOXD-AS1 is a novel lncRNA encoded in HOXD cluster and a marker of neuroblastoma progression revealed via integrative analysis of noncoding transcriptome. BMC Genomics.

[6] Chen L, Yan HX, Yang W, Hu L, Yu LX, Liu Q (2009). The role of microRNA expression pattern in human intrahepatic cholangiocarcinoma. J Hepatol.

[7] Huang MD, Chen WM, Qi FZ, Xia R, Sun M, Xu TP (2015). Long non-coding RNA ANRIL is upregulated in hepatocellular carcinoma and regulates cell apoptosis by epigenetic silencing of KLF2. J Hematol Oncol.

[8] Loewen G, Jayawickramarajah J, Zhuo Y, Shan B (2014). Functions of lncRNA HOTAIR in lung cancer. J Hematol Oncol.

[9] Teschendorff AE, Lee SH, Jones A, Fiegl H, Kalwa M, Wagner W (2015). HOTAIR and its surrogate DNA methylation signature indicate carboplatin resistance in ovarian cancer. Genome Med.

[10] Iizuka N, Oka M, Tamesa T, Hamamoto Y, Yamada-Okabe H (2004). Imbalance in expression levels of insulin-like growth factor 2 and H19 transcripts linked to progression of hepatocellular carcinoma. Anticancer Res.

[11] Charlton J, Williams RD, Sebire NJ, Popov S, Vujanic G, Chagtai T (2015). Comparative methylome analysis identifies new tumour subtypes and biomarkers for transformation of nephrogenic rests into Wilms tumour. Genome Med..

[12] Han BW, Chen YQ (2013). Potential pathological and functional links between long noncoding RNAs and hematopoiesis. Sci Signal.

[13] Yang F, Zhang L, Huo XS, Yuan JH, Xu D, Yuan SX (2011). Long noncoding RNA high expression in hepatocellular carcinoma facilitates tumor growth through enhancer of zeste homolog 2 in humans. Hepatology.

[14] Wang J, Liu X, Wu H, Ni P, Gu Z, Qiao Y (2010). CREB up-regulates long non-coding RNA, HULC expression through interaction with microRNA-372 in liver cancer. Nucleic Acids Res.

[15] Li G, Zhang H, Wan X, Yang X, Zhu C, Wang A (2014). Long noncoding RNA plays a key role in metastasis and prognosis of hepatocellular carcinoma. Biomed Res Int.

[16] Fava G, Lorenzini I (2012). Molecular pathogenesis of cholangiocarcinoma. Int J Hepatol.

[17] Rizvi S, Gores GJ (2013). Pathogenesis, diagnosis, and management of cholangiocarcinoma. Gastroenterology.

[18] Zeng C, Yu X, Lai J, Yang L, Chen S, Li Y (2015). Overexpression of the long non-coding RNA PVT1 is correlated with leukemic cell proliferation in acute promyelocytic leukemia. J Hematol Oncol.

[19] Ding C, Yang Z, Lv Z, DU C, Xiao H, Peng C (2015). Long non-coding RNA PVT1 is associated with tumor progression and predicts recurrence in hepatocellular carcinoma patients. Oncol Lett.

[20] Zhao Y, Guo Q, Chen J, Hu J, Wang S, Sun Y (2014). Role of long non-coding RNA HULC in cell proliferation, apoptosis and tumor metastasis of gastric cancer: a clinical and in vitro investigation. Oncol Rep.

[21] Villegas VE, Zaphiropoulos PG (2015). Neighboring gene regulation by antisense long non-coding RNAs. Int J Mol Sci.

[22] Blake JACKDM (2015). Gene ontology consortium: going forward. Nucleic Acids Res.

[23] Lang T, Lo C, Skinner N, Locarnini S, Visvanathan K, Mansell A (2011). The hepatitis B e antigen (HBeAg) targets and suppresses activation of the toll-like receptor signaling pathway. J Hepatol.

[24] Sacre SM, Lundberg AM, Andreakos E, Taylor C, Feldmann M, Foxwell BM (2007). Selective use of TRAM in lipopolysaccharide (LPS) and lipoteichoic acid (LTA) induced NF-kappaB activation and cytokine production in primary human cells: TRAM is an adaptor for LPS and LTA signaling. J Immunol.

[25] Kenny EF, O'Neill LA (2008). Signalling adaptors used by toll-like receptors: an update. Cytokine.

[26] Wilkinson LJ, Waring RH (2002). Cysteine dioxygenase: modulation of expression in human cell lines by cytokines and control of sulphate production. Toxicol in Vitro.

[27] Miyake K (2007). Innate immune sensing of pathogens and danger signals by cell surface toll-like receptors. Semin Immunol.

[28] Hayama Tomomi, Kamio Naoto, Okabe Tatsu, Muromachi Koichiro, Matsushima Kiyoshi (2016). Kallikrein Promotes Inflammation in Human Dental Pulp Cells Via Protease-Activated Receptor-1. Journal of Cellular Biochemistry.

[29] Fang K, Han BW, Chen ZH, Lin KY, Zeng CW, Li XJ (2014). A distinct set of long non-coding RNAs in childhood MLL-rearranged acute lymphoblastic leukemia: biology and epigenetic target. Hum Mol Genet.

[30] Wang KC, Chang HY (2011). Molecular mechanisms of long noncoding RNAs. Mol Cell.

[31] Kawanishi S, Hiraku Y, Pinlaor S, Ma N (2006). Oxidative and nitrative DNA damage in animals and patients with inflammatory diseases in relation to inflammation-related carcinogenesis. Biol Chem.

[32] Reuter S, Gupta SC, Chaturvedi MM, Aggarwal BB (2010). Oxidative stress, inflammation, and cancer: how are they linked?. Free Radic Biol Med.

[33] Andersen JB, Spee B, Blechacz BR, Avital I, Komuta M, Barbour A (2012). Genomic and genetic characterization of cholangiocarcinoma identifies therapeutic targets for tyrosine kinase inhibitors. Gastroenterology.

[34] Chapman MH, Tidswell R, Dooley JS, Sandanayake NS, Cerec V, Deheragoda M (2012). Whole genome RNA expression profiling of endoscopic biliary brushings provides data suitable for biomarker discovery in cholangiocarcinoma. J Hepatol.

[35] Olivar R, Luque A, Naranjo-Gomez M, Quer J, DFP G, Borras FE (2013). The alpha7beta0 isoform of the complement regulator C4b-binding protein induces a semimature, anti-inflammatory state in dendritic cells. J Immunol.

[36] Takahashi M, Ishida Y, Iwaki D, Kanno K, Suzuki T, Endo Y (2010). Essential role of mannose-binding lectin-associated serine protease-1 in activation of the complement factor D. J Exp Med.

[37] Kang JH, Super M, Yung CW, Cooper RM, Domansky K, Graveline AR (2014). An extracorporeal blood-cleansing device for sepsis therapy. Nat Med.

[38] Cai L, Oyeniran C, Biswas DD, Allegood J, Milstien S, Kordula T (2016). ORMDL proteins regulate ceramide levels during sterile inflammation. J Lipid Res.

[39] Sigel S, Bunk S, Meergans T, Doninger B, Stich K, Stulnig T (2012). Apolipoprotein B100 is a suppressor of Staphylococcus aureus-induced innate immune responses in humans and mice. Eur J Immunol.

[40] Kata D, Foldesi I, Feher LZ, Hackler LJ, Puskas LG, Gulya K (2017). A novel pleiotropic effect of aspirin: beneficial regulation of pro- and anti-inflammatory mechanisms in microglial cells. Brain Res Bull.

[41] Xu TP, Huang MD, Xia R, Liu XX, Sun M, Yin L (2014). Decreased expression of the long non-coding RNA FENDRR is associated with poor prognosis in gastric cancer and FENDRR regulates gastric cancer cell metastasis by affecting fibronectin1 expression. J Hematol Oncol.

[42] Landskron G, De la Fuente M, Thuwajit P, Thuwajit C, Hermoso MA (2014). Chronic inflammation and cytokines in the tumor microenvironment. J Immunol Res.

[43] Hu YW, Zhao JY, Li SF, Huang JL, Qiu YR, Ma X (2015). RP5-833A20.1/miR-382-5p/NFIA-dependent signal transduction pathway contributes to the regulation of cholesterol homeostasis and inflammatory reaction. Arterioscler Thromb Vasc Biol.

[44] Yan B, Yao J, Liu JY, Li XM, Wang XQ, Li YJ (2015). lncRNA-MIAT regulates microvascular dysfunction by functioning as a competing endogenous RNA. Circ Res.

[45] Wang WT, Ye H, Wei PP, Han BW, He B, Chen ZH (2016). LncRNAs H19 and HULC, activated by oxidative stress, promote cell migration and invasion in cholangiocarcinoma through a ceRNA manner. J Hematol Oncol.

[46] Lin KY, Ye H, Han BW, Wang WT, Wei PP, He B (2016). Genome-wide screen identified let-7c/miR-99a/miR-125b regulating tumor progression and stem-like properties in cholangiocarcinoma. Oncogene.

[47] Goydos JS, Brumfield AM, Frezza E, Booth A, Lotze MT, Carty SE (1998). Marked elevation of serum interleukin-6 in patients with cholangiocarcinoma: validation of utility as a clinical marker. Ann Surg.

[48] Johnson C, Han Y, Hughart N, McCarra J, Alpini G, Meng F (2012). Interleukin-6 and its receptor, key players in hepatobiliary inflammation and cancer. Transl Gastrointest Cancer.

[49] Rosen HR, Winkle PJ, Kendall BJ, Diehl DL (1997). Biliary interleukin-6 and tumor necrosis factor-alpha in patients undergoing endoscopic retrograde cholangiopancreatography. Dig Dis Sci.

[50] Hummel S, Van Aken H, Zarbock A (2014). Inhibitors of CXC chemokine receptor type 4: putative therapeutic approaches in inflammatory diseases. Curr Opin Hematol.

[51] Leelawat K, Leelawat S, Narong S, Hongeng S (2007). Roles of the MEK1/2 and AKT pathways in CXCL12/CXCR4 induced cholangiocarcinoma cell invasion. World J Gastroenterol.

[52] Ohira S, Sasaki M, Harada K, Sato Y, Zen Y, Isse K (2006). Possible regulation of migration of intrahepatic cholangiocarcinoma cells by interaction of CXCR4 expressed in carcinoma cells with tumor necrosis factor-alpha and stromal-derived factor-1 released in stroma. Am J Pathol.

[53] Matouk IJ, Raveh E, Abu-lail R, Mezan S, Gilon M, Gershtain E (2014). Oncofetal H19 RNA promotes tumor metastasis. Biochim Biophys Acta.

[54] Tay Y, Rinn J, Pandolfi PP (2014). The multilayered complexity of ceRNA crosstalk and competition. Nature.

[55] Xu J, Li Y, Lu J, Pan T, Ding N, Wang Z (2015). The mRNA related ceRNA-ceRNA landscape and significance across 20 major cancer types. Nucleic Acids Res.

[56] Ying L, Huang Y, Chen H, Wang Y, Xia L, Chen Y (2013). Downregulated MEG3 activates autophagy and increases cell proliferation in bladder cancer. Mol BioSyst.

[57] Quinonez SC, Innis JW (2014). Human HOX gene disorders. Mol Genet Metab.

[58] Shu Y, Wang B, Wang J, Wang JM, Zou SQ (2011). Identification of methylation profile of HOX genes in extrahepatic cholangiocarcinoma. World J Gastroenterol.

[59] Goyama S, Huang G, Kurokawa M, Mulloy JC (2015). Posttranslational modifications of RUNX1 as potential anticancer targets. Oncogene.

[60] Gabay M., Li Y., Felsher D. W. (2014). MYC Activation Is a Hallmark of Cancer Initiation and Maintenance. Cold Spring Harbor Perspectives in Medicine.

[61] Katoh M, Katoh M (2004). Human FOX gene family (review). Int J Oncol.

[62] Li Z, Huang H, Chen P, He M, Li Y, Arnovitz S (2012). miR-196b directly targets both HOXA9/MEIS1 oncogenes and FAS tumour suppressor in MLL-rearranged leukaemia. Nat Commun.

[63] Toska E, Roberts SG (2014). Mechanisms of transcriptional regulation by WT1 (Wilms' tumour 1). Biochem J.

[64] Garding A, Bhattacharya N, Claus R, Ruppel M, Tschuch C, Filarsky K (2013). Epigenetic upregulation of lncRNAs at 13q14.3 in leukemia is linked to the in Cis downregulation of a gene cluster that targets NF-kB. PLoS Genet.

[65] Liao XH, Wang JG, Li LY, Zhou DM, Ren KH, Jin YT (2016). Long intergenic non-coding RNA APOC1P1-3 inhibits apoptosis by decreasing alpha-tubulin acetylation in breast cancer. Cell Death Dis.

[66] Yang W, Li Y, Song X, Xu J, Xie J (2017). Genome-wide analysis of long noncoding RNA and mRNA co-expression profile in intrahepatic cholangiocarcinoma tissue by RNA sequencing. Oncotarget.

[67] Lv L, Wei M, Lin P, Chen Z, Gong P, Quan Z (2017). Integrated mRNA and lncRNA expression profiling for exploring metastatic biomarkers of human intrahepatic cholangiocarcinoma. Am J Cancer Res.

[68] Zhao S, Wang J, Qin C (2014). Blockade of CXCL12/CXCR4 signaling inhibits intrahepatic cholangiocarcinoma progression and metastasis via inactivation of canonical Wnt pathway. J Exp Clin Cancer Res.

