# Supplementary material for: Global identification and characterization of lncRNAs that control inflammation in malignant cholangiocytes
Source: BMC Genomics. 2018 Oct 11;19:735. doi: 10.1186/s12864-018-5133-8 (PMC6180422; doi:10.1186/s12864-018-5133-8)
Supplement: Supplementary file 1 — Table S1. Patient demographics and clinicopathologic features. (DOCX 22 kb) [file 12864_2018_5133_MOESM1_ESM.docx]

**Table S1. Patient demographics and clinicopathologic features.**

|  | trainning set | | Validation set | |
| --- | --- | --- | --- | --- |
| Characteristics | Intra-hepatic CCA | Extra-hepatic CCA | Intra-hepatic CCA | Extra-hepatic CCA |
|  | n = 13 (52.0%) | n = 12 (48.0%) | n=3(27.3%) | n=8(72.7%) |
| Age (years, median and range) | 59 (45-66) | 57 (33-76) | 69(62-79) | 57.5(47-70) |
| Sex (%) |  |  |  |  |
| Male | 7 (53.8) | 7 (58.3) | 2(66.7) | 4（50） |
| Female | 6 (46.2) | 5 (41.7) | 1（33.3） | 4（50） |
| HBV+ (%) | 7 (53.8) | 5 (41.7) | 3（100） | 3（37.5） |
| Tumor size (cm, median and range) | 5.0 (2.4-10.0) | 3.0 (1.2-4.6) | 6（2.2-12） | 4（1.2-8.0） |
| CA19-9 (U/ml, median and range) | 2098.3 (55.9-18706.0) | 160.6 (0.9-32394.0) | 213.2（103.4-11068） | 2086（0.6-9330） |
| Differentiation stage (%) | | |  |  |
| Well | 6 (46.1) | 6 (50.0) | 0（0） | 2（25） |
| Moderately | 2 (15.4) | 2 (16.7) | 3（100） | 5（62.5） |
| Moderately-poorly | 4 (30.8) | 2 (16.7) | 0（0） | 1（12.5） |
| Poorly | 1 (8.7) | 2 (16.7) | 0（0） | 0（0） |
