# Supplementary material for: Global identification and characterization of lncRNAs that control inflammation in malignant cholangiocytes
Source: BMC Genomics. 2018 Oct 11;19:735. doi: 10.1186/s12864-018-5133-8 (PMC6180422; doi:10.1186/s12864-018-5133-8)
Supplement: Supplementary file 2 — Table S2. Primer sequence for qPCR and vector construction. (DOCX 23 kb) [file 12864_2018_5133_MOESM2_ESM.docx]

**Table S2. Primer sequence for qPCR and vector construction.**

| Primer Name | Sequence 5' to 3' |
| --- | --- |
| HULC-Forward | GCAAGCCAGGAAGAGTCGTC |
| HULC-Reverse | GCTGTGCTTAGTTTATTGCCAGG |
| H19-Forward | TTCCAGGCAGAAAGAGCAAGAGGGC |
| H19-Reverse | AGACGTCCTGCTGCAACTCCCCGAG |
| RP11-528A4.2-Forward | GAACCGGCTCCTCTATCCCC |
| RP11-528A4.2-Reverse | AAACGGTCTGTGCTCTGCGT |
| LPAL2-Forward | AGATGCCGATACAGGCCCTT |
| LPAL2-Reverse | CACAGCTCTGGCTTTCCCAG |
| C3P1-Forward | CAGACCGTGAGTGCCAAACA |
| C3P1-Reverse | AAAGGCCAGAATGCGGAAGG |
| APOC1P1-Forward | GCTCCAGAAGTCTCCAACCC |
| APOC1P1-Reverse | TGAGTCAGTCTTGAGTTTCTCCTTC |
| RP11-370B11.3-Forward | GTGAAGCTCCATATGTCCCACA |
| RP11-370B11.3-Reverse | CCTTCCCTCCATGTATCCCCA |
| AC005550.3-Forward | GGGTTCTGGCTTCGGATAGTG |
| AC005550.3-Reverse | TTTTGTGAAGAGTCCAGCAGGC |
| PVT1-Forward | ACCCTGCCCATGCCATAGAT |
| PVT1-Reverse | GTGGGGCTTGTGAATCTGGG |
| CTC-505O3.2-Forward | TCCCTAGAGCCCAACCTTGC |
| CTC-505O3.2-Reverse | GCTCATTGGCTACCCTGACC |
| RP11-622A1.2-Forward | GCTACCATCAACCCAGTCTCC |
| RP11-622A1.2-Reverse | TTGGAATGGCACTGGCTGTT |
| LIT3594-Forward | ACGTCAGCGTCACATTGGC |
| LIT3594-Reverse | CTGCACAGTCCCTTTGACGA |
| GAPDH-Forward | GCTGAACGGGAAGCTCACTG |
| GAPDH-Reverse | GTGCTCAGTGTAGCCCAGGA |
| *Apoa2-* Forward | CTTGTCAGGTCAGCAGGAA |
| Apoa2- Reverse | TCACTTAGCCGCAGGAG |
| CFH- Forward | CAGCCACAGATGAGTTAGG |
| CFH- Reverse | CCAGATTGCCAGTTCAGG |
| KNG1- Forward | TCAACCACTGGGAATGATCTCAC |
| KNG1- Reverse | TCGCAGGACCTTAGGTGACTA |
| MBL2- Forward | CATCAACGGCTTCCCAGGC |
| MBL2- Reverse | TGGGCTGGCAAGACAACTATTAG |
| ORM1- Forward | TTGCGCATTCCCAAGTCA |
| ORM1- Reverse | CAGTGGCTCACACTTATCCTTTTTC |
| Orm2- Forward | ATCTCTTCCAAGCCCTGGTG |
| *Orm2*- Reverse | ATTGGTGATAGGGTCGCCTATGGT |
| SAA4- Forward | GTGATGGGAGTCAGCAGTGA |
| SAA4- Reverse | CTTTGGGCAGCCTCGTAGT |
| CCL16- Forward | CCACCTGCTGCCTGAAGTAT |
| CCL16- Reverse | GTTGGTGCAGACTTCTCGGT |
| APOC1P1-full-F: | GCGGCCGCCAACCAAGCCCTCCAGCA |
| APOC1P1-full-R: | TCTAGAGCCTCAGCCTCCCGAATAG |
