# Supplementary figures and images for: Global identification and characterization of lncRNAs that control inflammation in malignant cholangiocytes
Source: BMC Genomics. 2018 Oct 11;19:735. doi: 10.1186/s12864-018-5133-8 (PMC6180422; doi:10.1186/s12864-018-5133-8)

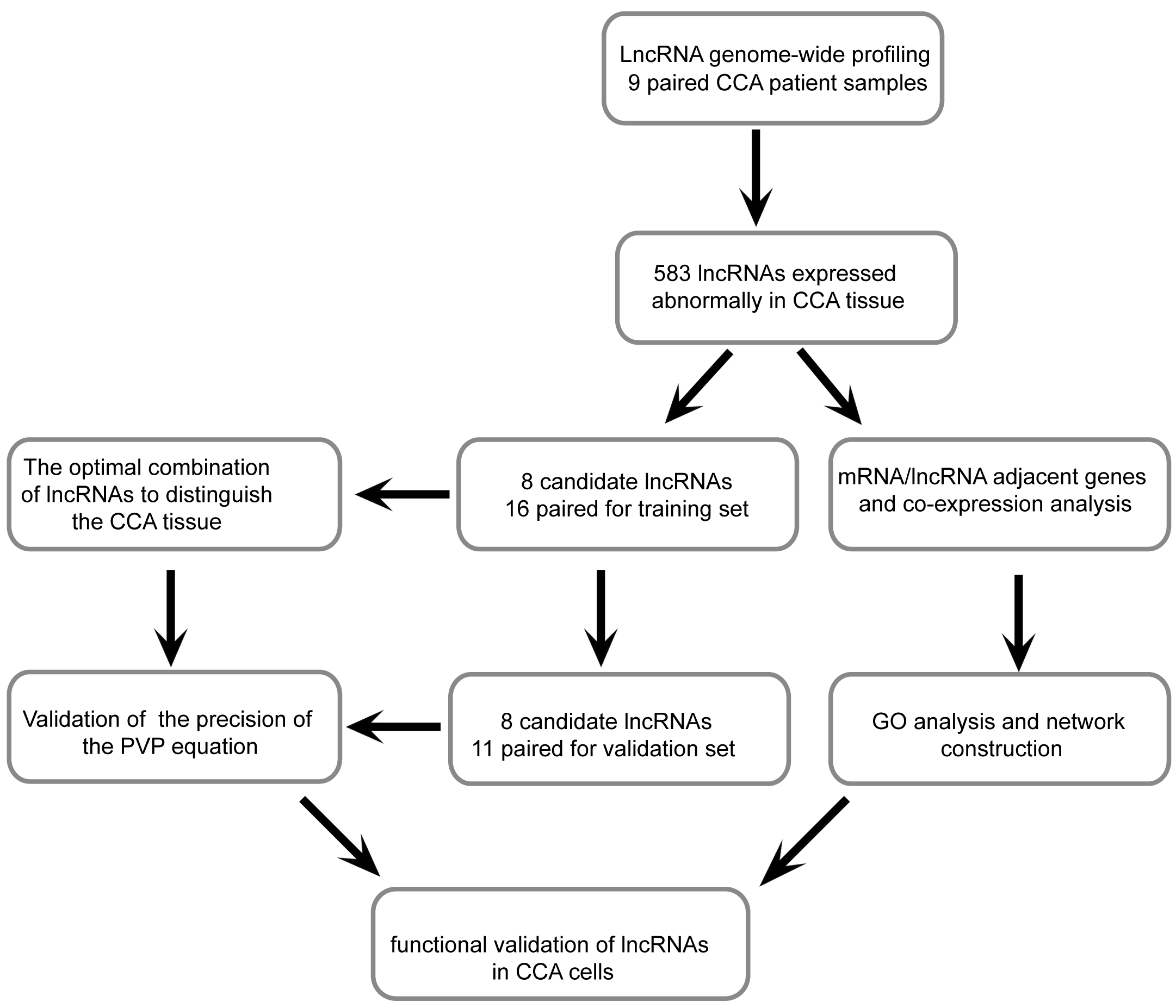

Supplement: Supplementary file 3 — Figure S1. The framework of the study. (TIF 450 kb) [file 12864_2018_5133_MOESM3_ESM.tif]

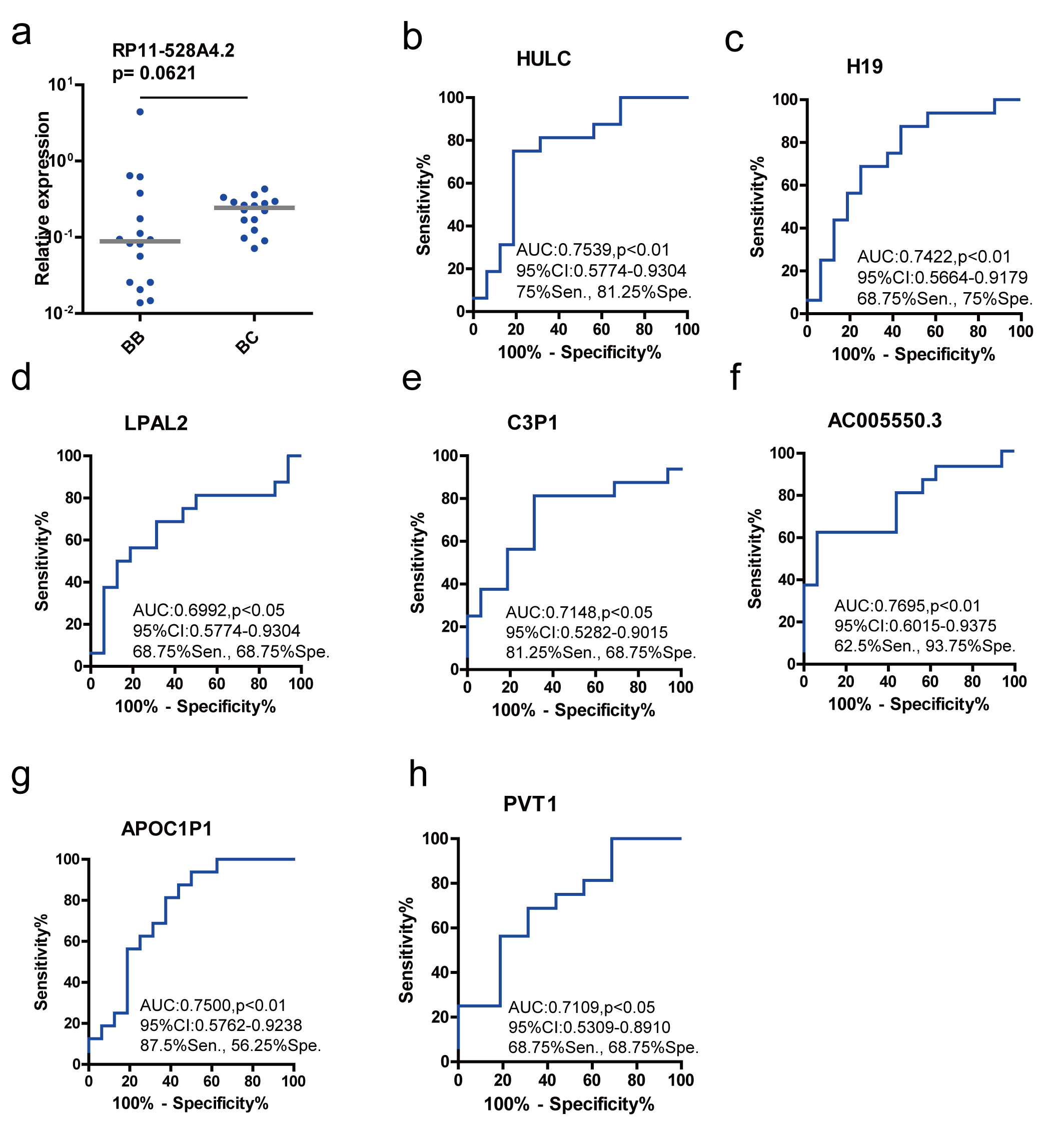

Supplement: Supplementary file 4 — Figure S2. Assessment of the diagnostic accuracy of these special lncRNAs for Cholangiocarcinoma. (a) the expression of RP11-528A4.2. Diagnostic value of lncRNAs for Cholangiocarcinoma: HULC(b), H19(c), LPAL2(d), C3P1(e), AC005550.3(f), APOC1P1(g), PVT1(h),and sensitivity: Sen. for short, specificity: Spe. for short. (TIF 638 kb) [file 12864_2018_5133_MOESM4_ESM.tif]

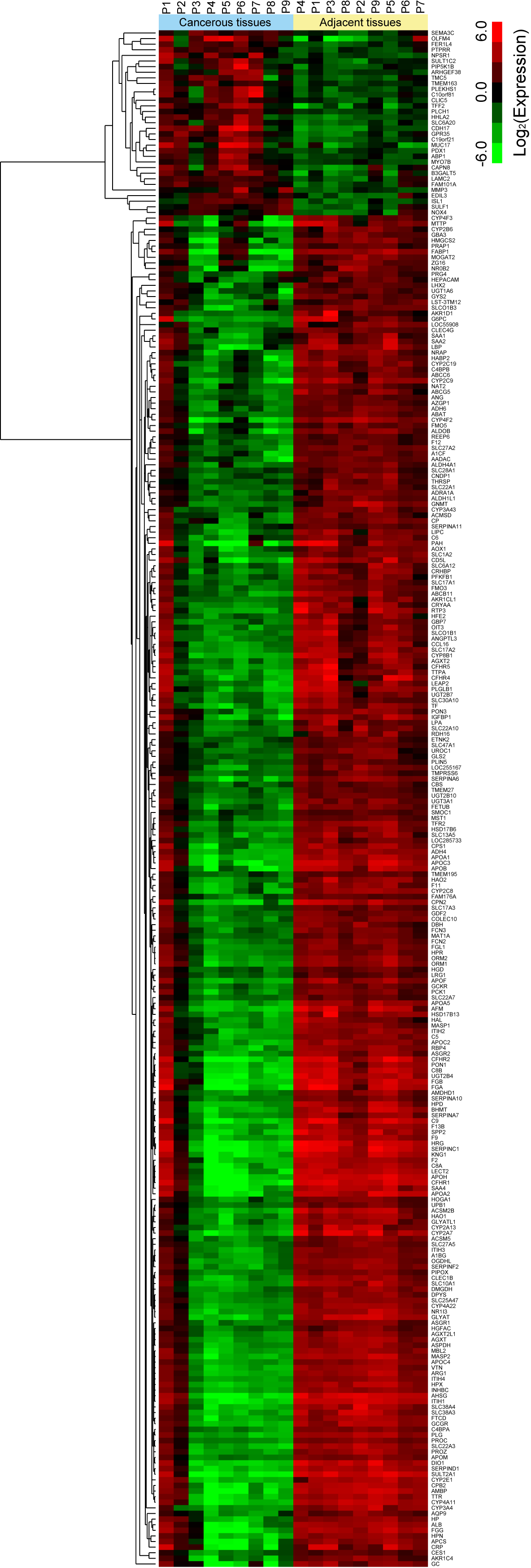

Supplement: Supplementary file 8 — Figure S3. Cluster analysis of mRNA expression in cancerous and adjacent tissues of cholangiocarcinoma patients (TIF 1712 kb) [file 12864_2018_5133_MOESM8_ESM.tif]

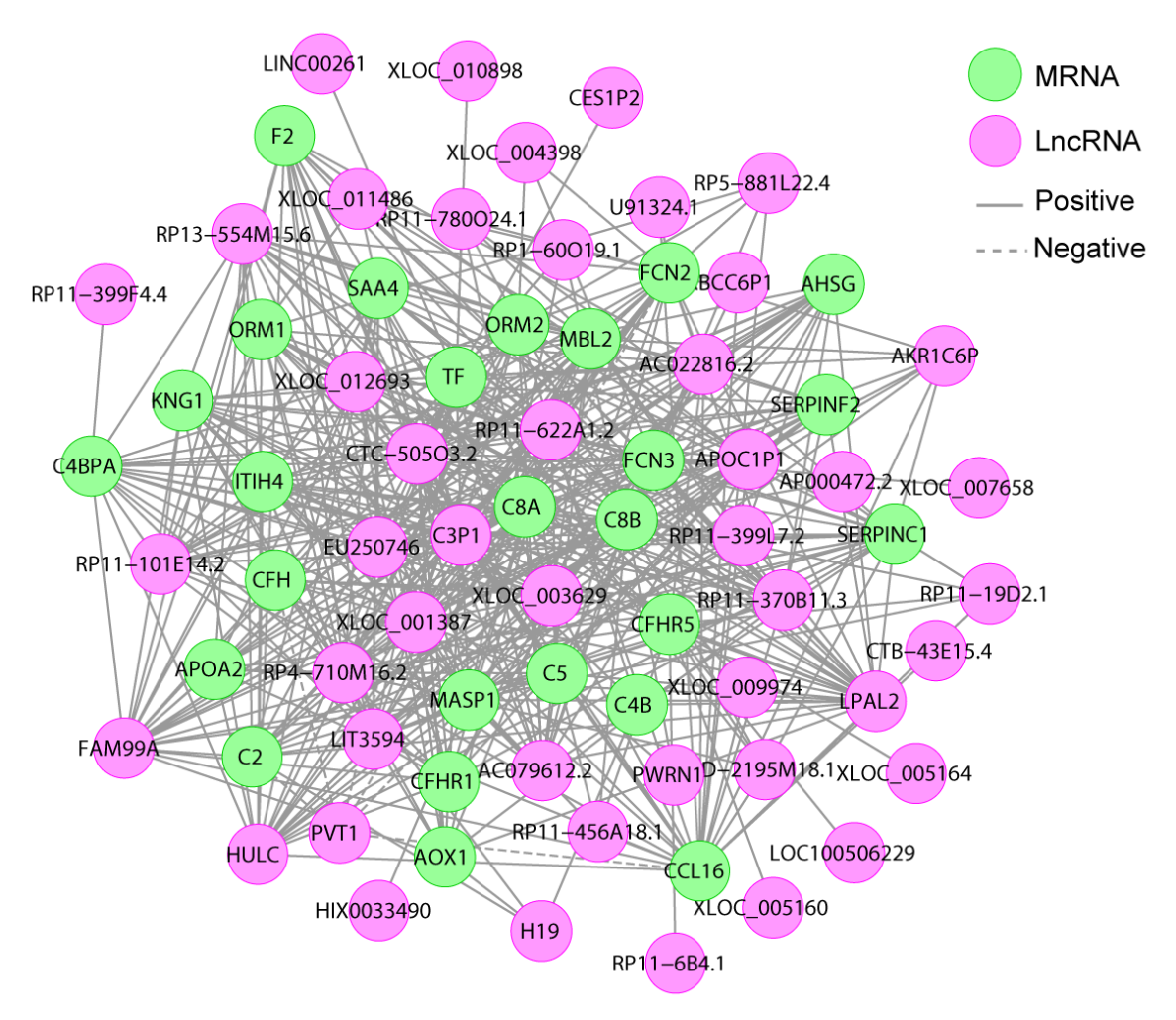

Supplement: Supplementary file 10 — Figure S4. The network of dysregulated lncRNA and the co-expressed inflammatory genes. The dysregulated lncRNAs (red nodes) that were highly co-expressed (Pearson correlation r > 0.85) with inflammatory genes (green nodes). (TIF 740 kb) [file 12864_2018_5133_MOESM10_ESM.tif]

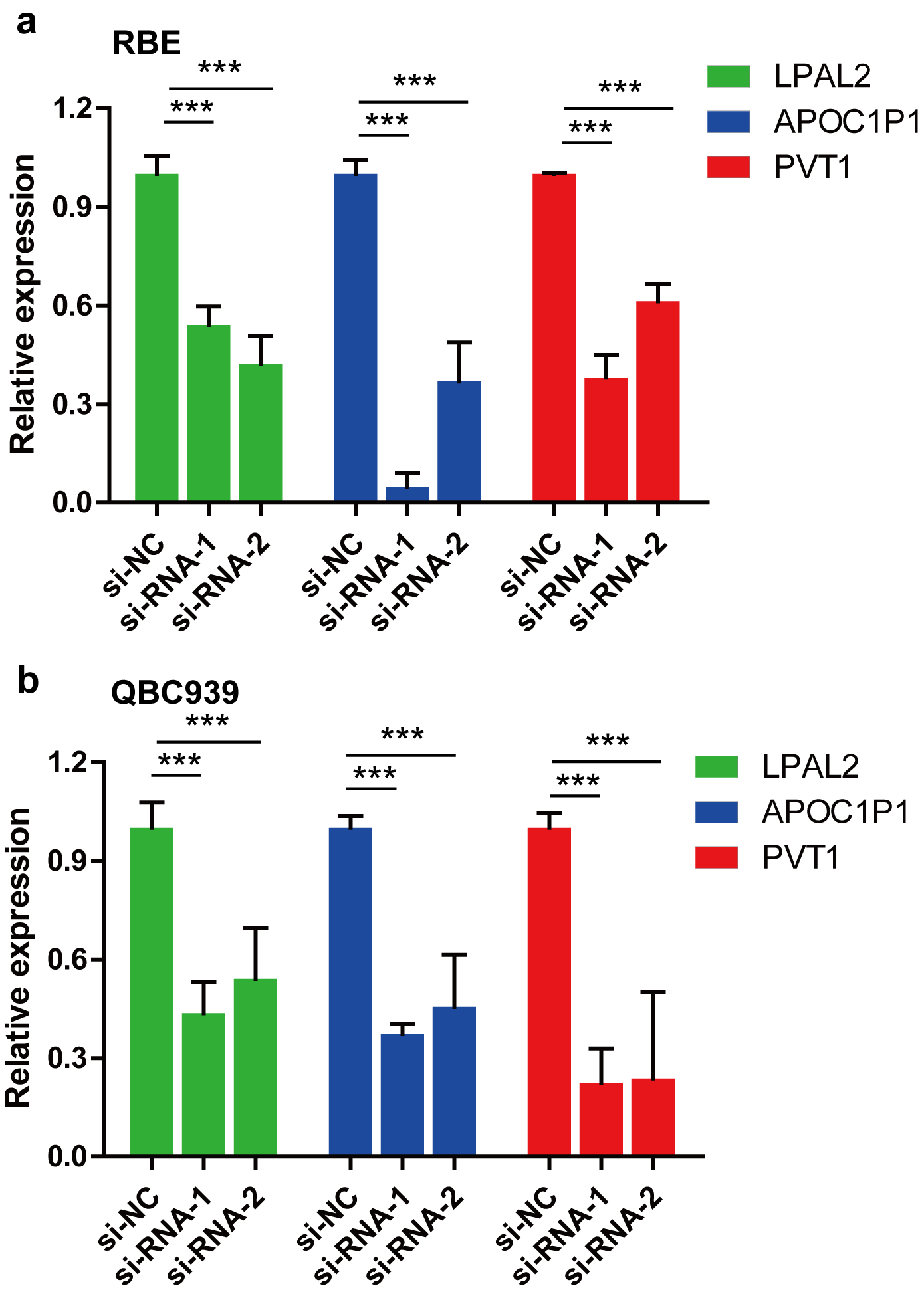

Supplement: Supplementary file 11 — Figure S5. The qpcR test for knockdown efficiency of the selected lncRNAs targeted by siRNAs in CCA cell lines. (a) RBE and (b)QBC939 cells. (TIF 537 kb) [file 12864_2018_5133_MOESM11_ESM.tif]

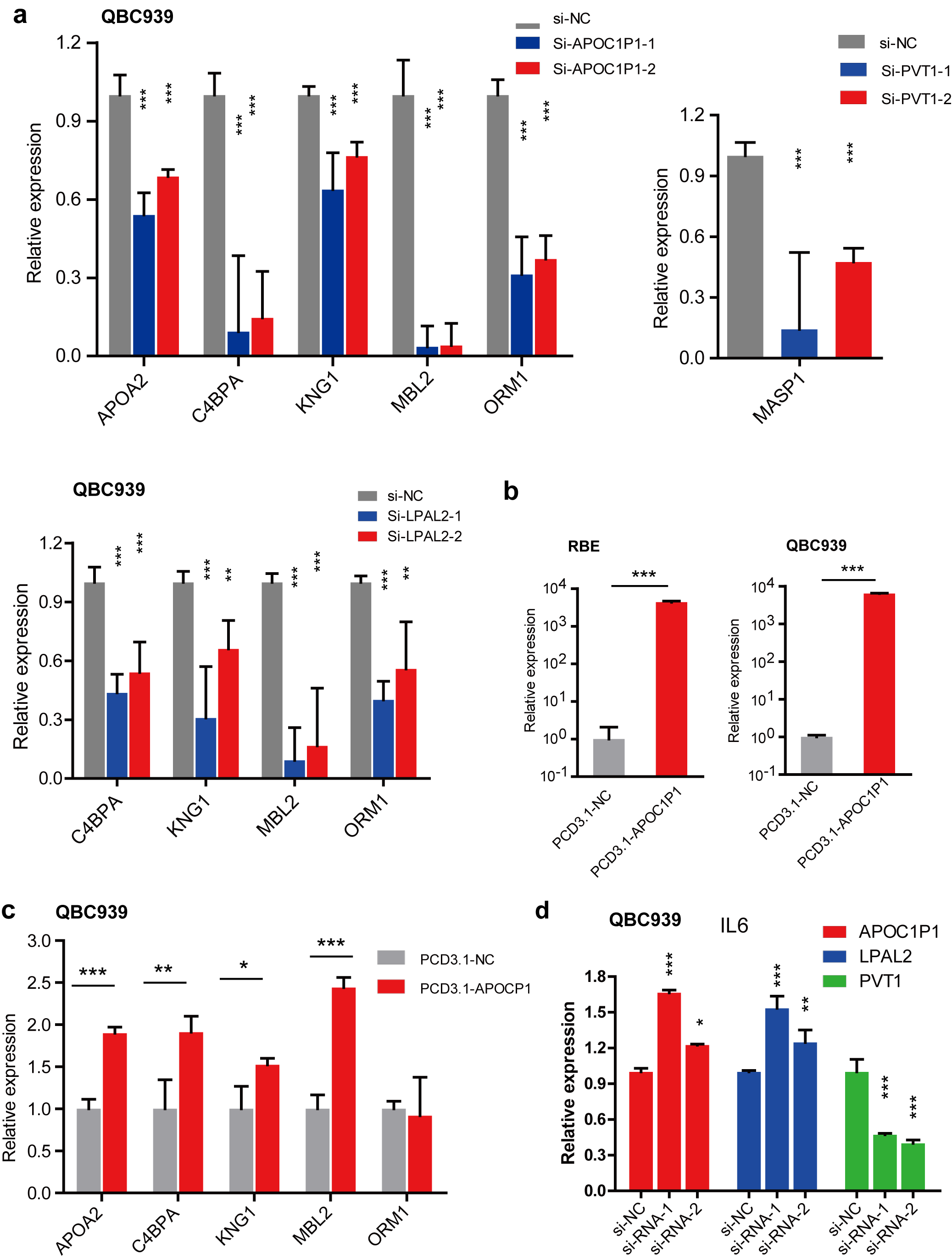

Supplement: Supplementary file 12 — Figure S6. Functional validation of selected lncRNAs in malignant cholangiocytes. (a) A set of the potentially targeted gens, like C4BPA, KNG1, MASP1, MBL2, etc., are significantly downregulated when APOC1P1, PVT1, and LPAL2 were knocked down by the siRNAs in CCA cells. (b) The qpcR test for the IL6 mRNA under the knockdown the selected lncRNAs targeted by siRNAs in QBC939. (c) The qpcR test for overexpression efficiency of APOC1P1 in CCA cell lines. (d) A set of the potentially targeted gens, like C4BPA, KNG1, MASP1, MBL2, etc., are significantly upregulated when APOC1P1 were overexpressed in QBC939. (TIF 929 kb) [file 12864_2018_5133_MOESM12_ESM.tif]

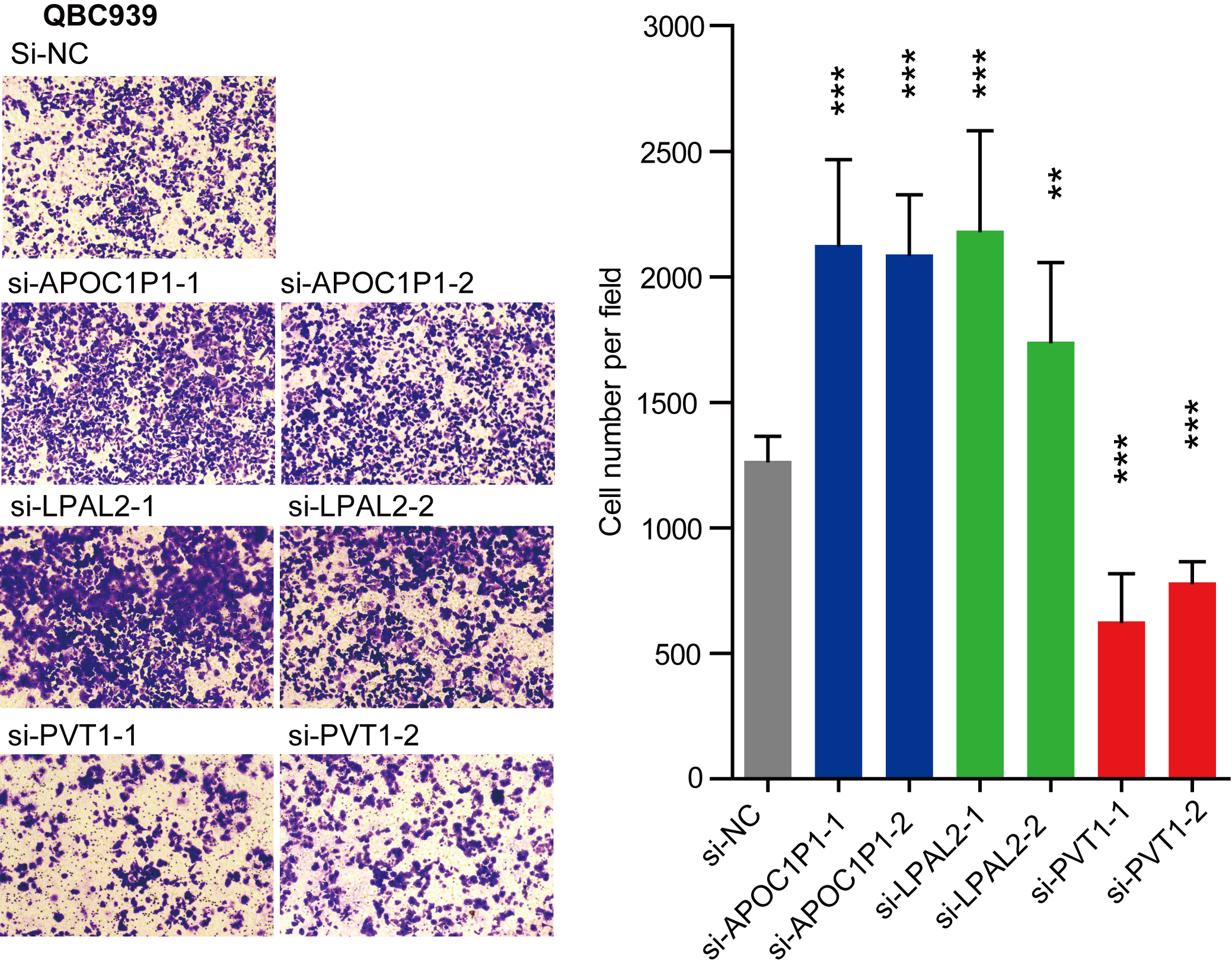

Supplement: Supplementary file 13 — Figure S7. Migration of QBC939 knocking down of selected lncRNAs in malignant cholangiocytes. QBC939 Cells were counted in ten fields for triplicate membranes at 10× magnification. Five random sights in each sample were selected to analyze cell count, and the triplicate experiments were analyzed by Mean ± SD, p value< 0.001, ***, < 0.01, **. (TIF 3990 kb) [file 12864_2018_5133_MOESM13_ESM.tif]
