# Supplementary material for: Global identification and characterization of lncRNAs that control inflammation in malignant cholangiocytes
Source: BMC Genomics. 2018 Oct 11;19:735. doi: 10.1186/s12864-018-5133-8 (PMC6180422; doi:10.1186/s12864-018-5133-8)
Supplement: Supplementary file 5 — Table S3. Adjacent genes of dysregulated lncRNAs. (DOCX 80 kb) [file 12864_2018_5133_MOESM5_ESM.docx]

**Table S3. Adjacent genes of dysregulated lncRNAs.**

| Gene_symbol | Chromosome | lncRNA_symbol | lncRNA_start | lncRNA_end | lncRNA_strand |
| --- | --- | --- | --- | --- | --- |
| ARHGEF2 | chr1 | ENST00000454974 | 155966113 | 155972355 | - |
| BCAR3 | chr1 | ASO3817 | 94057634 | 94068376 | + |
| C1orf68 | chr1 | AF005081 | 152692385 | 152692799 | + |
| C8A | chr1 | RNZ2250 | 57354732 | 57354932 | + |
| CAMSAP2 | chr1 | RNZ3995 | 200704279 | 200704556 | + |
| CEP85 | chr1 | uc001blq.2 | 26551810 | 26556331 | + |
| DISP1 | chr1 | ENST00000439440 | 222988452 | 223000924 | + |
| ERRFI1 | chr1 | ENST00000423628 | 8066073 | 8066784 | - |
| F3 | chr1 | XLOC_000302 | 95007607 | 95008571 | + |
| FGGY | chr1 | RNZ1812 | 60196873 | 60197150 | + |
| GPR89C | chr1 | NR_003377 | 145924387 | 145942619 | - |
| GPR89C | chr1 | RNZ3245 | 146823120 | 146823397 | + |
| HSD11B1 | chr1 | RNZ4229 | 209881764 | 209882008 | + |
| LAPTM5 | chr1 | NR_034182 | 31191618 | 31199593 | + |
| LOC100288142 | chr1 | ENST00000439352 | 144593735 | 144597070 | + |
| LOC100288142 | chr1 | NR_003377 | 145924387 | 145942619 | - |
| LPHN2 | chr1 | RNZ2566 | 82049589 | 82049829 | + |
| LPHN2 | chr1 | AK026847 | 82458559 | 82462214 | + |
| MATN1 | chr1 | NR_034182 | 31191618 | 31199593 | + |
| MT1HL1 | chr1 | uc001hyk.1 | 237167402 | 237167718 | - |
| NBPF10 | chr1 | NR_003377 | 145924387 | 145942619 | - |
| NBPF24 | chr1 | RNZ3245 | 146823120 | 146823397 | + |
| NBPF8 | chr1 | ENST00000439352 | 144593735 | 144597070 | + |
| NBPF8 | chr1 | NR_003377 | 145924387 | 145942619 | - |
| NBPF8 | chr1 | RNZ3245 | 146823120 | 146823397 | + |
| NBPF9 | chr1 | ENST00000439352 | 144593735 | 144597070 | + |
| NBPF9 | chr1 | NR_003377 | 145924387 | 145942619 | - |
| NBPF9 | chr1 | RNZ3245 | 146823120 | 146823397 | + |
| NID1 | chr1 | XLOC_000628 | 236120926 | 236138865 | + |
| PBX1 | chr1 | uc.40+ | 164637961 | 164638208 | + |
| PCSK9 | chr1 | ASO2233 | 55531370 | 55534931 | + |
| PPIAL4B | chr1 | ENST00000439352 | 144593735 | 144597070 | + |
| PPIAL4B | chr1 | NR_003377 | 145924387 | 145942619 | - |
| PPIAL4B | chr1 | RNZ3245 | 146823120 | 146823397 | + |
| PROX1 | chr1 | ENST00000451396 | 214139236 | 214159496 | + |
| ROR1 | chr1 | RNZ2412 | 64283086 | 64283365 | + |
| SLAMF9 | chr1 | ENST00000423943 | 159931007 | 159948851 | + |
| SLC44A3 | chr1 | uc001dqu.2 | 95123089 | 95285834 | - |
| SSR2 | chr1 | ENST00000454974 | 155966113 | 155972355 | - |
| USP24 | chr1 | ASO2233 | 55531370 | 55534931 | + |
| WLS | chr1 | ENST00000434072 | 68604039 | 68607471 | + |
| ZNF281 | chr1 | uc010ppi.1 | 200380927 | 200444641 | + |
| ANAPC1 | chr2 | ENST00000409139 | 87754947 | 87906324 | + |
| ANAPC1 | chr2 | XLOC_002197 | 88436734 | 88438049 | - |
| ANAPC1 | chr2 | ENST00000421951 | 89065323 | 89106126 | + |
| ANAPC1 | chr2 | uc002stt.2 | 91807794 | 91847975 | - |
| ANAPC1 | chr2 | XLOC_001583 | 98081682 | 98091049 | - |
| ANAPC1 | chr2 | ENST00000448595 | 99385423 | 99388543 | - |
| ANAPC1 | chr2 | ENST00000449772 | 105028684 | 105030466 | - |
| ANAPC1 | chr2 | ENST00000415627 | 105990549 | 105992540 | + |
| ANAPC1 | chr2 | XLOC_001619 | 108145942 | 108172782 | + |
| ANAPC1 | chr2 | NR_027145 | 110656008 | 111230652 | - |
| ANAPC1 | chr2 | uc002the.2 | 111965358 | 112252692 | - |
| BCL11A | chr2 | uc002sag.2 | 60751632 | 60755350 | - |
| C2orf50 | chr2 | ENST00000447433 | 11264344 | 11272896 | - |
| C2orf72 | chr2 | HIT000066977.10 | 231916754 | 231917352 | + |
| C2orf91 | chr2 | ENST00000427054 | 42158738 | 42160605 | - |
| CPS1 | chr2 | NR_002763 | 211482294 | 211484599 | + |
| FABP1 | chr2 | XLOC_002197 | 88436734 | 88438049 | - |
| FHL2 | chr2 | ENST00000415627 | 105990549 | 105992540 | + |
| FLJ30838 | chr2 | RNZ18402 | 58749313 | 58749552 | + |
| FLJ33534 | chr2 | ENST00000447433 | 11264344 | 11272896 | - |
| GPR113 | chr2 | XLOC_001401 | 26526697 | 26529191 | + |
| HAAO | chr2 | AK123057 | 42990224 | 42992816 | + |
| HAAO | chr2 | XLOC_002066 | 43028633 | 43038968 | - |
| HOXD1 | chr2 | ENST00000413969 | 177041183 | 177053686 | - |
| HOXD3 | chr2 | ENST00000413969 | 177041183 | 177053686 | - |
| IGKV3-11 | chr2 | ENST00000409139 | 87754947 | 87906324 | + |
| IGKV3-11 | chr2 | XLOC_002197 | 88436734 | 88438049 | - |
| IGKV3-11 | chr2 | ENST00000421951 | 89065323 | 89106126 | + |
| LIMS3 | chr2 | NR_027145 | 110656008 | 111230652 | - |
| LIMS3-LOC440895 | chr2 | NR_027145 | 110656008 | 111230652 | - |
| LOC100287010 | chr2 | ENST00000449772 | 105028684 | 105030466 | - |
| LOC151009 | chr2 | NR_027145 | 110656008 | 111230652 | - |
| LOC541471 | chr2 | uc002the.2 | 111965358 | 112252692 | - |
| LOC654342 | chr2 | uc002stt.2 | 91807794 | 91847975 | - |
| MALL | chr2 | NR_027145 | 110656008 | 111230652 | - |
| MEIS1 | chr2 | RNZ18620 | 66697968 | 66698284 | + |
| MTA3 | chr2 | AK123057 | 42990224 | 42992816 | + |
| NCKAP5 | chr2 | ENST00000432414 | 134023766 | 134042334 | + |
| NPHP1 | chr2 | NR_027145 | 110656008 | 111230652 | - |
| OXER1 | chr2 | AK123057 | 42990224 | 42992816 | + |
| PLGLB2 | chr2 | ENST00000409139 | 87754947 | 87906324 | + |
| PSMD1 | chr2 | HIT000066977.10 | 231916754 | 231917352 | + |
| RGPD1 | chr2 | ENST00000409139 | 87754947 | 87906324 | + |
| RGPD5 | chr2 | NR_027145 | 110656008 | 111230652 | - |
| RGPD5 | chr2 | uc002the.2 | 111965358 | 112252692 | - |
| RGPD6 | chr2 | NR_027145 | 110656008 | 111230652 | - |
| RMND5A | chr2 | ENST00000409139 | 87754947 | 87906324 | + |
| RUFY4 | chr2 | NR_002712 | 218923877 | 218926013 | - |
| SLC19A3 | chr2 | uc002vpj.2 | 228549926 | 228582745 | - |
| SRD5A2 | chr2 | ENST00000405650 | 31747549 | 31806136 | - |
| UGT1A1 | chr2 | NR_037694 | 234662961 | 234663991 | - |
| UGT1A10 | chr2 | NR_037694 | 234662961 | 234663991 | - |
| UGT1A3 | chr2 | NR_037694 | 234662961 | 234663991 | - |
| UGT1A4 | chr2 | NR_037694 | 234662961 | 234663991 | - |
| UGT1A5 | chr2 | NR_037694 | 234662961 | 234663991 | - |
| UGT1A6 | chr2 | NR_037694 | 234662961 | 234663991 | - |
| UGT1A7 | chr2 | NR_037694 | 234662961 | 234663991 | - |
| UGT1A8 | chr2 | NR_037694 | 234662961 | 234663991 | - |
| UGT1A9 | chr2 | NR_037694 | 234662961 | 234663991 | - |
| APEH | chr3 | uc001azm.3 | 49721912 | 49722416 | - |
| CHRD | chr3 | XLOC_002948 | 184096017 | 184097565 | + |
| CP | chr3 | XLOC_002865 | 148942374 | 148944127 | + |
| CTDSPL | chr3 | ENST00000438136 | 37795179 | 37903229 | - |
| EPHB1 | chr3 | RNZ23992 | 134513868 | 134514107 | + |
| EPHB1 | chr3 | XLOC_003263 | 134979869 | 135158671 | - |
| GNAI2 | chr3 | ENST00000456338 | 50242700 | 50256112 | + |
| GNAI2 | chr3 | ENST00000439898 | 50297734 | 50300790 | + |
| GNAT1 | chr3 | ENST00000456338 | 50242700 | 50256112 | + |
| HGD | chr3 | RNZ23849 | 120363936 | 120364266 | + |
| HHLA2 | chr3 | ASO1844 | 108106442 | 108108128 | + |
| IQSEC1 | chr3 | ENST00000499738 | 13054480 | 13057307 | + |
| ITGA9 | chr3 | ENST00000438136 | 37795179 | 37903229 | - |
| KCTD6 | chr3 | XLOC_002675 | 58419600 | 58470480 | + |
| LOC647323 | chr3 | ENST00000449350 | 193727964 | 193788741 | - |
| MBNL1 | chr3 | NR_027037 | 151980404 | 151987415 | - |
| MBNL1 | chr3 | NR_027038 | 151980404 | 151987415 | - |
| MECOM | chr3 | RNZ24353 | 168919598 | 168919836 | + |
| MECOM | chr3 | RNZ24358 | 168982583 | 168982790 | + |
| MECOM | chr3 | RNZ24362 | 169072712 | 169072912 | + |
| MST1 | chr3 | uc001azm.3 | 49721912 | 49722416 | - |
| MYH15 | chr3 | ASO1844 | 108106442 | 108108128 | + |
| PDHB | chr3 | XLOC_002675 | 58419600 | 58470480 | + |
| POLR2H | chr3 | XLOC_002948 | 184096017 | 184097565 | + |
| PXK | chr3 | XLOC_002675 | 58419600 | 58470480 | + |
| RFTN1 | chr3 | RNZ22513 | 16518419 | 16518696 | + |
| RNF123 | chr3 | uc001azm.3 | 49721912 | 49722416 | - |
| SEMA3B | chr3 | ENST00000439898 | 50297734 | 50300790 | + |
| SLC2A2 | chr3 | ASO1952 | 170712264 | 170713500 | + |
| SLC38A3 | chr3 | ENST00000456338 | 50242700 | 50256112 | + |
| THPO | chr3 | XLOC_002948 | 184096017 | 184097565 | + |
| ZBTB20 | chr3 | RNZ23677 | 114135838 | 114136113 | + |
| ADH1A | chr4 | ENST00000500358 | 100010007 | 100222513 | + |
| ADH1B | chr4 | ENST00000500358 | 100010007 | 100222513 | + |
| ADH4 | chr4 | ENST00000500358 | 100010007 | 100222513 | + |
| ADH5 | chr4 | ENST00000500358 | 100010007 | 100222513 | + |
| ADH6 | chr4 | ENST00000500358 | 100010007 | 100222513 | + |
| AFM | chr4 | RP11-622A1.2 | 74374519 | 74399845 | + |
| CEP135 | chr4 | HIT000242487.9 | 56828899 | 56829220 | + |
| CYP4V2 | chr4 | XLOC_003821 | 187137129 | 187137707 | + |
| F11 | chr4 | NR_033900 | 187207251 | 187422212 | - |
| IRF2 | chr4 | XLOC_004205 | 185286340 | 185303530 | - |
| LOC728040 | chr4 | RP11-622A1.2 | 74374519 | 74399845 | + |
| LPHN3 | chr4 | uc003hcu.1 | 62941883 | 62944051 | + |
| MGC45800 | chr4 | ENST00000315302 | 183062802 | 183065619 | - |
| PDGFRA | chr4 | RNZ25264 | 54729858 | 54730075 | + |
| QDPR | chr4 | XLOC_003471 | 17484657 | 17487209 | + |
| SMIM14 | chr4 | uc003gun.1 | 39548920 | 39551206 | - |
| TENM3 | chr4 | ENST00000315302 | 183062802 | 183065619 | - |
| UGT8 | chr4 | XLOC_003653 | 115602946 | 115611114 | + |
| ZNF827 | chr4 | RNZ26024 | 146855638 | 146855838 | + |
| ABLIM3 | chr5 | RNZ27942 | 148574976 | 148575244 | + |
| CCNG1 | chr5 | uc003lzd.1 | 162875115 | 162877423 | - |
| CDC25C | chr5 | RNZ27716 | 137674323 | 137675451 | + |
| CDO1 | chr5 | ENST00000499037 | 115150891 | 115152356 | + |
| DBN1 | chr5 | ENST00000513271 | 176875052 | 176879142 | - |
| EBF1 | chr5 | AK021803 | 158499438 | 158500620 | - |
| EBF1 | chr5 | ENST00000523301 | 158527629 | 158544486 | + |
| FAM53C | chr5 | RNZ27716 | 137674323 | 137675451 | + |
| FLJ16171 | chr5 | RNZ28606 | 174344658 | 174344955 | + |
| GRK6 | chr5 | ENST00000513271 | 176875052 | 176879142 | - |
| LOC728554 | chr5 | XLOC_005099 | 177387461 | 177392855 | - |
| MEF2C | chr5 | ENST00000509179 | 88185190 | 88261689 | + |
| MYOT | chr5 | NR_002713 | 137136881 | 137146439 | + |
| NPY6R | chr5 | NR_002713 | 137136881 | 137146439 | + |
| NUDCD2 | chr5 | uc003lzd.1 | 162875115 | 162877423 | - |
| PMCHL2 | chr5 | uc011csc.1 | 70671790 | 70681768 | + |
| PPP1R2P3 | chr5 | NR_002168 | 156277548 | 156279539 | + |
| PRR7 | chr5 | ENST00000513271 | 176875052 | 176879142 | - |
| SMAD5 | chr5 | HIT000067435.11 | 135465195 | 135470579 | - |
| TENM2 | chr5 | RNZ28297 | 166951892 | 166952166 | + |
| TENM2 | chr5 | RNZ28305 | 167054089 | 167054289 | + |
| ZNF354A | chr5 | XLOC_005102 | 178121720 | 178134039 | - |
| C6orf70 | chr6 | NR_026780 | 170190168 | 170202969 | + |
| CUTA | chr6 | RNZ29059 | 33379104 | 33379345 | + |
| ELOVL2 | chr6 | uc003mzq.1 | 11044923 | 11079377 | + |
| GSTA2 | chr6 | NR_033760 | 52604260 | 52609957 | - |
| HEY2 | chr6 | ENST00000451660 | 125899712 | 126070332 | - |
| KHDRBS2 | chr6 | XLOC_005755 | 62340137 | 62390954 | - |
| KIFC1 | chr6 | RNZ29059 | 33379104 | 33379345 | + |
| LRRC16A | chr6 | ASO2281 | 25593014 | 25593574 | - |
| MARCKS | chr6 | ENST00000434296 | 114191161 | 114191899 | - |
| MRPS18B | chr6 | RNZ29018 | 30583811 | 30584192 | + |
| PHF1 | chr6 | RNZ29059 | 33379104 | 33379345 | + |
| PPP1R10 | chr6 | RNZ29018 | 30583811 | 30584192 | + |
| SLC17A1 | chr6 | HIT000328221.9 | 25798603 | 25798901 | + |
| SLC17A1 | chr6 | HIT000325594.4 | 25818263 | 25818644 | - |
| SYCP2L | chr6 | ENST00000500710 | 10743565 | 10747802 | - |
| SYNGAP1 | chr6 | RNZ29059 | 33379104 | 33379345 | + |
| TAGAP | chr6 | ASO3608 | 159463302 | 159469735 | + |
| TCP10 | chr6 | AK000257 | 167762738 | 167764966 | - |
| TMEM14B | chr6 | ENST00000500710 | 10743565 | 10747802 | - |
| TTLL2 | chr6 | AK000257 | 167762738 | 167764966 | - |
| ABHD11 | chr7 | NR_026690 | 73149398 | 73150330 | + |
| AZGP1 | chr7 | NR_036679 | 99578384 | 99581860 | + |
| AZGP1P1 | chr7 | NR_036679 | 99578384 | 99581860 | + |
| C7orf10 | chr7 | RNZ30935 | 40786561 | 40786772 | + |
| CADPS2 | chr7 | NR_036484 | 121943711 | 121950131 | + |
| CHN2 | chr7 | ENST00000447171 | 29554384 | 29603286 | - |
| CUX1 | chr7 | RNZ31467 | 101502627 | 101502864 | + |
| DPY19L2P2 | chr7 | NR_003561 | 102815461 | 102920759 | - |
| FEZF1 | chr7 | NR_036484 | 121943711 | 121950131 | + |
| FKBP6 | chr7 | NR_026690 | 73149398 | 73150330 | + |
| GIMAP8 | chr7 | XLOC_006293 | 150130741 | 150145228 | + |
| HOXA10-HOXA9 | chr7 | ENST00000521197 | 27179982 | 27192316 | + |
| HOXA3 | chr7 | ENST00000522193 | 27153554 | 27162996 | + |
| HOXA4 | chr7 | ENST00000522193 | 27153554 | 27162996 | + |
| HOXA4 | chr7 | ENST00000521197 | 27179982 | 27192316 | + |
| HOXA5 | chr7 | ENST00000521197 | 27179982 | 27192316 | + |
| HOXA6 | chr7 | ENST00000521197 | 27179982 | 27192316 | + |
| HOXA7 | chr7 | ENST00000521197 | 27179982 | 27192316 | + |
| HOXA9 | chr7 | ENST00000521197 | 27179982 | 27192316 | + |
| LOC100093631 | chr7 | NR_026690 | 73149398 | 73150330 | + |
| LOC285972 | chr7 | XLOC_006293 | 150130741 | 150145228 | + |
| LOC645249 | chr7 | uc003wne.1 | 156803599 | 156809117 | + |
| LOC646329 | chr7 | ENST00000418546 | 130566520 | 130597956 | - |
| LOC646762 | chr7 | NR_036554 | 29725687 | 29782019 | + |
| MEOX2 | chr7 | ENST00000451240 | 15707571 | 15721605 | + |
| MEOX2 | chr7 | XLOC_005991 | 15728002 | 15736507 | + |
| MKLN1 | chr7 | uc003vqr.1 | 130994502 | 131012535 | - |
| MNX1 | chr7 | uc003wne.1 | 156803599 | 156809117 | + |
| MTERF | chr7 | XLOC_006168 | 91510142 | 91515201 | + |
| NCF1 | chr7 | NR_026690 | 73149398 | 73150330 | + |
| NPSR1 | chr7 | ENST00000539747 | 34390033 | 34911194 | - |
| PMS2L2 | chr7 | NR_026690 | 73149398 | 73150330 | + |
| PRR15 | chr7 | ENST00000447171 | 29554384 | 29603286 | - |
| SHFM1 | chr7 | XLOC_006529 | 96250838 | 96293650 | - |
| SNX10 | chr7 | EvoFold_20316 | 26411765 | 26412090 | + |
| SPDYE8P | chr7 | NR_026690 | 73149398 | 73150330 | + |
| TES | chr7 | uc003vhq.1 | 115893169 | 115895118 | + |
| ZNF107 | chr7 | uc003ttf.2 | 64141510 | 64147263 | + |
| ADAM3A | chr8 | NR_024106 | 39308563 | 39380470 | - |
| DEFB109P1B | chr8 | NR_027424 | 7812534 | 7866277 | + |
| DEFB130 | chr8 | NR_027425 | 11973290 | 12008698 | + |
| FAM66D | chr8 | NR_027425 | 11973290 | 12008698 | + |
| FAM66E | chr8 | NR_027424 | 7812534 | 7866277 | + |
| GGH | chr8 | XLOC_006819 | 63924579 | 63925933 | + |
| GRHL2 | chr8 | HIT000393196.5 | 102633031 | 102633399 | + |
| HAS2 | chr8 | ENST00000518865 | 122651532 | 122655691 | + |
| MYC | chr8 | XLOC_006925 | 128806778 | 129113503 | + |
| RIPK2 | chr8 | uc003yed.2 | 90623584 | 90769797 | - |
| RNF139 | chr8 | ENST00000519861 | 125485048 | 125486817 | - |
| SBSPON | chr8 | XLOC_006843 | 74005609 | 74006141 | + |
| TMEM75 | chr8 | XLOC_006925 | 128806778 | 129113503 | + |
| USP17L2 | chr8 | NR_027425 | 11973290 | 12008698 | + |
| USP17L3 | chr8 | NR_027424 | 7812534 | 7866277 | + |
| USP17L7 | chr8 | NR_027425 | 11973290 | 12008698 | + |
| USP17L8 | chr8 | NR_027424 | 7812534 | 7866277 | + |
| XKR6 | chr8 | uc010lrv.1 | 10980890 | 10982327 | - |
| ZFAT | chr8 | XLOC_006933 | 135732687 | 135736134 | + |
| ZFHX4 | chr8 | uc.243+ | 77690961 | 77691177 | + |
| ZFPM2 | chr8 | ASO3563 | 106808458 | 106810670 | - |
| ZNF705B | chr8 | NR_027424 | 7812534 | 7866277 | + |
| ZNF705D | chr8 | NR_027425 | 11973290 | 12008698 | + |
| ALDOB | chr9 | AK128504 | 104181298 | 104185086 | + |
| ANKRD20A3 | chr9 | NR_026558 | 42858151 | 42893137 | + |
| ANKRD20A3 | chr9 | NR_026759 | 43027747 | 43032877 | - |
| ANKRD20A3 | chr9 | XLOC_007365 | 45484571 | 45488153 | + |
| ANKRD20A3 | chr9 | uc004aej.2 | 66688087 | 66699951 | - |
| ANKRD20A3 | chr9 | uc004aen.1 | 67254266 | 67289492 | - |
| AQP7P3 | chr9 | NR_026558 | 42858151 | 42893137 | + |
| CBWD1 | chr9 | ENST00000416242 | 112715 | 113754 | - |
| CBWD5 | chr9 | NR_026558 | 42858151 | 42893137 | + |
| CBWD5 | chr9 | NR_026759 | 43027747 | 43032877 | - |
| CBWD5 | chr9 | XLOC_007365 | 45484571 | 45488153 | + |
| CBWD5 | chr9 | uc004aej.2 | 66688087 | 66699951 | - |
| CBWD5 | chr9 | uc004aen.1 | 67254266 | 67289492 | - |
| CNTNAP3B | chr9 | NR_026558 | 42858151 | 42893137 | + |
| CNTNAP3B | chr9 | NR_026759 | 43027747 | 43032877 | - |
| CRAT | chr9 | NR_028048 | 131857072 | 131873070 | - |
| DOLPP1 | chr9 | NR_028048 | 131857072 | 131873070 | - |
| FAM27E3 | chr9 | XLOC_007365 | 45484571 | 45488153 | + |
| FAM27E3 | chr9 | uc004aej.2 | 66688087 | 66699951 | - |
| FAM27E3 | chr9 | uc004aen.1 | 67254266 | 67289492 | - |
| FLJ35282 | chr9 | ENST00000448570 | 22767173 | 22768315 | + |
| FOXD4 | chr9 | ENST00000416242 | 112715 | 113754 | - |
| FOXD4L2 | chr9 | NR_026558 | 42858151 | 42893137 | + |
| FOXD4L2 | chr9 | NR_026759 | 43027747 | 43032877 | - |
| FOXD4L2 | chr9 | XLOC_007365 | 45484571 | 45488153 | + |
| FOXD4L2 | chr9 | uc004aej.2 | 66688087 | 66699951 | - |
| FOXD4L2 | chr9 | uc004aen.1 | 67254266 | 67289492 | - |
| FRRS1L | chr9 | AF038201 | 111892572 | 111893944 | - |
| HIATL2 | chr9 | uc004awr.1 | 99695548 | 99735230 | - |
| HRCT1 | chr9 | NR_024283 | 35909479 | 35911617 | + |
| IFNE | chr9 | NR_027054 | 21454266 | 21559697 | - |
| LOC100132167 | chr9 | XLOC_007365 | 45484571 | 45488153 | + |
| LOC100289019 | chr9 | XLOC_007858 | 130872812 | 130880972 | - |
| LOC286297 | chr9 | NR_026558 | 42858151 | 42893137 | + |
| LOC286297 | chr9 | NR_026759 | 43027747 | 43032877 | - |
| LOC286297 | chr9 | XLOC_007365 | 45484571 | 45488153 | + |
| LOC286297 | chr9 | uc004aej.2 | 66688087 | 66699951 | - |
| LOC389791 | chr9 | XLOC_007858 | 130872812 | 130880972 | - |
| LOC554249 | chr9 | NR_026558 | 42858151 | 42893137 | + |
| LOC554249 | chr9 | NR_026759 | 43027747 | 43032877 | - |
| LOC554249 | chr9 | XLOC_007365 | 45484571 | 45488153 | + |
| LOC643648 | chr9 | NR_026558 | 42858151 | 42893137 | + |
| LOC643648 | chr9 | NR_026759 | 43027747 | 43032877 | - |
| LOC643648 | chr9 | XLOC_007365 | 45484571 | 45488153 | + |
| NFIL3 | chr9 | XLOC_007456 | 94186603 | 94189429 | + |
| NUTM2G | chr9 | uc004awr.1 | 99695548 | 99735230 | - |
| PPP2R4 | chr9 | NR_028048 | 131857072 | 131873070 | - |
| PTGES2 | chr9 | XLOC_007858 | 130872812 | 130880972 | - |
| ROR2 | chr9 | RNZ34315 | 94658984 | 94659225 | + |
| SLC25A25 | chr9 | XLOC_007858 | 130872812 | 130880972 | - |
| SPATA31A5 | chr9 | NR_026558 | 42858151 | 42893137 | + |
| SPATA31A5 | chr9 | NR_026759 | 43027747 | 43032877 | - |
| SPATA31A5 | chr9 | XLOC_007365 | 45484571 | 45488153 | + |
| TMEM252 | chr9 | XLOC_007722 | 71158456 | 71161505 | - |
| ZNF189 | chr9 | AK128504 | 104181298 | 104185086 | + |
| ZNF322 | chr9 | uc004axc.1 | 99957632 | 99960147 | - |
| ZNF462 | chr9 | RNZ34485 | 109651288 | 109651566 | + |
| ADAM12 | chr10 | AK022196 | 127725869 | 127727892 | - |
| AGAP8 | chr10 | ENST00000430284 | 47279191 | 48998766 | - |
| AGAP8 | chr10 | XLOC_008814 | 48987629 | 49018413 | - |
| AGAP9 | chr10 | ENST00000430284 | 47279191 | 48998766 | - |
| AKR1C1 | chr10 | NR_026743 | 4913858 | 4958465 | - |
| ANXA8 | chr10 | ENST00000430284 | 47279191 | 48998766 | - |
| ANXA8L2 | chr10 | ENST00000430284 | 47279191 | 48998766 | - |
| C10orf11 | chr10 | EvoFold_29087 | 77831521 | 77831768 | - |
| DNA2 | chr10 | ENST00000439904 | 70237754 | 70240521 | - |
| FAM21B | chr10 | ENST00000430284 | 47279191 | 48998766 | - |
| FAM21B | chr10 | XLOC_008814 | 48987629 | 49018413 | - |
| FAM25C | chr10 | ENST00000430284 | 47279191 | 48998766 | - |
| FAM25C | chr10 | XLOC_008814 | 48987629 | 49018413 | - |
| FAM53B | chr10 | HIT000325925.9 | 126467359 | 126467666 | - |
| FRMPD2 | chr10 | ENST00000453853 | 46798369 | 46809021 | - |
| FRMPD2 | chr10 | ENST00000430284 | 47279191 | 48998766 | - |
| FRMPD2 | chr10 | XLOC_008814 | 48987629 | 49018413 | - |
| GDF10 | chr10 | ENST00000430284 | 47279191 | 48998766 | - |
| GDF2 | chr10 | ENST00000430284 | 47279191 | 48998766 | - |
| LOC100288974 | chr10 | ENST00000453174 | 81664653 | 81691557 | + |
| MBL2 | chr10 | ENST00000443523 | 54316742 | 54515169 | - |
| METTL10 | chr10 | HIT000325925.9 | 126467359 | 126467666 | - |
| OBFC1 | chr10 | XLOC_008597 | 105682667 | 105683570 | + |
| PTPN20A | chr10 | ENST00000453853 | 46798369 | 46809021 | - |
| PTPN20A | chr10 | ENST00000430284 | 47279191 | 48998766 | - |
| PTPN20B | chr10 | ENST00000453853 | 46798369 | 46809021 | - |
| PTPN20B | chr10 | ENST00000430284 | 47279191 | 48998766 | - |
| RAB11FIP2 | chr10 | ENST00000454781 | 119806514 | 119859650 | + |
| RBP3 | chr10 | ENST00000430284 | 47279191 | 48998766 | - |
| SFTPD | chr10 | ENST00000453174 | 81664653 | 81691557 | + |
| SLC25A16 | chr10 | ENST00000439904 | 70237754 | 70240521 | - |
| TAF3 | chr10 | RNZ4591 | 8026401 | 8026601 | + |
| TIAL1 | chr10 | uc.313+ | 121340173 | 121340404 | + |
| ZNF32 | chr10 | uc001jba.2 | 44124264 | 44170147 | + |
| ZNF488 | chr10 | ENST00000430284 | 47279191 | 48998766 | - |
| C11orf49 | chr11 | RNZ6858 | 47093391 | 47093793 | + |
| CADM1 | chr11 | RNZ7634 | 115050431 | 115050656 | + |
| CADM1 | chr11 | RNZ7649 | 115196263 | 115196928 | + |
| CADM1 | chr11 | ENST00000543182 | 115267473 | 115268376 | + |
| EHF | chr11 | RNZ6683 | 34653655 | 34653913 | + |
| GLYATL1 | chr11 | ASO3830 | 58720863 | 58722269 | - |
| KCNQ1 | chr11 | LIT1660 | 2668474 | 2668897 | + |
| KCNQ1 | chr11 | LIT1658 | 2696558 | 2696886 | + |
| KRTAP5-6 | chr11 | uc001ltz.1 | 1709526 | 1710286 | + |
| LOC100128239 | chr11 | ENST00000527712 | 133902699 | 133916744 | + |
| LOC646813 | chr11 | uc010rib.1 | 50375273 | 50379802 | + |
| MOB2 | chr11 | NR_026643 | 1686828 | 1689086 | + |
| MOB2 | chr11 | NR_026642 | 1704499 | 1706859 | - |
| MOB2 | chr11 | uc001ltz.1 | 1709526 | 1710286 | + |
| SERPING1 | chr11 | XLOC_009135 | 57386013 | 57388699 | + |
| SOX6 | chr11 | RNZ6455 | 16216184 | 16216426 | + |
| WT1 | chr11 | ENST00000395900 | 32457124 | 32462950 | + |
| ZBTB16 | chr11 | ASO3767 | 113931026 | 113932261 | - |
| CORO1C | chr12 | XLOC_009876 | 109029644 | 109036526 | + |
| DCP1B | chr12 | NR_036546 | 2038367 | 2045742 | - |
| DNAJC22 | chr12 | AL049983 | 49747460 | 49749819 | + |
| ENDOU | chr12 | ENST00000547799 | 48099867 | 48136077 | + |
| FIGNL2 | chr12 | ASO1937 | 52203488 | 52204384 | - |
| FZD10 | chr12 | ENST00000537095 | 130640278 | 130646801 | - |
| GPR182 | chr12 | uc010sqx.1 | 57348915 | 57397270 | - |
| HOXC4 | chr12 | NR_026655 | 54452037 | 54516018 | + |
| INHBC | chr12 | ENST00000547552 | 57824898 | 57827718 | + |
| IQSEC3 | chr12 | ENST00000537961 | 276167 | 291565 | - |
| KLRC4 | chr12 | ENST00000500682 | 10516367 | 10551105 | + |
| KLRC4-KLRK1 | chr12 | ENST00000500682 | 10516367 | 10551105 | + |
| KLRK1 | chr12 | ENST00000500682 | 10516367 | 10551105 | + |
| LRCOL1 | chr12 | ENST00000538369 | 133179735 | 133186983 | - |
| LTA4H | chr12 | uc009ztj.2 | 96405073 | 96408952 | - |
| MAGOHB | chr12 | uc009zhn.2 | 10741077 | 10752434 | - |
| P2RX2 | chr12 | ENST00000538369 | 133179735 | 133186983 | - |
| PAH | chr12 | RNZ9526 | 103283819 | 103284098 | + |
| R3HDM2 | chr12 | ENST00000547552 | 57824898 | 57827718 | + |
| RAPGEF3 | chr12 | ENST00000547799 | 48099867 | 48136077 | + |
| RDH16 | chr12 | uc010sqx.1 | 57348915 | 57397270 | - |
| RPAP3 | chr12 | ENST00000547799 | 48099867 | 48136077 | + |
| SCN8A | chr12 | ASO1937 | 52203488 | 52204384 | - |
| SELPLG | chr12 | XLOC_009876 | 109029644 | 109036526 | + |
| SLC6A12 | chr12 | ENST00000537961 | 276167 | 291565 | - |
| SYT1 | chr12 | ENST00000550268 | 79786696 | 79849240 | - |
| TAC3 | chr12 | uc010sqx.1 | 57348915 | 57397270 | - |
| TMEM132D | chr12 | uc001uic.2 | 129594241 | 129597842 | + |
| WNT5B | chr12 | XLOC_009974 | 1762423 | 1772556 | - |
| ZBTB39 | chr12 | uc010sqx.1 | 57348915 | 57397270 | - |
| ZNF705A | chr12 | uc009zgc.2 | 8332804 | 8356981 | + |
| CPB2 | chr13 | uc001vau.1 | 46626982 | 46675482 | + |
| F10 | chr13 | LIT3592 | 113813440 | 113814140 | - |
| HTR2A | chr13 | XLOC_010384 | 47402275 | 47407401 | + |
| KLHL1 | chr13 | XLOC_010422 | 70689281 | 70705153 | + |
| LHFP | chr13 | uc001uxg.2 | 40100015 | 40107556 | - |
| MBNL2 | chr13 | ENST00000453862 | 97824722 | 97831876 | - |
| MTRF1 | chr13 | uc001uyb.2 | 41793281 | 41794134 | + |
| NBEA | chr13 | EvoFold_34741 | 36089039 | 36089304 | + |
| PDS5B | chr13 | HIT000220172.7 | 33242538 | 33242992 | - |
| PROZ | chr13 | LIT3592 | 113813440 | 113814140 | - |
| PRR20D | chr13 | XLOC_010629 | 57711062 | 57711858 | - |
| SERP2 | chr13 | ENST00000432701 | 44974486 | 44980002 | - |
| SLC7A1 | chr13 | RNZ9831 | 30160970 | 30161289 | + |
| SLITRK5 | chr13 | ENST00000453832 | 88267817 | 88323361 | - |
| TPT1 | chr13 | ENST00000420693 | 45924457 | 45925485 | + |
| ZC3H13 | chr13 | uc001vau.1 | 46626982 | 46675482 | + |
| C14orf25 | chr14 | XLOC_010807 | 38205180 | 38208450 | + |
| EGLN3 | chr14 | RNZ11047 | 34412366 | 34412620 | + |
| GPHN | chr14 | uc001xiv.3 | 66953108 | 66965271 | + |
| GPR65 | chr14 | uc001xvw.2 | 88490893 | 88553681 | + |
| PRKD1 | chr14 | RNZ10900 | 30302933 | 30303152 | + |
| RAD51B | chr14 | RNZ11456 | 68715421 | 68715640 | + |
| SAMD4A | chr14 | RNZ11273 | 55179015 | 55179226 | + |
| SERPINA4 | chr14 | XLOC_011107 | 95024729 | 95027008 | - |
| SMOC1 | chr14 | RNZ11522 | 70348957 | 70349237 | + |
| SMOC1 | chr14 | LIT3591 | 70419070 | 70420113 | + |
| SMOC1 | chr14 | RNZ11526 | 70444055 | 70444292 | + |
| SMOC1 | chr14 | RNZ11529 | 70485578 | 70485837 | + |
| ALDH1A2 | chr15 | XLOC_011486 | 58572414 | 58576475 | - |
| BCL2L10 | chr15 | XLOC_011480 | 52407273 | 52409155 | - |
| CSPG4P8 | chr15 | NR_033579 | 82970478 | 83145983 | + |
| FAM63B | chr15 | XLOC_011488 | 59060272 | 59063173 | - |
| GNB5 | chr15 | XLOC_011480 | 52407273 | 52409155 | - |
| GNB5 | chr15 | AF007131 | 52472413 | 52498071 | + |
| GOLGA6L1 | chr15 | XLOC_011171 | 24356506 | 24358424 | + |
| GOLGA6L1 | chr15 | XLOC_011401 | 24405063 | 24630598 | - |
| GOLGA6L1 | chr15 | XLOC_011172 | 24415174 | 24426091 | + |
| GOLGA6L1 | chr15 | XLOC_011175 | 24532701 | 24603338 | + |
| GOLGA6L1 | chr15 | NR_022009 | 25380788 | 25383200 | + |
| GOLGA6L1 | chr15 | AF400501 | 25463498 | 25620623 | + |
| GOLGA6L10 | chr15 | NR_033579 | 82970478 | 83145983 | + |
| GOLGA6L9 | chr15 | NR_033579 | 82970478 | 83145983 | + |
| LOC100129973 | chr15 | AF007131 | 52472413 | 52498071 | + |
| LOC440295 | chr15 | NR_033579 | 82970478 | 83145983 | + |
| LOC727751 | chr15 | NR_033579 | 82970478 | 83145983 | + |
| LOC80154 | chr15 | NR_033579 | 82970478 | 83145983 | + |
| MTHFS | chr15 | uc002bey.2 | 80137383 | 80139529 | - |
| MYO5C | chr15 | AF007131 | 52472413 | 52498071 | + |
| PCSK6 | chr15 | XLOC_011390 | 101835622 | 101838894 | + |
| PML | chr15 | XLOC_011309 | 74346639 | 74348558 | + |
| PPIP5K1 | chr15 | RNZ12413 | 43851597 | 43851842 | + |
| RORA | chr15 | RNZ12639 | 61330498 | 61330698 | + |
| RORA | chr15 | RNZ12646 | 61413972 | 61414323 | + |
| RPS17L | chr15 | NR_033579 | 82970478 | 83145983 | + |
| SMAD3 | chr15 | RNZ12774 | 67367371 | 67367588 | + |
| SNRPA1 | chr15 | XLOC_011390 | 101835622 | 101838894 | + |
| SNURF-SNRPN | chr15 | NR_022009 | 25380788 | 25383200 | + |
| SNURF-SNRPN | chr15 | AF400501 | 25463498 | 25620623 | + |
| ST20-MTHFS | chr15 | uc002bey.2 | 80137383 | 80139529 | - |
| UBE3A | chr15 | AF400501 | 25463498 | 25620623 | + |
| SEPT12 | chr16 | BC148245 | 4845703 | 4846312 | + |
| ABCC6P1 | chr16 | NR_003569 | 18582569 | 18609610 | + |
| CA7 | chr16 | RNZ14285 | 66879781 | 66880058 | + |
| CRNDE | chr16 | XLOC_011950 | 54951850 | 54963045 | - |
| GLYR1 | chr16 | BC148245 | 4845703 | 4846312 | + |
| IRX5 | chr16 | XLOC_011950 | 54951850 | 54963045 | - |
| LMF1 | chr16 | NR_036442 | 1025760 | 1031318 | - |
| LOC100132247 | chr16 | uc010vdn.1 | 29086162 | 29128036 | + |
| LOC440335 | chr16 | BC148245 | 4845703 | 4846312 | + |
| MT1A | chr16 | NR_001447 | 56651372 | 56652730 | + |
| MT1A | chr16 | uc002ejp.1 | 56669674 | 56670998 | + |
| MT1A | chr16 | NR_027781 | 56677598 | 56678853 | + |
| MT1B | chr16 | NR_027781 | 56677598 | 56678853 | + |
| MT1DP | chr16 | uc002ejp.1 | 56669674 | 56670998 | + |
| MT1DP | chr16 | NR_027781 | 56677598 | 56678853 | + |
| MT1E | chr16 | NR_001447 | 56651372 | 56652730 | + |
| MT1E | chr16 | uc002ejp.1 | 56669674 | 56670998 | + |
| MT1G | chr16 | uc010vhg.1 | 56710043 | 56711675 | + |
| MT1H | chr16 | uc010vhg.1 | 56710043 | 56711675 | + |
| MT1IP | chr16 | uc010vhg.1 | 56710043 | 56711675 | + |
| MT1JP | chr16 | uc002ejp.1 | 56669674 | 56670998 | + |
| MT1JP | chr16 | NR_027781 | 56677598 | 56678853 | + |
| MT1L | chr16 | NR_001447 | 56651372 | 56652730 | + |
| MT1M | chr16 | NR_001447 | 56651372 | 56652730 | + |
| MT1M | chr16 | uc002ejp.1 | 56669674 | 56670998 | + |
| MT1M | chr16 | NR_027781 | 56677598 | 56678853 | + |
| MT1X | chr16 | uc010vhg.1 | 56710043 | 56711675 | + |
| MT2A | chr16 | NR_001447 | 56651372 | 56652730 | + |
| NOMO2 | chr16 | NR_003569 | 18582569 | 18609610 | + |
| NPIPL1 | chr16 | uc010vdn.1 | 29086162 | 29128036 | + |
| NPIPL3 | chr16 | uc010vdn.1 | 29086162 | 29128036 | + |
| ROGDI | chr16 | BC148245 | 4845703 | 4846312 | + |
| RRN3P2 | chr16 | uc010vdn.1 | 29086162 | 29128036 | + |
| SOX8 | chr16 | NR_036442 | 1025760 | 1031318 | - |
| SRL | chr16 | XLOC_011866 | 4230068 | 4233668 | - |
| AOC3 | chr17 | NR_002773 | 41019161 | 41021234 | + |
| ARL17A | chr17 | NONE | 43990941 | 43991991 | - |
| ARL17A | chr17 | NONE | 43994767 | 43994987 | - |
| ARL17A | chr17 | NONE | 43998757 | 43999237 | - |
| ARL17A | chr17 | NONE | 44000827 | 44001877 | - |
| CYB5D1 | chr17 | uc010cno.1 | 7771138 | 7777031 | - |
| DCXR | chr17 | XLOC_012343 | 79995796 | 79997235 | + |
| EFCAB13 | chr17 | NR_027416 | 45500842 | 45504058 | + |
| FAM117A | chr17 | RNZ15658 | 47817041 | 47817440 | + |
| HOXB6 | chr17 | ENST00000438772 | 46684988 | 46716647 | - |
| HOXB7 | chr17 | ENST00000438772 | 46684988 | 46716647 | - |
| HOXB8 | chr17 | ENST00000438772 | 46684988 | 46716647 | - |
| HOXB9 | chr17 | ENST00000438772 | 46684988 | 46716647 | - |
| HOXB9 | chr17 | ENST00000433510 | 46713653 | 46724385 | - |
| LOC100506388 | chr17 | uc010cjm.1 | 181048 | 183234 | + |
| LRRC45 | chr17 | XLOC_012343 | 79995796 | 79997235 | + |
| LSMD1 | chr17 | uc010cno.1 | 7771138 | 7777031 | - |
| LUC7L3 | chr17 | NR_024626 | 48834758 | 48844918 | - |
| MAPT | chr17 | NONE | 43990941 | 43991991 | - |
| MAPT | chr17 | NONE | 43994767 | 43994987 | - |
| MAPT | chr17 | NONE | 43998757 | 43999237 | - |
| MAPT | chr17 | NONE | 44000827 | 44001877 | - |
| MSI2 | chr17 | AK129642 | 55731047 | 55732651 |  |
| PLEKHM1P | chr17 | NR_026899 | 62745779 | 62778117 | - |
| RAC3 | chr17 | XLOC_012343 | 79995796 | 79997235 | + |
| RFNG | chr17 | XLOC_012343 | 79995796 | 79997235 | + |
| RPH3AL | chr17 | uc010cjm.1 | 181048 | 183234 | + |
| SOX9 | chr17 | ENST00000529667 | 70067182 | 70115634 | - |
| TLK2 | chr17 | RNZ15921 | 60556818 | 60557179 | + |
| ADNP2 | chr18 | NR_028340 | 77905806 | 77936315 | + |
| CCDC68 | chr18 | XLOC_012847 | 52558107 | 52565201 | - |
| DLGAP1 | chr18 | uc002kmi.2 | 3603735 | 3608319 | + |
| LOC339290 | chr18 | NR_026849 | 5236722 | 5238028 | - |
| PARD6G | chr18 | NR_028340 | 77905806 | 77936315 | + |
| RAB27B | chr18 | XLOC_012847 | 52558107 | 52565201 | - |
| SETBP1 | chr18 | EvoFold_43935 | 42518260 | 42518461 | + |
| SLC25A52 | chr18 | BC036040 | 29304160 | 29434944 | + |
| TRAPPC8 | chr18 | BC036040 | 29304160 | 29434944 | + |
| ZNF407 | chr18 | uc.442+ | 72592711 | 72592960 | + |
| ZNF521 | chr18 | uc.424+ | 22767778 | 22767993 | + |
| APOC1 | chr19 | NR_028412 | 45430059 | 45434281 | + |
| CYP2A7 | chr19 | NR_001278 | 41430169 | 41456565 | + |
| CYP2B7P1 | chr19 | NR_001278 | 41430169 | 41456565 | + |
| FXYD5 | chr19 | uc002nyj.1 | 35657757 | 35660784 | + |
| LOC100134317 | chr19 | XLOC_013054 | 36800485 | 36812260 | + |
| PSG1 | chr19 | NR_026824 | 43341148 | 43359870 | - |
| PSG3 | chr19 | NR_026824 | 43341148 | 43359870 | - |
| SAMD4B | chr19 | uc002olc.1 | 39840439 | 39843129 | + |
| TNFSF14 | chr19 | XLOC_012931 | 6661465 | 6662832 | + |
| ADA | chr20 | uc002xmm.1 | 43289247 | 43367608 | - |
| ERGIC3 | chr20 | NR_024377 | 34146506 | 34195484 | - |
| FER1L4 | chr20 | NR_024377 | 34146506 | 34195484 | - |
| FOXA2 | chr20 | ENST00000450346 | 22541191 | 22559280 |  |
| HM13 | chr20 | NR_003677 | 30135184 | 30136019 | + |
| HNF4A | chr20 | ASO3676 | 43000767 | 43031509 | - |
| KCNK15 | chr20 | uc002xmm.1 | 43289247 | 43367608 | - |
| LOC100127888 | chr20 | NR_024470 | 61294378 | 61297973 | - |
| LOC284801 | chr20 | ENST00000416638 | 26171624 | 26174582 | + |
| MMP9 | chr20 | ENST00000419897 | 44649257 | 44650366 | - |
| SLC12A5 | chr20 | ENST00000419897 | 44649257 | 44650366 | - |
| SLCO4A1 | chr20 | NR_024470 | 61294378 | 61297973 | - |
| SPAG4 | chr20 | NR_024377 | 34146506 | 34195484 | - |
| WISP2 | chr20 | uc002xmm.1 | 43289247 | 43367608 | - |
| BTG3 | chr21 | AL049332 | 18963578 | 18967633 | + |
| CXADR | chr21 | AL049332 | 18963578 | 18967633 | + |
| LOC100133286 | chr21 | XLOC_013931 | 37477178 | 37481988 | + |
| NCAM2 | chr21 | HIT000393201.3 | 22859409 | 22859700 | + |
| RIPK4 | chr21 | XLOC_013950 | 43194020 | 43196304 | + |
| RUNX1 | chr21 | ASO3481 | 36314113 | 36317337 | - |
| RUNX1 | chr21 | NR_026812 | 36410232 | 36411723 | - |
| SOD1 | chr21 | uc002yoz.1 | 33026870 | 33031813 | - |
| TSPEAR | chr21 | ENST00000449713 | 45905459 | 45910171 | + |
| C22orf26 | chr22 | NR_027034 | 46449725 | 46454402 | + |
| DGCR2 | chr22 | ENST00000438934 | 18958026 | 19018742 | + |
| LOC554174 | chr22 | NR_027034 | 46449725 | 46454402 | + |
| MKL1 | chr22 | AB051446 | 40876291 | 40883142 | - |
| MYH9 | chr22 | uc003apj.1 | 36730923 | 36732334 | - |
| PPARA | chr22 | XLOC_014268 | 46533091 | 46539488 | + |
| SEPT6 | chrX | ASO3646 | 118827530 | 118830022 | + |
| ABCB7 | chrX | ASO2242 | 74292761 | 74297818 | + |
| ACSL4 | chrX | HIT000245014.7 | 108975212 | 108975708 | - |
| DIAPH2 | chrX | L22650 | 96788171 | 96872312 | - |
| PAK3 | chrX | AF070581 | 110368941 | 110370682 | + |
