# Supplementary material for: Global identification and characterization of lncRNAs that control inflammation in malignant cholangiocytes
Source: BMC Genomics. 2018 Oct 11;19:735. doi: 10.1186/s12864-018-5133-8 (PMC6180422; doi:10.1186/s12864-018-5133-8)
Supplement: Supplementary file 6 — Table S4. GO clusters of adjacent genes of dysregulated lncRNAs. (DOCX 33 kb) [file 12864_2018_5133_MOESM6_ESM.docx]

**Table S4. GO clusters of adjacent genes of dysregulated lncRNAs.**

| GO_Term | Name | P-Value | FDR |
| --- | --- | --- | --- |
| GO0009952 | anterior/posterior pattern formation | 1.00E-08 | 1.74E-05 |
| GO0003002 | regionalization | 1.13E-08 | 1.97E-05 |
| GO0043009 | chordate embryonic development | 1.63E-08 | 2.84E-05 |
| GO0009792 | embryonic development ending in birth or egg hatching | 1.96E-08 | 3.40E-05 |
| GO0001501 | skeletal system development | 3.22E-08 | 5.60E-05 |
| GO0007389 | pattern specification process | 9.33E-08 | 1.62E-04 |
| GO0048706 | embryonic skeletal system development | 9.59E-08 | 1.67E-04 |
| GO0048568 | embryonic organ development | 2.15E-07 | 3.74E-04 |
| GO0048598 | embryonic morphogenesis | 1.03E-06 | 0.001792 |
| GO0048705 | skeletal system morphogenesis | 5.85E-06 | 0.010148 |
| GO0048562 | embryonic organ morphogenesis | 6.66E-06 | 0.011561 |
| GO0048704 | embryonic skeletal system morphogenesis | 3.04E-05 | 0.052737 |
| GO0006069 | ethanol oxidation | 9.94E-05 | 0.172393 |
| GO0006067 | ethanol metabolic process | 9.94E-05 | 0.172393 |
| GO0034308 | monohydric alcohol metabolic process | 9.94E-05 | 0.172393 |
| GO0006350 | transcription | 2.30E-04 | 0.398104 |
| GO0030324 | lung development | 3.07E-04 | 0.531388 |
| GO0050817 | coagulation | 3.84E-04 | 0.663853 |
| GO0007596 | blood coagulation | 3.84E-04 | 0.663853 |
| GO0030323 | respiratory tube development | 3.84E-04 | 0.663853 |
| GO0051173 | positive regulation of nitrogen compound metabolic process | 4.01E-04 | 0.694375 |
| GO0031328 | positive regulation of cellular biosynthetic process | 4.88E-04 | 0.844668 |
| GO0045944 | positive regulation of transcription from RNA polymerase II promoter | 5.09E-04 | 0.88015 |
| GO0010557 | positive regulation of macromolecule biosynthetic process | 5.18E-04 | 0.896163 |
| GO0045935 | positive regulation of nucleobase, nucleoside, nucleotide and nucleic acid metabolic process | 5.50E-04 | 0.950281 |
| GO0007599 | hemostasis | 5.85E-04 | 1.010339 |
| GO0060541 | respiratory system development | 5.85E-04 | 1.010339 |
| GO0048534 | hemopoietic or lymphoid organ development | 5.95E-04 | 1.027294 |
| GO0009891 | positive regulation of biosynthetic process | 6.16E-04 | 1.064712 |
| GO0045941 | positive regulation of transcription | 6.26E-04 | 1.081411 |
| GO0045893 | positive regulation of transcription, DNA-dependent | 7.80E-04 | 1.345584 |
| GO0051254 | positive regulation of RNA metabolic process | 8.83E-04 | 1.522483 |
| GO0010628 | positive regulation of gene expression | 9.50E-04 | 1.637076 |
| GO0030099 | myeloid cell differentiation | 9.52E-04 | 1.640171 |
| GO0042060 | wound healing | 0.001001 | 1.724039 |
| GO0010604 | positive regulation of macromolecule metabolic process | 0.00104 | 1.790947 |
| GO0002520 | immune system development | 0.001094 | 1.881745 |
| GO0006357 | regulation of transcription from RNA polymerase II promoter | 0.001234 | 2.120274 |
| GO0030097 | hemopoiesis | 0.002053 | 3.50575 |
| GO0045449 | regulation of transcription | 0.003274 | 5.534368 |
| GO0051186 | cofactor metabolic process | 0.003707 | 6.245422 |
| GO0050878 | regulation of body fluid levels | 0.003755 | 6.322595 |
| GO0035270 | endocrine system development | 0.003875 | 6.519006 |
| GO0009880 | embryonic pattern specification | 0.004924 | 8.213954 |
| GO0051604 | protein maturation | 0.005244 | 8.724282 |
| GO0051216 | cartilage development | 0.005472 | 9.087059 |
| GO0009611 | response to wounding | 0.006078 | 10.04366 |
| GO0006732 | coenzyme metabolic process | 0.006385 | 10.5259 |
| GO0006355 | regulation of transcription, DNA-dependent | 0.006404 | 10.55475 |
| GO0051252 | regulation of RNA metabolic process | 0.006413 | 10.56927 |
| GO0042127 | regulation of cell proliferation | 0.007378 | 12.06462 |
| GO0019748 | secondary metabolic process | 0.007504 | 12.25895 |
| GO0051241 | negative regulation of multicellular organismal process | 0.009871 | 15.82268 |
| GO0051605 | protein maturation by peptide bond cleavage | 0.011194 | 17.75419 |
| GO0016485 | protein processing | 0.011304 | 17.91228 |
| GO0045930 | negative regulation of mitotic cell cycle | 0.011629 | 18.37984 |
| GO0035136 | forelimb morphogenesis | 0.011629 | 18.37984 |
| GO0055114 | oxidation reduction | 0.012841 | 20.09978 |
| GO0000122 | negative regulation of transcription from RNA polymerase II promoter | 0.014027 | 21.75035 |
| GO0051336 | regulation of hydrolase activity | 0.016157 | 24.63456 |
| GO0021675 | nerve development | 0.016556 | 25.16275 |
| GO0001822 | kidney development | 0.018461 | 27.63998 |
| GO0032582 | negative regulation of gene-specific transcription | 0.020328 | 29.99304 |
| GO0035295 | tube development | 0.022874 | 33.086 |
| GO0021602 | cranial nerve morphogenesis | 0.02289 | 33.10529 |
| GO0010038 | response to metal ion | 0.022903 | 33.12095 |
| GO0042592 | homeostatic process | 0.023283 | 33.57014 |
| GO0045892 | negative regulation of transcription, DNA-dependent | 0.02447 | 34.95819 |
| GO0048736 | appendage development | 0.025138 | 35.72721 |
| GO0060173 | limb development | 0.025138 | 35.72721 |
| GO0046620 | regulation of organ growth | 0.026992 | 37.81674 |
| GO0030878 | thyroid gland development | 0.027078 | 37.91241 |
| GO0060420 | regulation of heart growth | 0.027078 | 37.91241 |
| GO0046688 | response to copper ion | 0.027078 | 37.91241 |
| GO0030278 | regulation of ossification | 0.027659 | 38.55287 |
| GO0051253 | negative regulation of RNA metabolic process | 0.027771 | 38.67592 |
| GO0001889 | liver development | 0.028131 | 39.06863 |
| GO0048732 | gland development | 0.028462 | 39.42797 |
| GO0006879 | cellular iron ion homeostasis | 0.029414 | 40.45058 |
| GO0006919 | activation of caspase activity | 0.029877 | 40.94207 |
| GO0010605 | negative regulation of macromolecule metabolic process | 0.030176 | 41.25736 |
| GO0001655 | urogenital system development | 0.03326 | 44.41717 |
| GO0043603 | cellular amide metabolic process | 0.033559 | 44.71494 |
| GO0010565 | regulation of cellular ketone metabolic process | 0.035494 | 46.60622 |
| GO0010035 | response to inorganic substance | 0.036061 | 47.14771 |
| GO0043280 | positive regulation of caspase activity | 0.039556 | 50.37833 |
| GO0010952 | positive regulation of peptidase activity | 0.039556 | 50.37833 |
| GO0031016 | pancreas development | 0.040207 | 50.9593 |
| GO0051055 | negative regulation of lipid biosynthetic process | 0.041275 | 51.8981 |
| GO0035113 | embryonic appendage morphogenesis | 0.04144 | 52.04118 |
| GO0030326 | embryonic limb morphogenesis | 0.04144 | 52.04118 |
| GO0051345 | positive regulation of hydrolase activity | 0.042648 | 53.08009 |
| GO0055072 | iron ion homeostasis | 0.043177 | 53.52851 |
| GO0007548 | sex differentiation | 0.047551 | 57.08215 |
| GO0042692 | muscle cell differentiation | 0.049151 | 58.31659 |
| GO0010629 | negative regulation of gene expression | 0.050108 | 59.03918 |
| GO0045471 | response to ethanol | 0.050821 | 59.56954 |
| GO0010043 | response to zinc ion | 0.051965 | 60.40774 |
| GO0010551 | regulation of specific transcription from RNA polymerase II promoter | 0.054585 | 62.26473 |
| GO0006769 | nicotinamide metabolic process | 0.056111 | 63.30877 |
| GO0046496 | nicotinamide nucleotide metabolic process | 0.056111 | 63.30877 |
| GO0030182 | neuron differentiation | 0.057038 | 63.92904 |
| GO0019438 | aromatic compound biosynthetic process | 0.05764 | 64.32683 |
| GO0010921 | regulation of phosphatase activity | 0.05764 | 64.32683 |
| GO0060627 | regulation of vesicle-mediated transport | 0.058736 | 65.04061 |
| GO0051301 | cell division | 0.059062 | 65.25017 |
| GO0010553 | negative regulation of specific transcription from RNA polymerase II promoter | 0.059601 | 65.59434 |
| GO0009820 | alkaloid metabolic process | 0.059601 | 65.59434 |
| GO0021915 | neural tube development | 0.060968 | 66.45216 |
| GO0019362 | pyridine nucleotide metabolic process | 0.063191 | 67.8047 |
| GO0001649 | osteoblast differentiation | 0.063191 | 67.8047 |
| GO0042594 | response to starvation | 0.063191 | 67.8047 |
| GO0021545 | cranial nerve development | 0.063518 | 67.99915 |
| GO0035115 | embryonic forelimb morphogenesis | 0.063518 | 67.99915 |
| GO0009108 | coenzyme biosynthetic process | 0.06366 | 68.08361 |
| GO0007423 | sensory organ development | 0.063961 | 68.2611 |
| GO0001823 | mesonephros development | 0.064008 | 68.289 |
| GO0070050 | neuron maintenance | 0.064008 | 68.289 |
| GO0021615 | glossopharyngeal nerve morphogenesis | 0.064008 | 68.289 |
| GO0021563 | glossopharyngeal nerve development | 0.064008 | 68.289 |
| GO0035108 | limb morphogenesis | 0.065293 | 69.03604 |
| GO0035107 | appendage morphogenesis | 0.065293 | 69.03604 |
| GO0002541 | activation of plasma proteins involved in acute inflammatory response | 0.066878 | 69.93553 |
| GO0031667 | response to nutrient levels | 0.067113 | 70.06675 |
| GO0048872 | homeostasis of number of cells | 0.067566 | 70.31771 |
| GO0008284 | positive regulation of cell proliferation | 0.069182 | 71.19841 |
| GO0006805 | xenobiotic metabolic process | 0.069588 | 71.41563 |
| GO0032012 | regulation of ARF protein signal transduction | 0.070662 | 71.9833 |
| GO0032583 | regulation of gene-specific transcription | 0.07309 | 73.22734 |
| GO0046890 | regulation of lipid biosynthetic process | 0.07454 | 73.94526 |
| GO0007178 | transmembrane receptor protein serine/threonine kinase signaling pathway | 0.074645 | 73.99691 |
| GO0046661 | male sex differentiation | 0.07504 | 74.18895 |
| GO0051051 | negative regulation of transport | 0.075165 | 74.24944 |
| GO0045923 | positive regulation of fatty acid metabolic process | 0.075838 | 74.57277 |
| GO0030334 | regulation of cell migration | 0.077025 | 75.13362 |
| GO0009890 | negative regulation of biosynthetic process | 0.077401 | 75.30896 |
| GO0016481 | negative regulation of transcription | 0.078141 | 75.65052 |
| GO0045834 | positive regulation of lipid metabolic process | 0.07851 | 75.81938 |
| GO0042445 | hormone metabolic process | 0.082113 | 77.40944 |
| GO0048538 | thymus development | 0.082259 | 77.4718 |
| GO0048638 | regulation of developmental growth | 0.082571 | 77.60434 |
| GO0051094 | positive regulation of developmental process | 0.084727 | 78.50075 |
| GO0051346 | negative regulation of hydrolase activity | 0.086721 | 79.2995 |
| GO0070482 | response to oxygen levels | 0.088317 | 79.91867 |
| GO0009954 | proximal/distal pattern formation | 0.08884 | 80.1179 |
| GO0043271 | negative regulation of ion transport | 0.08884 | 80.1179 |
| GO0001523 | retinoid metabolic process | 0.08884 | 80.1179 |
| GO0016101 | diterpenoid metabolic process | 0.08884 | 80.1179 |
| GO0042558 | pteridine and derivative metabolic process | 0.08884 | 80.1179 |
| GO0009410 | response to xenobiotic stimulus | 0.08884 | 80.1179 |
| GO0048806 | genitalia development | 0.08884 | 80.1179 |
| GO0019217 | regulation of fatty acid metabolic process | 0.090957 | 80.90482 |
| GO0043281 | regulation of caspase activity | 0.093886 | 81.94541 |
| GO0048878 | chemical homeostasis | 0.095018 | 82.3332 |
| GO0050778 | positive regulation of immune response | 0.097739 | 83.23317 |
| GO0019216 | regulation of lipid metabolic process | 0.098187 | 83.37686 |
| GO0055088 | lipid homeostasis | 0.099681 | 83.84865 |
| GO0043392 | negative regulation of DNA binding | 0.099681 | 83.84865 |
