# Supplementary material for: Global identification and characterization of lncRNAs that control inflammation in malignant cholangiocytes
Source: BMC Genomics. 2018 Oct 11;19:735. doi: 10.1186/s12864-018-5133-8 (PMC6180422; doi:10.1186/s12864-018-5133-8)
Supplement: Supplementary file 7 — Table S5. Dysregulated lncRNAs adjacent to inflammatory genes. (DOCX 50 kb) [file 12864_2018_5133_MOESM7_ESM.docx]

**Table S5. Dysregulated lncRNAs adjacent to inflammatory genes.**

| Gene_symbol | Chromosome | lncRNA_symbol | lncRNA_start | lncRNA_end | lncRNA_strand |
| --- | --- | --- | --- | --- | --- |
| PARK7 | chr1 | ENST00000423628 | 8066073 | 8066784 | - |
| CTNNBIP1 | chr1 | ENST00000441033 | 9561150 | 9563530 | - |
| PIK3CD | chr1 | ENST00000441033 | 9561150 | 9563530 | - |
| TNFRSF1B | chr1 | XLOC_000055 | 12587485 | 12588702 | + |
| C8A | chr1 | XLOC_000845 | 56797823 | 56840926 | - |
| C8B | chr1 | XLOC_000845 | 56797823 | 56840926 | - |
| C8A | chr1 | ENST00000451914 | 56880634 | 56881638 | + |
| C8B | chr1 | ENST00000451914 | 56880634 | 56881638 | + |
| C8A | chr1 | RNZ2250 | 57354732 | 57354932 | + |
| C8B | chr1 | RNZ2250 | 57354732 | 57354932 | + |
| IL23R | chr1 | XLOC_000236 | 67987995 | 67998015 | + |
| IL23R | chr1 | ENST00000434072 | 68604039 | 68607471 | + |
| F3 | chr1 | ASO3817 | 94057634 | 94068376 | + |
| F3 | chr1 | XLOC_000302 | 95007607 | 95008571 | + |
| F3 | chr1 | uc001dqu.2 | 95123089 | 95285834 | - |
| NOTCH2 | chr1 | XLOC_000984 | 119860047 | 119860947 | - |
| NOTCH2 | chr1 | RNZ3190 | 119896638 | 119896872 | + |
| S100A12 | chr1 | AF005081 | 152692385 | 152692799 | + |
| S100A8 | chr1 | AF005081 | 152692385 | 152692799 | + |
| S100A9 | chr1 | AF005081 | 152692385 | 152692799 | + |
| GBA | chr1 | ENST00000454974 | 155966113 | 155972355 | - |
| AIM2 | chr1 | ENST00000423943 | 159931007 | 159948851 | + |
| APCS | chr1 | ENST00000423943 | 159931007 | 159948851 | + |
| CRP | chr1 | ENST00000423943 | 159931007 | 159948851 | + |
| DARC | chr1 | ENST00000423943 | 159931007 | 159948851 | + |
| FCER1A | chr1 | ENST00000423943 | 159931007 | 159948851 | + |
| IFI16 | chr1 | ENST00000423943 | 159931007 | 159948851 | + |
| KCNJ10 | chr1 | ENST00000423943 | 159931007 | 159948851 | + |
| SERPINC1 | chr1 | ENST00000424181 | 173991646 | 173992123 | + |
| TNFSF4 | chr1 | ENST00000424181 | 173991646 | 173992123 | + |
| DUSP10 | chr1 | ENST00000455363 | 222626081 | 222628047 | - |
| TLR5 | chr1 | ENST00000455363 | 222626081 | 222628047 | - |
| TLR5 | chr1 | ENST00000439440 | 222988452 | 223000924 | + |
| SDC1 | chr2 | XLOC_002012 | 21316934 | 21319427 | - |
| SDC1 | chr2 | XLOC_002013 | 21320550 | 21322019 | - |
| NLRC4 | chr2 | ENST00000405650 | 31747549 | 31806136 | - |
| REG3A | chr2 | ENST00000420648 | 78826187 | 78826532 | - |
| REG3G | chr2 | ENST00000420648 | 78826187 | 78826532 | - |
| IL1A | chr2 | ENST00000446595 | 114286223 | 114300235 | - |
| IL1B | chr2 | ENST00000446595 | 114286223 | 114300235 | - |
| IL1RN | chr2 | ENST00000446595 | 114286223 | 114300235 | - |
| IL36A | chr2 | ENST00000446595 | 114286223 | 114300235 | - |
| IL36B | chr2 | ENST00000446595 | 114286223 | 114300235 | - |
| IL36G | chr2 | ENST00000446595 | 114286223 | 114300235 | - |
| IL37 | chr2 | ENST00000446595 | 114286223 | 114300235 | - |
| NR4A2 | chr2 | uc002tyw.2 | 156877047 | 157111432 | - |
| CXCR1 | chr2 | NR_002712 | 218923877 | 218926013 | - |
| CXCR2 | chr2 | NR_002712 | 218923877 | 218926013 | - |
| SLC11A1 | chr2 | NR_002712 | 218923877 | 218926013 | - |
| CCL20 | chr2 | uc002vpj.2 | 228549926 | 228582745 | - |
| UGT1A1 | chr2 | NR_037694 | 234662961 | 234663991 | - |
| HDAC4 | chr2 | ENST00000446029 | 240547391 | 240552533 | + |
| PPARG | chr3 | ENST00000499738 | 13054480 | 13057307 | + |
| MYD88 | chr3 | ENST00000438136 | 37795179 | 37903229 | - |
| GPX1 | chr3 | uc001azm.3 | 49721912 | 49722416 | - |
| HYAL1 | chr3 | uc001azm.3 | 49721912 | 49722416 | - |
| HYAL2 | chr3 | uc001azm.3 | 49721912 | 49722416 | - |
| HYAL3 | chr3 | uc001azm.3 | 49721912 | 49722416 | - |
| TUSC2 | chr3 | uc001azm.3 | 49721912 | 49722416 | - |
| GPX1 | chr3 | ENST00000456338 | 50242700 | 50256112 | + |
| HYAL1 | chr3 | ENST00000456338 | 50242700 | 50256112 | + |
| HYAL2 | chr3 | ENST00000456338 | 50242700 | 50256112 | + |
| HYAL3 | chr3 | ENST00000456338 | 50242700 | 50256112 | + |
| TUSC2 | chr3 | ENST00000456338 | 50242700 | 50256112 | + |
| GPX1 | chr3 | ENST00000439898 | 50297734 | 50300790 | + |
| HYAL1 | chr3 | ENST00000439898 | 50297734 | 50300790 | + |
| HYAL2 | chr3 | ENST00000439898 | 50297734 | 50300790 | + |
| HYAL3 | chr3 | ENST00000439898 | 50297734 | 50300790 | + |
| TUSC2 | chr3 | ENST00000439898 | 50297734 | 50300790 | + |
| PXK | chr3 | XLOC_002675 | 58419600 | 58470480 | + |
| PROK2 | chr3 | uc003dpd.1 | 72200407 | 72223483 | + |
| CD47 | chr3 | ASO1844 | 108106442 | 108108128 | + |
| CHST2 | chr3 | XLOC_002845 | 142645516 | 142661376 | + |
| AGTR1 | chr3 | XLOC_002865 | 148942374 | 148944127 | + |
| PTX3 | chr3 | NR_034007 | 156799455 | 156840791 | - |
| MECOM | chr3 | RNZ24353 | 168919598 | 168919836 | + |
| MECOM | chr3 | RNZ24358 | 168982583 | 168982790 | + |
| MECOM | chr3 | RNZ24362 | 169072712 | 169072912 | + |
| ALG3 | chr3 | XLOC_002948 | 184096017 | 184097565 | + |
| ALG3 | chr3 | RNZ24675 | 184374407 | 184374805 | + |
| ADIPOQ | chr3 | uc010hyn.1 | 185677757 | 185693547 | + |
| AHSG | chr3 | uc010hyn.1 | 185677757 | 185693547 | + |
| KNG1 | chr3 | uc010hyn.1 | 185677757 | 185693547 | + |
| LIAS | chr4 | uc003gun.1 | 39548920 | 39551206 | - |
| TLR1 | chr4 | uc003gun.1 | 39548920 | 39551206 | - |
| TLR10 | chr4 | uc003gun.1 | 39548920 | 39551206 | - |
| TLR6 | chr4 | uc003gun.1 | 39548920 | 39551206 | - |
| KIT | chr4 | RNZ25264 | 54729858 | 54730075 | + |
| CXCL1 | chr4 | RP11-622A1.2 | 74374519 | 74399845 | + |
| CXCL2 | chr4 | RP11-622A1.2 | 74374519 | 74399845 | + |
| CXCL3 | chr4 | RP11-622A1.2 | 74374519 | 74399845 | + |
| CXCL6 | chr4 | RP11-622A1.2 | 74374519 | 74399845 | + |
| IL8 | chr4 | RP11-622A1.2 | 74374519 | 74399845 | + |
| CXCL10 | chr4 | XLOC_003985 | 76103903 | 76105665 | - |
| CXCL11 | chr4 | XLOC_003985 | 76103903 | 76105665 | - |
| CXCL9 | chr4 | XLOC_003985 | 76103903 | 76105665 | - |
| NFKB1 | chr4 | XLOC_003629 | 104346198 | 104374914 | + |
| SMAD1 | chr4 | RNZ26024 | 146855638 | 146855838 | + |
| KLKB1 | chr4 | XLOC_003821 | 187137129 | 187137707 | + |
| TLR3 | chr4 | XLOC_003821 | 187137129 | 187137707 | + |
| KLKB1 | chr4 | NR_033900 | 187207251 | 187422212 | - |
| TLR3 | chr4 | NR_033900 | 187207251 | 187422212 | - |
| FAM105B | chr5 | RNZ24613 | 14962012 | 14962241 | + |
| ISL1 | chr5 | XLOC_004815 | 50261452 | 50266021 | - |
| IL6ST | chr5 | XLOC_004826 | 54895898 | 54899362 | - |
| NAIP | chr5 | uc011csc.1 | 70671790 | 70681768 | + |
| CDO1 | chr5 | XLOC_004959 | 114999014 | 115009135 | - |
| TICAM2 | chr5 | XLOC_004959 | 114999014 | 115009135 | - |
| TMED7-TICAM2 | chr5 | XLOC_004959 | 114999014 | 115009135 | - |
| CDO1 | chr5 | CTC-505O3.2 | 115074778 | 115092222 | + |
| TICAM2 | chr5 | CTC-505O3.2 | 115074778 | 115092222 | + |
| TMED7-TICAM2 | chr5 | CTC-505O3.2 | 115074778 | 115092222 | + |
| CDO1 | chr5 | ENST00000499037 | 115150891 | 115152356 | + |
| TICAM2 | chr5 | ENST00000499037 | 115150891 | 115152356 | + |
| TMED7-TICAM2 | chr5 | ENST00000499037 | 115150891 | 115152356 | + |
| IL13 | chr5 | ENST00000457890 | 131514926 | 131516821 | - |
| IL3 | chr5 | ENST00000457890 | 131514926 | 131516821 | - |
| IL4 | chr5 | ENST00000457890 | 131514926 | 131516821 | - |
| IL5 | chr5 | ENST00000457890 | 131514926 | 131516821 | - |
| IL9 | chr5 | HIT000067435.11 | 135465195 | 135470579 | - |
| HNRNPA0 | chr5 | NR_002713 | 137136881 | 137146439 | + |
| HNRNPA0 | chr5 | RNZ27716 | 137674323 | 137675451 | + |
| CD14 | chr5 | XLOC_004576 | 139529832 | 139531887 | + |
| CSF1R | chr5 | RNZ27942 | 148574976 | 148575244 | + |
| IL17B | chr5 | RNZ27942 | 148574976 | 148575244 | + |
| CSF1R | chr5 | ENST00000519898 | 148786478 | 148805225 | + |
| IL17B | chr5 | ENST00000519898 | 148786478 | 148805225 | + |
| IL12B | chr5 | AK021803 | 158499438 | 158500620 | - |
| IL12B | chr5 | ENST00000523301 | 158527629 | 158544486 | + |
| F12 | chr5 | ENST00000513271 | 176875052 | 176879142 | - |
| F12 | chr5 | XLOC_005099 | 177387461 | 177392855 | - |
| BMP6 | chr6 | XLOC_005158 | 8284310 | 8307763 | + |
| BMP6 | chr6 | NONE | 8652441 | 8654459 | + |
| HIST1H2BA | chr6 | ASO2281 | 25593014 | 25593574 | - |
| HIST1H2BA | chr6 | HIT000328221.9 | 25798603 | 25798901 | + |
| HIST1H2BA | chr6 | HIT000325594.4 | 25818263 | 25818644 | - |
| ABCF1 | chr6 | RNZ29018 | 30583811 | 30584192 | + |
| AIF1 | chr6 | RNZ29018 | 30583811 | 30584192 | + |
| IER3 | chr6 | RNZ29018 | 30583811 | 30584192 | + |
| LTA | chr6 | RNZ29018 | 30583811 | 30584192 | + |
| NCR3 | chr6 | RNZ29018 | 30583811 | 30584192 | + |
| TNF | chr6 | RNZ29018 | 30583811 | 30584192 | + |
| HLA-DRB1 | chr6 | RNZ29059 | 33379104 | 33379345 | + |
| IL17A | chr6 | NR_033760 | 52604260 | 52609957 | - |
| IL17F | chr6 | NR_033760 | 52604260 | 52609957 | - |
| MAS1 | chr6 | ASO3608 | 159463302 | 159469735 | + |
| MAS1 | chr6 | NR_028093 | 160887586 | 160932156 | - |
| HDAC9 | chr7 | ENST00000439046 | 17474100 | 17500296 | - |
| HDAC9 | chr7 | ENST00000451792 | 17503068 | 17598533 | - |
| NOD1 | chr7 | ENST00000447171 | 29554384 | 29603286 | - |
| TRIL | chr7 | ENST00000447171 | 29554384 | 29603286 | - |
| NOD1 | chr7 | NR_036554 | 29725687 | 29782019 | + |
| TRIL | chr7 | NR_036554 | 29725687 | 29782019 | + |
| NCF1 | chr7 | NR_026690 | 73149398 | 73150330 | + |
| SERPINE1 | chr7 | RNZ31467 | 101502627 | 101502864 | + |
| PIK3CG | chr7 | XLOC_006200 | 106415521 | 106421611 | + |
| RARRES2 | chr7 | XLOC_006293 | 150130741 | 150145228 | + |
| EPHX2 | chr8 | XLOC_006745 | 27029283 | 27068608 | + |
| PNMA2 | chr8 | XLOC_006745 | 27029283 | 27068608 | + |
| IDO1 | chr8 | NR_024106 | 39308563 | 39380470 | - |
| CRH | chr8 | XLOC_007110 | 67122576 | 67123886 | - |
| LY96 | chr8 | XLOC_006843 | 74005609 | 74006141 | + |
| RIPK2 | chr8 | uc003yed.2 | 90623584 | 90769797 | - |
| YWHAZ | chr8 | NR_033962 | 102064281 | 102088479 | + |
| YWHAZ | chr8 | HIT000393196.5 | 102633031 | 102633399 | + |
| IFNA2 | chr9 | NR_027054 | 21454266 | 21559697 | - |
| ANXA1 | chr9 | ENST00000449235 | 75486643 | 75489014 | - |
| S1PR3 | chr9 | XLOC_007443 | 90791286 | 90791995 | + |
| SYK | chr9 | XLOC_007456 | 94186603 | 94189429 | + |
| SYK | chr9 | RNZ34315 | 94658984 | 94659225 | + |
| KLF4 | chr9 | RNZ34485 | 109651288 | 109651566 | + |
| PTGES | chr9 | NR_028048 | 131857072 | 131873070 | - |
| GATA3 | chr10 | RNZ4591 | 8026401 | 8026601 | + |
| ALOX5 | chr10 | ENST00000453853 | 46798369 | 46809021 | - |
| MBL2 | chr10 | ENST00000443523 | 54316742 | 54515169 | - |
| CHID1 | chr11 | NR_026643 | 1686828 | 1689086 | + |
| DEAF1 | chr11 | NR_026643 | 1686828 | 1689086 | + |
| INS | chr11 | NR_026643 | 1686828 | 1689086 | + |
| TOLLIP | chr11 | NR_026643 | 1686828 | 1689086 | + |
| CHID1 | chr11 | NR_026642 | 1704499 | 1706859 | - |
| INS | chr11 | NR_026642 | 1704499 | 1706859 | - |
| TOLLIP | chr11 | NR_026642 | 1704499 | 1706859 | - |
| CHID1 | chr11 | uc001ltz.1 | 1709526 | 1710286 | + |
| INS | chr11 | uc001ltz.1 | 1709526 | 1710286 | + |
| TOLLIP | chr11 | uc001ltz.1 | 1709526 | 1710286 | + |
| INS | chr11 | ENST00000439725 | 2016405 | 2022700 | - |
| TOLLIP | chr11 | ENST00000439725 | 2016405 | 2022700 | - |
| INS | chr11 | LIT1660 | 2668474 | 2668897 | + |
| INS | chr11 | LIT1658 | 2696558 | 2696886 | + |
| CD44 | chr11 | RNZ6683 | 34653655 | 34653913 | + |
| CD59 | chr11 | RNZ6683 | 34653655 | 34653913 | + |
| F2 | chr11 | RNZ6858 | 47093391 | 47093793 | + |
| NR1H3 | chr11 | RNZ6858 | 47093391 | 47093793 | + |
| SERPING1 | chr11 | XLOC_009135 | 57386013 | 57388699 | + |
| GAL | chr11 | ENST00000533670 | 67653916 | 67673821 | + |
| GSTP1 | chr11 | ENST00000533670 | 67653916 | 67673821 | + |
| APOA1 | chr11 | ENST00000539222 | 115803574 | 115810928 | + |
| APOC3 | chr11 | ENST00000539222 | 115803574 | 115810928 | + |
| JAM3 | chr11 | ENST00000527712 | 133902699 | 133916744 | + |
| A2M | chr12 | uc009zgc.2 | 8332804 | 8356981 | + |
| C3AR1 | chr12 | uc009zgc.2 | 8332804 | 8356981 | + |
| CD163 | chr12 | uc009zgc.2 | 8332804 | 8356981 | + |
| KLRG1 | chr12 | uc009zgc.2 | 8332804 | 8356981 | + |
| A2M | chr12 | XLOC_002821 | 8404006 | 8450140 | + |
| C3AR1 | chr12 | XLOC_002821 | 8404006 | 8450140 | + |
| CD163 | chr12 | XLOC_002821 | 8404006 | 8450140 | + |
| KLRG1 | chr12 | XLOC_002821 | 8404006 | 8450140 | + |
| A2M | chr12 | XLOC_009653 | 9600968 | 9614245 | + |
| CLEC7A | chr12 | XLOC_009653 | 9600968 | 9614245 | + |
| KLRG1 | chr12 | XLOC_009653 | 9600968 | 9614245 | + |
| OLR1 | chr12 | XLOC_009653 | 9600968 | 9614245 | + |
| CLEC7A | chr12 | ENST00000500682 | 10516367 | 10551105 | + |
| OLR1 | chr12 | ENST00000500682 | 10516367 | 10551105 | + |
| CLEC7A | chr12 | uc009zhn.2 | 10741077 | 10752434 | - |
| OLR1 | chr12 | uc009zhn.2 | 10741077 | 10752434 | - |
| HDAC7 | chr12 | ENST00000547777 | 47641906 | 47643068 | + |
| HDAC7 | chr12 | ENST00000550019 | 47747536 | 47763718 | + |
| HDAC7 | chr12 | ENST00000547799 | 48099867 | 48136077 | + |
| CELA1 | chr12 | ASO1937 | 52203488 | 52204384 | - |
| NPFF | chr12 | NR_026655 | 54452037 | 54516018 | + |
| IL23A | chr12 | uc010sqx.1 | 57348915 | 57397270 | - |
| LTA4H | chr12 | uc009ztj.2 | 96405073 | 96408952 | - |
| MVK | chr12 | XLOC_009876 | 109029644 | 109036526 | + |
| MPHOSPH8 | chr13 | NR_027995 | 19408542 | 19446109 | - |
| HMGB1 | chr13 | RNZ9831 | 30160970 | 30161289 | + |
| ALOX5AP | chr13 | XLOC_010556 | 30914408 | 30951282 | - |
| HMGB1 | chr13 | XLOC_010556 | 30914408 | 30951282 | - |
| KL | chr13 | HIT000220172.7 | 33242538 | 33242992 | - |
| PRKD1 | chr14 | RNZ10900 | 30302933 | 30303152 | + |
| SERPINA1 | chr14 | XLOC_011107 | 95024729 | 95027008 | - |
| SERPINA3 | chr14 | XLOC_011107 | 95024729 | 95027008 | - |
| AK7 | chr14 | ENST00000554321 | 96343155 | 96389392 | + |
| BDKRB1 | chr14 | ENST00000554321 | 96343155 | 96389392 | + |
| BDKRB2 | chr14 | ENST00000554321 | 96343155 | 96389392 | + |
| AKT1 | chr14 | XLOC_010952 | 105559180 | 105565891 | + |
| RORA | chr15 | RNZ12639 | 61330498 | 61330698 | + |
| RORA | chr15 | RNZ12646 | 61413972 | 61414323 | + |
| SMAD3 | chr15 | XLOC_011510 | 67276459 | 67280258 | - |
| SMAD3 | chr15 | XLOC_011292 | 67332911 | 67340583 | + |
| SMAD3 | chr15 | RNZ12774 | 67367371 | 67367588 | + |
| CD276 | chr15 | XLOC_011309 | 74346639 | 74348558 | + |
| SEMA7A | chr15 | XLOC_011309 | 74346639 | 74348558 | + |
| VIMP | chr15 | XLOC_011390 | 101835622 | 101838894 | + |
| MEFV | chr16 | XLOC_011866 | 4230068 | 4233668 | - |
| IL27 | chr16 | uc010vdn.1 | 29086162 | 29128036 | + |
| LAT | chr16 | uc010vdn.1 | 29086162 | 29128036 | + |
| NUPR1 | chr16 | uc010vdn.1 | 29086162 | 29128036 | + |
| SPN | chr16 | uc010vdn.1 | 29086162 | 29128036 | + |
| NOD2 | chr16 | XLOC_011718 | 50913947 | 50918656 | + |
| NOD2 | chr16 | XLOC_011936 | 51096097 | 51129392 | - |
| NOD2 | chr16 | XLOC_011938 | 51421605 | 51559447 | - |
| CCL17 | chr16 | NR_001447 | 56651372 | 56652730 | + |
| CCL22 | chr16 | NR_001447 | 56651372 | 56652730 | + |
| CX3CL1 | chr16 | NR_001447 | 56651372 | 56652730 | + |
| CCL17 | chr16 | uc002ejp.1 | 56669674 | 56670998 | + |
| CCL22 | chr16 | uc002ejp.1 | 56669674 | 56670998 | + |
| CX3CL1 | chr16 | uc002ejp.1 | 56669674 | 56670998 | + |
| CCL17 | chr16 | NR_027781 | 56677598 | 56678853 | + |
| CCL22 | chr16 | NR_027781 | 56677598 | 56678853 | + |
| CX3CL1 | chr16 | NR_027781 | 56677598 | 56678853 | + |
| CCL17 | chr16 | uc010vhg.1 | 56710043 | 56711675 | + |
| CCL22 | chr16 | uc010vhg.1 | 56710043 | 56711675 | + |
| CX3CL1 | chr16 | uc010vhg.1 | 56710043 | 56711675 | + |
| ABR | chr17 | uc010cjm.1 | 181048 | 183234 | + |
| KDM6B | chr17 | uc010cno.1 | 7771138 | 7777031 | - |
| PER1 | chr17 | uc010cno.1 | 7771138 | 7777031 | - |
| AOC3 | chr17 | NR_002773 | 41019161 | 41021234 | + |
| STAT3 | chr17 | NR_002773 | 41019161 | 41021234 | + |
| STAT5A | chr17 | NR_002773 | 41019161 | 41021234 | + |
| STAT5B | chr17 | NR_002773 | 41019161 | 41021234 | + |
| NFE2L1 | chr17 | NR_027416 | 45500842 | 45504058 | + |
| NFE2L1 | chr17 | ENST00000438772 | 46684988 | 46716647 | - |
| NFE2L1 | chr17 | ENST00000433510 | 46713653 | 46724385 | - |
| HRH4 | chr18 | uc.424+ | 22767778 | 22767993 | + |
| MEP1B | chr18 | BC036040 | 29304160 | 29434944 | + |
| C3 | chr19 | XLOC_012931 | 6661465 | 6662832 | + |
| FFAR3 | chr19 | uc002nyj.1 | 35657757 | 35660784 | + |
| NFKBID | chr19 | uc002nyj.1 | 35657757 | 35660784 | + |
| FFAR3 | chr19 | XLOC_013054 | 36800485 | 36812260 | + |
| NFKBID | chr19 | XLOC_013054 | 36800485 | 36812260 | + |
| ZFP36 | chr19 | uc002olc.1 | 39840439 | 39843129 | + |
| AXL | chr19 | NR_001278 | 41430169 | 41456565 | + |
| RPS19 | chr19 | NR_001278 | 41430169 | 41456565 | + |
| TGFB1 | chr19 | NR_001278 | 41430169 | 41456565 | + |
| RPS19 | chr19 | NR_026824 | 43341148 | 43359870 | - |
| APOE | chr19 | XLOC_013093 | 44699118 | 44702389 | + |
| APOE | chr19 | NR_028412 | 45430059 | 45434281 | + |
| NANOS2 | chr19 | NR_028412 | 45430059 | 45434281 | + |
| BMP2 | chr20 | ENST00000428954 | 7237226 | 7238861 | - |
| HCK | chr20 | NR_003677 | 30135184 | 30136019 | + |
| ADA | chr20 | ASO3676 | 43000767 | 43031509 | - |
| ADA | chr20 | XLOC_013749 | 43077416 | 43079245 | - |
| ADA | chr20 | uc002xmm.1 | 43289247 | 43367608 | - |
| CD40 | chr20 | ENST00000419897 | 44649257 | 44650366 | - |
| TFF2 | chr21 | XLOC_013947 | 42953358 | 42954625 | + |
| TFF2 | chr21 | XLOC_013950 | 43194020 | 43196304 | + |
| ITGB2 | chr21 | ENST00000449713 | 45905459 | 45910171 | + |
| APOL2 | chr22 | uc003apj.1 | 36730923 | 36732334 | - |
| APOL3 | chr22 | uc003apj.1 | 36730923 | 36732334 | - |
| HMOX1 | chr22 | uc003apj.1 | 36730923 | 36732334 | - |
| NFAM1 | chr22 | XLOC_014390 | 43782059 | 43787399 | - |
| SCUBE1 | chr22 | XLOC_014390 | 43782059 | 43787399 | - |
| CYBB | chrX | XLOC_007952 | 37748182 | 37758208 | + |
