# Supplementary material for: Global identification and characterization of lncRNAs that control inflammation in malignant cholangiocytes
Source: BMC Genomics. 2018 Oct 11;19:735. doi: 10.1186/s12864-018-5133-8 (PMC6180422; doi:10.1186/s12864-018-5133-8)
Supplement: Supplementary file 9 — Table S6. Genome-wide correlation between lncRNA and mRNA levels (Pearson correlation coefficient > 0.95). (DOCX 221 kb) [file 12864_2018_5133_MOESM9_ESM.docx]

**Table S6.** **Genome-wide correlation between lncRNA and mRNA levels ( Pearson correlation coefficient> 0.95).**

| lncRNA | mRNA | Pearson r | lncRNA | mRNA | Pearson r |
| --- | --- | --- | --- | --- | --- |
| XLOC_012693 | A1BG | 0.972216478 | NRED_1255_416 | GLYAT | 0.954544 |
| XLOC_003572 | A1BG | 0.951120128 | NRED_1255_416 | GLYAT | 0.952823 |
| ENST00000507582 | A1BG | 0.956782718 | ENCODE_1597_545 | GLYAT | 0.955664 |
| ENST00000544018 | A1BG | 0.97147536 | ENCODE_2110_447 | GLYAT | 0.95048 |
| ENST00000417121 | A1BG | 0.960217081 | RefSeq_532_3763 | GLYAT | 0.982841 |
| ENST00000417851 | A1BG | 0.951684965 | RefSeq_1589_2127 | GLYAT | 0.95481 |
| ENST00000439524 | A1BG | 0.970575541 | RefSeq_1589_2127 | GLYAT | 0.95642 |
| ENST00000414604 | A1BG | 0.969736571 | RefSeq_2351_1430 | GLYAT | 0.971684 |
| ENST00000445096 | A1BG | 0.960236473 | UCSC_5722_1825 | GLYAT | 0.955514 |
| ENST00000545531 | A1BG | 0.957479551 | UCSC_6534_1640 | GLYAT | 0.966594 |
| RefSeq_532_3763 | A1BG | 0.981439424 | UCSC_8524_1151 | GLYAT | 0.957109 |
| RefSeq_2351_1430 | A1BG | 0.962302659 | UCSC_11594_306 | GLYAT | 0.954607 |
| UCSC_5722_1825 | A1BG | 0.96586488 | CombinedLit_370_438 | GLYAT | 0.979569 |
| UCSC_6534_1640 | A1BG | 0.9521082 | XLOC_012693 | GLYATL1 | 0.973001 |
| UCSC_11594_306 | A1BG | 0.956607699 | ENST00000417121 | GLYATL1 | 0.967674 |
| CombinedLit_370_438 | A1BG | 0.975210551 | ENST00000417851 | GLYATL1 | 0.959402 |
| ENST00000505626 | AADAT | 0.951687128 | ENST00000439524 | GLYATL1 | 0.959736 |
| ENCODE_1931_477 | AADAT | 0.966201255 | ENST00000414604 | GLYATL1 | 0.953165 |
| RefSeq_843_3032 | AADAT | 0.965058072 | ENST00000445096 | GLYATL1 | 0.968741 |
| RefSeq_869_3001 | AADAT | 0.95642547 | ENST00000545531 | GLYATL1 | 0.971228 |
| XLOC_012693 | ABAT | 0.952445385 | lncRNAdb_60_500 | GLYATL1 | 0.957249 |
| ENST00000417121 | ABAT | 0.964515448 | NRED_1255_416 | GLYATL1 | 0.953394 |
| ENST00000417851 | ABAT | 0.959834312 | ENCODE_2110_447 | GLYATL1 | 0.959905 |
| ENST00000439524 | ABAT | 0.965004994 | RefSeq_532_3763 | GLYATL1 | 0.96362 |
| ENST00000414604 | ABAT | 0.960250556 | RefSeq_2351_1430 | GLYATL1 | 0.962689 |
| ENST00000445096 | ABAT | 0.956063689 | UCSC_6534_1640 | GLYATL1 | 0.970873 |
| ENST00000545531 | ABAT | 0.964559831 | CombinedLit_370_438 | GLYATL1 | 0.965511 |
| RefSeq_2351_1430 | ABAT | 0.962567414 | ENST00000544018 | GNMT | 0.95924 |
| UCSC_6534_1640 | ABAT | 0.965566712 | ENST00000417121 | GOT1 | 0.967169 |
| UCSC_5722_1825 | ABCB11 | 0.967551874 | ENST00000417851 | GOT1 | 0.969764 |
| XLOC_004515 | ABCC2 | 0.962493072 | ENST00000439524 | GOT1 | 0.950253 |
| UCSC_4189_2198 | ABCC2 | 0.960805402 | ENST00000445096 | GOT1 | 0.958839 |
| UCSC_5722_1825 | ABCC2 | 0.961150245 | ENST00000545531 | GOT1 | 0.960189 |
| UCSC_5979_1768 | ABCC2 | 0.955338344 | RefSeq_2351_1430 | GOT1 | 0.962842 |
| ENST00000507582 | ABCC6 | 0.954600914 | UCSC_6534_1640 | GOT1 | 0.959902 |
| RefSeq_1040_2724 | ABCC6P1 | 0.980413409 | ENST00000417121 | GPLD1 | 0.954699 |
| asoverlaps_653_1337 | ABCC6P1 | 0.978560078 | ENST00000417851 | GPLD1 | 0.95654 |
| ENST00000538369 | ABCG5 | 0.955187268 | ENST00000545531 | GPLD1 | 0.953526 |
| ENST00000452481 | ABCG5 | 0.957344702 | UCSC_5722_1825 | GPLD1 | 0.953206 |
| UCSC_4189_2198 | ABCG5 | 0.95591963 | ENST00000439725 | H19 | 0.975926 |
| XLOC_004515 | ACADSB | 0.962984723 | ENST00000411861 | H19 | 0.974267 |
| XLOC_004515 | ACADSB | 0.953049179 | ENST00000417089 | H19 | 0.97002 |
| ENST00000507582 | ACADSB | 0.955233919 | ENST00000446406 | H19 | 0.976719 |
| ENST00000538369 | ACADSB | 0.954617655 | ENST00000431095 | H19 | 0.981794 |
| ENST00000544018 | ACADSB | 0.974667207 | ENST00000411754 | H19 | 0.978855 |
| ENST00000439524 | ACADSB | 0.952855882 | ENST00000422826 | H19 | 0.972106 |
| ENCODE_1597_545 | ACADSB | 0.969387204 | ENST00000428066 | H19 | 0.95526 |
| ENCODE_1597_545 | ACADSB | 0.968945866 | ENST00000436715 | H19 | 0.9919 |
| UCSC_5722_1825 | ACADSB | 0.951617746 | ENST00000442037 | H19 | 0.957727 |
| ENST00000507582 | ACAT1 | 0.95517051 | ENST00000535745 | H19 | 0.970072 |
| ENST00000538369 | ACAT1 | 0.967010959 | lncRNAdb_24_2322 | H19 | 0.980686 |
| ENST00000376608 | ACAT1 | 0.961375623 | UCSC_5536_1867 | H19 | 0.981244 |
| ENST00000376608 | ACAT1 | 0.952401643 | UCSC_6480_1652 | H19 | 0.981837 |
| ENCODE_2461_388 | ACAT1 | 0.956662277 | UCSC_6979_1542 | H19 | 0.972481 |
| RefSeq_532_3763 | ACAT1 | 0.955378096 | UCSC_7465_1417 | H19 | 0.978181 |
| RefSeq_1589_2127 | ACAT1 | 0.95340567 | UCSC_9805_863 | H19 | 0.979292 |
| UCSC_5210_1945 | ACAT1 | 0.963729924 | H-InvDB_974_445 | H19 | 0.975506 |
| UCSC_5722_1825 | ACAT1 | 0.960440756 | H-InvDB_1367_377 | H19 | 0.968313 |
| UCSC_5722_1825 | ACAT1 | 0.950313992 | CombinedLit_52_2251 | H19 | 0.978218 |
| UCSC_11594_306 | ACAT1 | 0.95447678 | NRED_705_1785 | HABP2 | 0.950489 |
| UCSC_4189_2198 | ACOT1 | 0.955079542 | RefSeq_843_3032 | HAL | 0.953592 |
| UCSC_5210_1945 | ACOT1 | 0.953079495 | XLOC_011486 | HAMP | 0.954727 |
| ENST00000417851 | ACOX2 | 0.958935556 | XLOC_012693 | HAO1 | 0.977458 |
| XLOC_012693 | ACSM2B | 0.982852541 | XLOC_003572 | HAO1 | 0.960317 |
| XLOC_003572 | ACSM2B | 0.964755904 | ENST00000507582 | HAO1 | 0.954935 |
| XLOC_004515 | ACSM2B | 0.958053717 | ENST00000544018 | HAO1 | 0.957371 |
| ENST00000507582 | ACSM2B | 0.955373929 | ENST00000417121 | HAO1 | 0.968132 |
| ENST00000544018 | ACSM2B | 0.951325778 | ENST00000417851 | HAO1 | 0.954953 |
| ENST00000417121 | ACSM2B | 0.976583309 | ENST00000439524 | HAO1 | 0.968683 |
| ENST00000417851 | ACSM2B | 0.961644919 | ENST00000414604 | HAO1 | 0.961017 |
| ENST00000439524 | ACSM2B | 0.978430149 | ENST00000445096 | HAO1 | 0.970385 |
| ENST00000414604 | ACSM2B | 0.969742542 | ENST00000545531 | HAO1 | 0.97287 |
| ENST00000445096 | ACSM2B | 0.978833504 | lncRNAdb_60_500 | HAO1 | 0.952831 |
| ENST00000545531 | ACSM2B | 0.978210349 | ENCODE_2110_447 | HAO1 | 0.964653 |
| lncRNAdb_60_500 | ACSM2B | 0.954064429 | RefSeq_532_3763 | HAO1 | 0.970765 |
| ENCODE_1597_545 | ACSM2B | 0.954527125 | RefSeq_2351_1430 | HAO1 | 0.95277 |
| ENCODE_2110_447 | ACSM2B | 0.961981154 | UCSC_6534_1640 | HAO1 | 0.957019 |
| RefSeq_532_3763 | ACSM2B | 0.974063782 | UCSC_11594_306 | HAO1 | 0.960947 |
| RefSeq_1769_1968 | ACSM2B | 0.953761842 | CombinedLit_370_438 | HAO1 | 0.966861 |
| RefSeq_2351_1430 | ACSM2B | 0.960701515 | XLOC_012693 | HAO2 | 0.971833 |
| UCSC_3328_2475 | ACSM2B | 0.951406317 | ENST00000544018 | HAO2 | 0.950596 |
| UCSC_5722_1825 | ACSM2B | 0.952851405 | ENST00000439524 | HAO2 | 0.959349 |
| UCSC_6534_1640 | ACSM2B | 0.959684196 | ENST00000414604 | HAO2 | 0.953346 |
| UCSC_8524_1151 | ACSM2B | 0.954675983 | ENCODE_1597_545 | HAO2 | 0.957973 |
| UCSC_11594_306 | ACSM2B | 0.967877886 | ENCODE_2110_447 | HAO2 | 0.952538 |
| CombinedLit_370_438 | ACSM2B | 0.977138858 | RefSeq_532_3763 | HAO2 | 0.969741 |
| XLOC_012693 | ACSM5 | 0.96945481 | RefSeq_1769_1968 | HAO2 | 0.965802 |
| XLOC_003572 | ACSM5 | 0.973252236 | UCSC_3328_2475 | HAO2 | 0.950738 |
| ENST00000507582 | ACSM5 | 0.959667158 | UCSC_8524_1151 | HAO2 | 0.95085 |
| ENST00000544018 | ACSM5 | 0.973836883 | UCSC_11594_306 | HAO2 | 0.95963 |
| ENST00000417121 | ACSM5 | 0.952630351 | CombinedLit_370_438 | HAO2 | 0.959155 |
| ENST00000439524 | ACSM5 | 0.968900783 | ENCODE_1931_477 | HFE2 | 0.953003 |
| ENST00000414604 | ACSM5 | 0.97202679 | RefSeq_843_3032 | HFE2 | 0.961395 |
| ENST00000445096 | ACSM5 | 0.96537361 | UCSC_3328_2475 | HFE2 | 0.952861 |
| ENST00000545531 | ACSM5 | 0.962193754 | XLOC_012693 | HGD | 0.967463 |
| NRED_1255_416 | ACSM5 | 0.953553728 | XLOC_004515 | HGD | 0.972215 |
| ENCODE_1597_545 | ACSM5 | 0.955923244 | ENST00000417121 | HGD | 0.954668 |
| ENCODE_2635_348 | ACSM5 | 0.973865817 | ENST00000439524 | HGD | 0.968932 |
| RefSeq_532_3763 | ACSM5 | 0.963736317 | ENST00000414604 | HGD | 0.960119 |
| RefSeq_1589_2127 | ACSM5 | 0.951722467 | ENST00000445096 | HGD | 0.957545 |
| UCSC_6534_1640 | ACSM5 | 0.955453745 | ENST00000545531 | HGD | 0.951972 |
| UCSC_6534_1640 | ACSM5 | 0.955212682 | ENCODE_1597_545 | HGD | 0.957082 |
| UCSC_10912_543 | ACSM5 | 0.964249648 | RefSeq_532_3763 | HGD | 0.951402 |
| CombinedLit_370_438 | ACSM5 | 0.964795729 | UCSC_4189_2198 | HGD | 0.953549 |
| XLOC_012693 | ADH4 | 0.965891901 | UCSC_5722_1825 | HGD | 0.950831 |
| ENST00000417121 | ADH4 | 0.956097477 | UCSC_6534_1640 | HGD | 0.952217 |
| ENST00000439524 | ADH4 | 0.980806 | CombinedLit_370_438 | HGD | 0.957224 |
| ENST00000414604 | ADH4 | 0.978687915 | XLOC_012693 | HGFAC | 0.978582 |
| ENST00000445096 | ADH4 | 0.959523261 | ENST00000417121 | HGFAC | 0.984803 |
| ENST00000545531 | ADH4 | 0.956021889 | ENST00000417851 | HGFAC | 0.978953 |
| RefSeq_532_3763 | ADH4 | 0.957750705 | ENST00000439524 | HGFAC | 0.967927 |
| UCSC_6534_1640 | ADH4 | 0.958938445 | ENST00000414604 | HGFAC | 0.960043 |
| CombinedLit_370_438 | ADH4 | 0.962547406 | ENST00000445096 | HGFAC | 0.985846 |
| XLOC_004515 | ADH6 | 0.951301576 | ENST00000545531 | HGFAC | 0.985493 |
| ENST00000439524 | ADH6 | 0.955360722 | lncRNAdb_60_500 | HGFAC | 0.95125 |
| UCSC_6534_1640 | ADH6 | 0.956480649 | RefSeq_532_3763 | HGFAC | 0.970901 |
| ENST00000544018 | ADRA1A | 0.959341359 | RefSeq_2351_1430 | HGFAC | 0.969314 |
| XLOC_012693 | AFM | 0.966360344 | UCSC_6534_1640 | HGFAC | 0.970967 |
| XLOC_003572 | AFM | 0.952186012 | CombinedLit_370_438 | HGFAC | 0.981096 |
| XLOC_004515 | AFM | 0.954647512 | XLOC_012693 | HOGA1 | 0.971211 |
| ENST00000507582 | AFM | 0.973123823 | ENST00000417121 | HOGA1 | 0.961968 |
| ENST00000538369 | AFM | 0.955757223 | ENST00000417851 | HOGA1 | 0.953617 |
| ENST00000417121 | AFM | 0.95438321 | ENST00000439524 | HOGA1 | 0.966782 |
| ENST00000439524 | AFM | 0.966797108 | ENST00000414604 | HOGA1 | 0.957333 |
| ENST00000414604 | AFM | 0.959412652 | ENST00000445096 | HOGA1 | 0.969677 |
| ENST00000445096 | AFM | 0.95837037 | ENST00000545531 | HOGA1 | 0.972096 |
| ENCODE_795_804 | AFM | 0.950081187 | lncRNAdb_60_500 | HOGA1 | 0.953397 |
| ENCODE_1597_545 | AFM | 0.955355052 | RefSeq_532_3763 | HOGA1 | 0.966393 |
| RefSeq_532_3763 | AFM | 0.956588902 | RefSeq_2351_1430 | HOGA1 | 0.961337 |
| RefSeq_843_3032 | AFM | 0.971835287 | UCSC_6534_1640 | HOGA1 | 0.973251 |
| RefSeq_869_3001 | AFM | 0.953398134 | UCSC_8524_1151 | HOGA1 | 0.951996 |
| RefSeq_1769_1968 | AFM | 0.967345956 | CombinedLit_370_438 | HOGA1 | 0.963999 |
| RefSeq_2866_939 | AFM | 0.959087632 | XLOC_012693 | HP | 0.953242 |
| RefSeq_3175_631 | AFM | 0.950290462 | RefSeq_532_3763 | HP | 0.97505 |
| UCSC_3328_2475 | AFM | 0.977733724 | RefSeq_2351_1430 | HP | 0.957879 |
| UCSC_4189_2198 | AFM | 0.955220558 | UCSC_8524_1151 | HP | 0.971781 |
| UCSC_5722_1825 | AFM | 0.959523414 | UCSC_11594_306 | HP | 0.950423 |
| UCSC_11594_306 | AFM | 0.972615315 | CombinedLit_370_438 | HP | 0.95877 |
| CombinedLit_370_438 | AFM | 0.959111325 | XLOC_012693 | HPD | 0.975381 |
| XLOC_012693 | AGXT | 0.989291195 | XLOC_001387 | HPD | 0.951676 |
| XLOC_003572 | AGXT | 0.952322648 | XLOC_004515 | HPD | 0.952584 |
| ENST00000544018 | AGXT | 0.951992379 | ENST00000507582 | HPD | 0.966817 |
| ENST00000417121 | AGXT | 0.986165471 | ENST00000538369 | HPD | 0.959848 |
| ENST00000417851 | AGXT | 0.975304321 | ENST00000544018 | HPD | 0.954932 |
| ENST00000439524 | AGXT | 0.982554683 | ENST00000376608 | HPD | 0.95354 |
| ENST00000414604 | AGXT | 0.97761337 | ENST00000417121 | HPD | 0.966695 |
| ENST00000445096 | AGXT | 0.986140631 | ENST00000417851 | HPD | 0.95977 |
| ENST00000545531 | AGXT | 0.985273065 | ENST00000439524 | HPD | 0.982464 |
| lncRNAdb_60_500 | AGXT | 0.962787572 | ENST00000414604 | HPD | 0.97488 |
| NRED_1255_416 | AGXT | 0.952854149 | ENST00000445096 | HPD | 0.965368 |
| ENCODE_2110_447 | AGXT | 0.953192337 | ENST00000545531 | HPD | 0.956201 |
| RefSeq_532_3763 | AGXT | 0.984086507 | lncRNAdb_60_500 | HPD | 0.95219 |
| RefSeq_1589_2127 | AGXT | 0.950756953 | ENCODE_795_804 | HPD | 0.953483 |
| RefSeq_2351_1430 | AGXT | 0.981518924 | ENCODE_2110_447 | HPD | 0.96981 |
| UCSC_6534_1640 | AGXT | 0.971017561 | RefSeq_532_3763 | HPD | 0.981476 |
| UCSC_8524_1151 | AGXT | 0.963385749 | RefSeq_1589_2127 | HPD | 0.959404 |
| UCSC_11594_306 | AGXT | 0.964575799 | RefSeq_1769_1968 | HPD | 0.955199 |
| CombinedLit_370_438 | AGXT | 0.992352139 | RefSeq_2351_1430 | HPD | 0.957096 |
| XLOC_004515 | AGXT2 | 0.965395912 | UCSC_5722_1825 | HPD | 0.974764 |
| ENST00000505626 | AGXT2 | 0.951077474 | UCSC_6534_1640 | HPD | 0.952726 |
| ENST00000507582 | AGXT2 | 0.952422987 | UCSC_11594_306 | HPD | 0.987457 |
| ENST00000538369 | AGXT2 | 0.962869899 | CombinedLit_370_438 | HPD | 0.96926 |
| ENST00000376608 | AGXT2 | 0.965687734 | XLOC_012693 | HPN | 0.960471 |
| ENCODE_2461_388 | AGXT2 | 0.957569615 | RefSeq_532_3763 | HPN | 0.958743 |
| RefSeq_843_3032 | AGXT2 | 0.953787916 | UCSC_8524_1151 | HPN | 0.956472 |
| RefSeq_869_3001 | AGXT2 | 0.95067358 | CombinedLit_370_438 | HPN | 0.95912 |
| RefSeq_1769_1968 | AGXT2 | 0.963685623 | XLOC_012693 | HPR | 0.966771 |
| UCSC_4189_2198 | AGXT2 | 0.970098499 | ENST00000417121 | HPR | 0.955686 |
| XLOC_012693 | AGXT2L1 | 0.97945363 | ENST00000439524 | HPR | 0.957319 |
| XLOC_003572 | AGXT2L1 | 0.95373692 | lncRNAdb_60_500 | HPR | 0.966938 |
| ENST00000417121 | AGXT2L1 | 0.985292372 | NRED_1255_416 | HPR | 0.952756 |
| ENST00000417851 | AGXT2L1 | 0.973992432 | RefSeq_532_3763 | HPR | 0.979249 |
| ENST00000439524 | AGXT2L1 | 0.971781042 | RefSeq_1589_2127 | HPR | 0.959541 |
| ENST00000414604 | AGXT2L1 | 0.960786189 | RefSeq_2351_1430 | HPR | 0.970876 |
| ENST00000445096 | AGXT2L1 | 0.981203225 | UCSC_8524_1151 | HPR | 0.981491 |
| ENST00000545531 | AGXT2L1 | 0.980739784 | UCSC_11594_306 | HPR | 0.969528 |
| lncRNAdb_60_500 | AGXT2L1 | 0.966576669 | CombinedLit_370_438 | HPR | 0.964283 |
| RefSeq_532_3763 | AGXT2L1 | 0.972514653 | XLOC_012693 | HPX | 0.991706 |
| RefSeq_1589_2127 | AGXT2L1 | 0.954407047 | XLOC_004515 | HPX | 0.952469 |
| RefSeq_2351_1430 | AGXT2L1 | 0.974562469 | ENST00000544018 | HPX | 0.960683 |
| UCSC_6534_1640 | AGXT2L1 | 0.965162059 | ENST00000417121 | HPX | 0.972929 |
| UCSC_8524_1151 | AGXT2L1 | 0.96788373 | ENST00000417851 | HPX | 0.95922 |
| UCSC_11594_306 | AGXT2L1 | 0.956111398 | ENST00000439524 | HPX | 0.983311 |
| CombinedLit_370_438 | AGXT2L1 | 0.981332913 | ENST00000414604 | HPX | 0.978895 |
| XLOC_012693 | AHSG | 0.989310613 | ENST00000445096 | HPX | 0.97517 |
| XLOC_003572 | AHSG | 0.955974328 | ENST00000545531 | HPX | 0.972254 |
| ENST00000417121 | AHSG | 0.967645106 | lncRNAdb_60_500 | HPX | 0.955701 |
| ENST00000417851 | AHSG | 0.954862733 | RefSeq_532_3763 | HPX | 0.994231 |
| ENST00000439524 | AHSG | 0.97457215 | RefSeq_1769_1968 | HPX | 0.953882 |
| ENST00000414604 | AHSG | 0.973532166 | RefSeq_2351_1430 | HPX | 0.972091 |
| ENST00000445096 | AHSG | 0.977253858 | UCSC_6534_1640 | HPX | 0.960793 |
| ENST00000545531 | AHSG | 0.968436336 | UCSC_8524_1151 | HPX | 0.970143 |
| lncRNAdb_60_500 | AHSG | 0.96116086 | UCSC_11594_306 | HPX | 0.974408 |
| RefSeq_532_3763 | AHSG | 0.985872052 | CombinedLit_370_438 | HPX | 0.991254 |
| RefSeq_2351_1430 | AHSG | 0.962372614 | XLOC_012693 | HRG | 0.980561 |
| UCSC_8524_1151 | AHSG | 0.965748019 | XLOC_003572 | HRG | 0.952941 |
| UCSC_11594_306 | AHSG | 0.965240958 | ENST00000507582 | HRG | 0.967863 |
| CombinedLit_370_438 | AHSG | 0.994168168 | ENST00000417121 | HRG | 0.960684 |
| ENST00000538369 | AIG1 | 0.951597934 | ENST00000439524 | HRG | 0.97995 |
| ENST00000417121 | AIG1 | 0.966553995 | ENST00000414604 | HRG | 0.976717 |
| ENST00000417851 | AIG1 | 0.96524677 | ENST00000445096 | HRG | 0.967052 |
| ENST00000445096 | AIG1 | 0.95627543 | ENST00000545531 | HRG | 0.952665 |
| ENST00000545531 | AIG1 | 0.959586568 | lncRNAdb_60_500 | HRG | 0.96448 |
| ENCODE_1597_545 | AIG1 | 0.957134324 | ENCODE_2110_447 | HRG | 0.965677 |
| UCSC_6534_1640 | AIG1 | 0.95258952 | RefSeq_532_3763 | HRG | 0.978997 |
| XLOC_001387 | AKR1CL1 | 0.950083764 | RefSeq_1769_1968 | HRG | 0.968406 |
| ENST00000507582 | AKR1CL1 | 0.9661982 | RefSeq_2351_1430 | HRG | 0.951385 |
| ENST00000538369 | AKR1CL1 | 0.959298656 | UCSC_5722_1825 | HRG | 0.957645 |
| ENST00000376608 | AKR1CL1 | 0.952276202 | UCSC_8524_1151 | HRG | 0.965407 |
| ENCODE_2461_388 | AKR1CL1 | 0.972746072 | UCSC_11594_306 | HRG | 0.991026 |
| UCSC_5722_1825 | AKR1CL1 | 0.953458065 | CombinedLit_370_438 | HRG | 0.983178 |
| UCSC_3328_2475 | AKR1D1 | 0.955083511 | XLOC_004515 | HRSP12 | 0.957901 |
| RefSeq_532_3763 | ALB | 0.955754164 | ENST00000538369 | HRSP12 | 0.966471 |
| UCSC_11594_306 | ALB | 0.952844677 | ENST00000376608 | HRSP12 | 0.954471 |
| NRED_1255_416 | ALDH1L1 | 0.95460112 | ENST00000452481 | HRSP12 | 0.952105 |
| RefSeq_1589_2127 | ALDH1L1 | 0.953495045 | ENCODE_2461_388 | HRSP12 | 0.955675 |
| UCSC_6534_1640 | ALDH1L1 | 0.969623997 | UCSC_4189_2198 | HRSP12 | 0.955172 |
| UCSC_6631_1619 | ALDH4A1 | 0.952507936 | UCSC_5210_1945 | HRSP12 | 0.959408 |
| UCSC_8286_1209 | ALDH4A1 | 0.952832469 | ENST00000544018 | HS3ST3B1 | 0.973888 |
| XLOC_012693 | ALDH6A1 | 0.956029023 | XLOC_004515 | HSD17B13 | 0.953669 |
| XLOC_002845 | ALDH6A1 | 0.962847405 | ENST00000505626 | HSD17B13 | 0.957266 |
| XLOC_004515 | ALDH6A1 | 0.970454422 | ENST00000507582 | HSD17B13 | 0.966591 |
| ENST00000417121 | ALDH6A1 | 0.968943059 | ENCODE_1597_545 | HSD17B13 | 0.963246 |
| ENST00000417851 | ALDH6A1 | 0.958721372 | RefSeq_843_3032 | HSD17B13 | 0.971206 |
| ENST00000439524 | ALDH6A1 | 0.96145115 | RefSeq_869_3001 | HSD17B13 | 0.958338 |
| ENST00000445096 | ALDH6A1 | 0.959270195 | RefSeq_1769_1968 | HSD17B13 | 0.965055 |
| ENST00000545531 | ALDH6A1 | 0.968467886 | UCSC_3328_2475 | HSD17B13 | 0.966154 |
| ENCODE_1597_545 | ALDH6A1 | 0.96083194 | UCSC_5210_1945 | HSD17B4 | 0.966172 |
| RefSeq_1589_2127 | ALDH6A1 | 0.951473764 | UCSC_11594_306 | HSD17B4 | 0.955466 |
| UCSC_4189_2198 | ALDH6A1 | 0.951532914 | XLOC_012693 | HSD17B6 | 0.961052 |
| UCSC_5722_1825 | ALDH6A1 | 0.961011442 | XLOC_001387 | HSD17B6 | 0.955623 |
| UCSC_6534_1640 | ALDH6A1 | 0.96560662 | XLOC_002845 | HSD17B6 | 0.954509 |
| XLOC_004515 | ALDH8A1 | 0.959008054 | XLOC_004515 | HSD17B6 | 0.977663 |
| ENST00000507582 | ALDH8A1 | 0.950182579 | ENST00000507582 | HSD17B6 | 0.963889 |
| ENST00000538369 | ALDH8A1 | 0.972407482 | ENST00000538369 | HSD17B6 | 0.975677 |
| ENST00000376608 | ALDH8A1 | 0.958705826 | ENST00000544018 | HSD17B6 | 0.962424 |
| ENST00000452481 | ALDH8A1 | 0.962001408 | ENST00000376608 | HSD17B6 | 0.954764 |
| ENCODE_2110_447 | ALDH8A1 | 0.961920793 | ENST00000439524 | HSD17B6 | 0.97184 |
| RefSeq_532_3763 | ALDH8A1 | 0.950833935 | ENST00000414604 | HSD17B6 | 0.963753 |
| UCSC_4189_2198 | ALDH8A1 | 0.95820738 | ENCODE_456_1286 | HSD17B6 | 0.952508 |
| UCSC_5210_1945 | ALDH8A1 | 0.950916057 | ENCODE_1597_545 | HSD17B6 | 0.971197 |
| UCSC_5722_1825 | ALDH8A1 | 0.951302643 | RefSeq_532_3763 | HSD17B6 | 0.954736 |
| UCSC_10539_647 | ALDH8A1 | 0.953897802 | RefSeq_1769_1968 | HSD17B6 | 0.959331 |
| UCSC_11594_306 | ALDH8A1 | 0.962092461 | UCSC_4189_2198 | HSD17B6 | 0.963453 |
| XLOC_012693 | AMBP | 0.965991062 | UCSC_5722_1825 | HSD17B6 | 0.96816 |
| ENST00000439524 | AMBP | 0.950303538 | UCSC_11594_306 | HSD17B6 | 0.95253 |
| ENST00000445096 | AMBP | 0.95156576 | XLOC_012693 | HULC | 0.972971 |
| RefSeq_532_3763 | AMBP | 0.972833693 | ENST00000417121 | HULC | 0.957655 |
| UCSC_6534_1640 | AMBP | 0.950446657 | ENST00000417851 | HULC | 0.952854 |
| CombinedLit_370_438 | AMBP | 0.958143209 | ENST00000445096 | HULC | 0.957849 |
| XLOC_012693 | AMDHD1 | 0.97087776 | lncRNAdb_60_500 | HULC | 0.99826 |
| XLOC_001387 | AMDHD1 | 0.95095149 | NRED_1255_416 | HULC | 0.955613 |
| XLOC_002845 | AMDHD1 | 0.952932834 | ENCODE_2110_447 | HULC | 0.962822 |
| XLOC_003572 | AMDHD1 | 0.954816268 | RefSeq_532_3763 | HULC | 0.957459 |
| XLOC_003629 | AMDHD1 | 0.958619038 | RefSeq_2351_1430 | HULC | 0.955965 |
| XLOC_004515 | AMDHD1 | 0.983113823 | UCSC_8524_1151 | HULC | 0.96534 |
| ENST00000507582 | AMDHD1 | 0.958891909 | UCSC_11594_306 | HULC | 0.962925 |
| ENST00000538369 | AMDHD1 | 0.953358672 | CombinedLit_370_438 | HULC | 0.957912 |
| ENST00000544018 | AMDHD1 | 0.965477479 | lncRNAdb_60_500 | IGFBP1 | 0.950645 |
| ENST00000376608 | AMDHD1 | 0.958849777 | UCSC_8524_1151 | IGFBP1 | 0.968643 |
| ENST00000439524 | AMDHD1 | 0.964014685 | H-InvDB_1158_407 | IGHV1-18 | 0.966397 |
| ENST00000414604 | AMDHD1 | 0.950977857 | H-InvDB_1160_407 | IGHV1-18 | 0.968761 |
| ENCODE_795_804 | AMDHD1 | 0.953406484 | H-InvDB_1712_333 | IGHV3-66 | 0.964068 |
| ENCODE_1597_545 | AMDHD1 | 0.967311838 | H-InvDB_1058_425 | IGHV5-51 | 0.981858 |
| ENCODE_2110_447 | AMDHD1 | 0.956253166 | H-InvDB_1327_382 | IGHV5-51 | 0.988247 |
| RefSeq_532_3763 | AMDHD1 | 0.978805134 | H-InvDB_1402_371 | IGLL5 | 0.952429 |
| RefSeq_1589_2127 | AMDHD1 | 0.950177234 | H-InvDB_1786_325 | IGLV3-25 | 0.954023 |
| RefSeq_1769_1968 | AMDHD1 | 0.961190411 | H-InvDB_373_650 | IGLV6-57 | 0.959722 |
| UCSC_3328_2475 | AMDHD1 | 0.953511081 | XLOC_012693 | INHBC | 0.991057 |
| UCSC_4189_2198 | AMDHD1 | 0.962025033 | XLOC_003572 | INHBC | 0.955564 |
| UCSC_5722_1825 | AMDHD1 | 0.966415821 | ENST00000507582 | INHBC | 0.955496 |
| UCSC_6534_1640 | AMDHD1 | 0.952039723 | ENST00000544018 | INHBC | 0.950521 |
| UCSC_8524_1151 | AMDHD1 | 0.953149175 | ENST00000417121 | INHBC | 0.97216 |
| UCSC_11594_306 | AMDHD1 | 0.969646152 | ENST00000417851 | INHBC | 0.958571 |
| CombinedLit_370_438 | AMDHD1 | 0.954822877 | ENST00000439524 | INHBC | 0.981794 |
| XLOC_000915 | AMHR2 | 0.956044599 | ENST00000414604 | INHBC | 0.977973 |
| XLOC_004515 | ANG | 0.960597698 | ENST00000445096 | INHBC | 0.97626 |
| ENCODE_1597_545 | ANG | 0.956603085 | ENST00000545531 | INHBC | 0.967563 |
| RefSeq_2866_939 | ANG | 0.95400005 | lncRNAdb_60_500 | INHBC | 0.965247 |
| UCSC_5722_1825 | ANG | 0.954217171 | NRED_1255_416 | INHBC | 0.951258 |
| ENST00000507582 | ANGPTL3 | 0.953165525 | RefSeq_532_3763 | INHBC | 0.991396 |
| ENST00000538369 | ANGPTL3 | 0.950930834 | RefSeq_1589_2127 | INHBC | 0.951268 |
| ENST00000376608 | ANGPTL3 | 0.958625994 | RefSeq_1769_1968 | INHBC | 0.956696 |
| ENCODE_1931_477 | ANGPTL3 | 0.950736307 | RefSeq_2351_1430 | INHBC | 0.9677 |
| ENCODE_2110_447 | ANGPTL3 | 0.954718772 | UCSC_6534_1640 | INHBC | 0.956433 |
| ENCODE_2461_388 | ANGPTL3 | 0.95170326 | UCSC_8524_1151 | INHBC | 0.975655 |
| RefSeq_843_3032 | ANGPTL3 | 0.967120509 | UCSC_11594_306 | INHBC | 0.977189 |
| RefSeq_869_3001 | ANGPTL3 | 0.953786817 | CombinedLit_370_438 | INHBC | 0.99371 |
| UCSC_5210_1945 | ANGPTL3 | 0.959523162 | XLOC_012693 | ITIH1 | 0.986144 |
| UCSC_11594_306 | ANGPTL3 | 0.962853808 | ENST00000417121 | ITIH1 | 0.963825 |
| XLOC_012784 | ANKRD30BP2 | 0.964590464 | ENST00000439524 | ITIH1 | 0.96772 |
| UCSC_8158_1240 | ANKRD30BP2 | 0.970421489 | ENST00000414604 | ITIH1 | 0.96451 |
| ENST00000544018 | AOX1 | 0.952638397 | ENST00000445096 | ITIH1 | 0.968543 |
| RefSeq_532_3763 | AOX1 | 0.950747706 | ENST00000545531 | ITIH1 | 0.962645 |
| XLOC_012693 | APOA1 | 0.965052169 | lncRNAdb_60_500 | ITIH1 | 0.966829 |
| ENST00000417121 | APOA1 | 0.95822768 | RefSeq_532_3763 | ITIH1 | 0.987341 |
| ENST00000417851 | APOA1 | 0.951723141 | RefSeq_2351_1430 | ITIH1 | 0.963329 |
| ENST00000417851 | APOA1 | 0.951853005 | UCSC_8524_1151 | ITIH1 | 0.974235 |
| ENST00000439524 | APOA1 | 0.980011108 | UCSC_11594_306 | ITIH1 | 0.974704 |
| ENST00000414604 | APOA1 | 0.97690008 | CombinedLit_370_438 | ITIH1 | 0.991251 |
| ENST00000445096 | APOA1 | 0.962970919 | XLOC_012693 | ITIH2 | 0.95809 |
| ENST00000545531 | APOA1 | 0.950679084 | RefSeq_532_3763 | ITIH2 | 0.962746 |
| NRED_1255_416 | APOA1 | 0.964815079 | RefSeq_1769_1968 | ITIH2 | 0.950885 |
| RefSeq_532_3763 | APOA1 | 0.960289318 | UCSC_8524_1151 | ITIH2 | 0.958519 |
| RefSeq_1589_2127 | APOA1 | 0.950770938 | UCSC_11594_306 | ITIH2 | 0.961378 |
| UCSC_6534_1640 | APOA1 | 0.958162148 | CombinedLit_370_438 | ITIH2 | 0.962055 |
| UCSC_6534_1640 | APOA1 | 0.952831893 | XLOC_012693 | ITIH3 | 0.972807 |
| CombinedLit_370_438 | APOA1 | 0.959839006 | XLOC_003572 | ITIH3 | 0.951699 |
| XLOC_012693 | APOA2 | 0.959002895 | ENST00000544018 | ITIH3 | 0.959363 |
| RefSeq_532_3763 | APOA2 | 0.972487531 | ENST00000417121 | ITIH3 | 0.952216 |
| RefSeq_1769_1968 | APOA2 | 0.951135929 | ENST00000439524 | ITIH3 | 0.962455 |
| UCSC_11594_306 | APOA2 | 0.985566558 | ENST00000414604 | ITIH3 | 0.961544 |
| CombinedLit_370_438 | APOA2 | 0.966923984 | ENST00000445096 | ITIH3 | 0.954844 |
| XLOC_012693 | APOA5 | 0.973917398 | RefSeq_532_3763 | ITIH3 | 0.982302 |
| XLOC_003572 | APOA5 | 0.966730199 | RefSeq_2351_1430 | ITIH3 | 0.951988 |
| ENST00000507582 | APOA5 | 0.965668428 | UCSC_5722_1825 | ITIH3 | 0.954529 |
| ENST00000547552 | APOA5 | 0.953572384 | UCSC_11594_306 | ITIH3 | 0.964838 |
| ENST00000417121 | APOA5 | 0.95009849 | CombinedLit_370_438 | ITIH3 | 0.97336 |
| ENST00000439524 | APOA5 | 0.961023499 | XLOC_012693 | ITIH4 | 0.98782 |
| ENST00000414604 | APOA5 | 0.957368096 | ENST00000417121 | ITIH4 | 0.968979 |
| ENST00000445096 | APOA5 | 0.964970147 | ENST00000417851 | ITIH4 | 0.950825 |
| lncRNAdb_60_500 | APOA5 | 0.95151321 | ENST00000439524 | ITIH4 | 0.978063 |
| ENCODE_2110_447 | APOA5 | 0.953177069 | ENST00000414604 | ITIH4 | 0.974692 |
| RefSeq_532_3763 | APOA5 | 0.959231692 | ENST00000445096 | ITIH4 | 0.974042 |
| RefSeq_1769_1968 | APOA5 | 0.967230841 | ENST00000545531 | ITIH4 | 0.965338 |
| UCSC_3328_2475 | APOA5 | 0.967246453 | lncRNAdb_60_500 | ITIH4 | 0.953786 |
| UCSC_11594_306 | APOA5 | 0.971066862 | RefSeq_532_3763 | ITIH4 | 0.986021 |
| CombinedLit_370_438 | APOA5 | 0.971532627 | RefSeq_1769_1968 | ITIH4 | 0.956812 |
| UCSC_11352_391 | APOC1 | 0.978938519 | RefSeq_2351_1430 | ITIH4 | 0.96178 |
| XLOC_012693 | APOC2 | 0.968063957 | UCSC_8524_1151 | ITIH4 | 0.965214 |
| ENST00000417121 | APOC2 | 0.957603757 | UCSC_11594_306 | ITIH4 | 0.9766 |
| ENST00000439524 | APOC2 | 0.958346304 | CombinedLit_370_438 | ITIH4 | 0.994631 |
| ENST00000414604 | APOC2 | 0.950212287 | XLOC_012693 | KIAA0087 | 0.952403 |
| ENST00000445096 | APOC2 | 0.954944352 | XLOC_003572 | KIAA0087 | 0.954297 |
| ENST00000545531 | APOC2 | 0.950388664 | ENST00000439524 | KIAA0087 | 0.951881 |
| lncRNAdb_60_500 | APOC2 | 0.95376314 | ENST00000414604 | KIAA0087 | 0.959718 |
| RefSeq_532_3763 | APOC2 | 0.965400927 | UCSC_3328_2475 | KIAA0087 | 0.951086 |
| UCSC_8524_1151 | APOC2 | 0.974191584 | CombinedLit_370_438 | KIAA0087 | 0.958108 |
| UCSC_11594_306 | APOC2 | 0.956494612 | ENST00000452481 | KLB | 0.956541 |
| CombinedLit_370_438 | APOC2 | 0.97426875 | UCSC_10539_647 | KLB | 0.958086 |
| XLOC_012693 | APOC3 | 0.955618676 | XLOC_012693 | KNG1 | 0.981028 |
| ENST00000439524 | APOC3 | 0.976155938 | XLOC_003572 | KNG1 | 0.953235 |
| ENST00000414604 | APOC3 | 0.97221355 | ENST00000507582 | KNG1 | 0.952581 |
| RefSeq_532_3763 | APOC3 | 0.959338117 | ENST00000417121 | KNG1 | 0.962278 |
| UCSC_11594_306 | APOC3 | 0.95740306 | ENST00000417851 | KNG1 | 0.954665 |
| CombinedLit_370_438 | APOC3 | 0.952773864 | ENST00000439524 | KNG1 | 0.979243 |
| XLOC_012693 | APOC4 | 0.995632638 | ENST00000414604 | KNG1 | 0.974778 |
| XLOC_003572 | APOC4 | 0.957495094 | ENST00000445096 | KNG1 | 0.967703 |
| ENST00000544018 | APOC4 | 0.956306455 | ENST00000545531 | KNG1 | 0.951012 |
| ENST00000417121 | APOC4 | 0.967430284 | lncRNAdb_60_500 | KNG1 | 0.953216 |
| ENST00000417851 | APOC4 | 0.955771579 | RefSeq_532_3763 | KNG1 | 0.987149 |
| ENST00000439524 | APOC4 | 0.980620757 | RefSeq_1769_1968 | KNG1 | 0.960954 |
| ENST00000414604 | APOC4 | 0.978578595 | UCSC_5722_1825 | KNG1 | 0.953345 |
| ENST00000445096 | APOC4 | 0.976907234 | UCSC_8524_1151 | KNG1 | 0.958825 |
| ENST00000545531 | APOC4 | 0.966815694 | UCSC_11594_306 | KNG1 | 0.984936 |
| lncRNAdb_60_500 | APOC4 | 0.958262227 | CombinedLit_370_438 | KNG1 | 0.981634 |
| RefSeq_532_3763 | APOC4 | 0.988949808 | XLOC_012693 | LECT2 | 0.978275 |
| RefSeq_1769_1968 | APOC4 | 0.957513908 | ENST00000417121 | LECT2 | 0.950949 |
| RefSeq_2351_1430 | APOC4 | 0.959199336 | ENST00000439524 | LECT2 | 0.959144 |
| UCSC_6534_1640 | APOC4 | 0.954562959 | ENST00000414604 | LECT2 | 0.951273 |
| UCSC_8524_1151 | APOC4 | 0.95955539 | ENST00000445096 | LECT2 | 0.956989 |
| UCSC_11594_306 | APOC4 | 0.971142993 | lncRNAdb_60_500 | LECT2 | 0.98234 |
| CombinedLit_370_438 | APOC4 | 0.991069788 | ENCODE_2110_447 | LECT2 | 0.960112 |
| XLOC_012693 | APOF | 0.979936379 | RefSeq_532_3763 | LECT2 | 0.976603 |
| XLOC_004515 | APOF | 0.957797008 | RefSeq_2351_1430 | LECT2 | 0.960398 |
| ENST00000439524 | APOF | 0.961756176 | UCSC_8524_1151 | LECT2 | 0.981488 |
| ENST00000414604 | APOF | 0.956774873 | UCSC_11594_306 | LECT2 | 0.976538 |
| ENST00000445096 | APOF | 0.953730795 | CombinedLit_370_438 | LECT2 | 0.976699 |
| ENCODE_795_804 | APOF | 0.95225201 | ENST00000507582 | LIPC | 0.95012 |
| ENCODE_1597_545 | APOF | 0.950673159 | RefSeq_1992_1751 | LMF1 | 0.984995 |
| RefSeq_532_3763 | APOF | 0.96950202 | UCSC_1952_3222 | LMF1 | 0.980851 |
| RefSeq_1769_1968 | APOF | 0.968675471 | XLOC_001387 | LOC100129278 | 0.98023 |
| UCSC_3328_2475 | APOF | 0.968136876 | UCSC_5722_1825 | LOC100129278 | 0.958375 |
| UCSC_11594_306 | APOF | 0.968339506 | UCSC_10912_543 | LOC100129278 | 0.950438 |
| CombinedLit_370_438 | APOF | 0.966824017 | H-InvDB_1311_384 | LOC100290481 | 0.953394 |
| XLOC_012693 | APOH | 0.975165894 | H-InvDB_1544_351 | LOC100290481 | 0.963149 |
| XLOC_003572 | APOH | 0.95358637 | H-InvDB_1748_330 | LOC100290481 | 0.952098 |
| ENST00000439524 | APOH | 0.956808585 | XLOC_012693 | LOC255167 | 0.956433 |
| ENST00000414604 | APOH | 0.95052837 | XLOC_003572 | LOC255167 | 0.959715 |
| ENST00000445096 | APOH | 0.951535953 | RefSeq_532_3763 | LOC255167 | 0.950541 |
| lncRNAdb_60_500 | APOH | 0.955293264 | ENST00000439524 | LOC285733 | 0.951419 |
| RefSeq_532_3763 | APOH | 0.985234037 | XLOC_010898 | LOC339926 | 0.959004 |
| RefSeq_1769_1968 | APOH | 0.950218837 | EvoFold_309_241 | LOC339926 | 0.951361 |
| UCSC_8524_1151 | APOH | 0.972212067 | XLOC_008833 | LOC440297 | 0.972492 |
| UCSC_11594_306 | APOH | 0.973841759 | RefSeq_91_6807 | LOC440297 | 0.990631 |
| CombinedLit_370_438 | APOH | 0.9777588 | RefSeq_92_6806 | LOC440297 | 0.98847 |
| XLOC_012693 | APOM | 0.977320036 | NRED_1152_890 | LOC440335 | 0.959289 |
| XLOC_012693 | APOM | 0.955603446 | NRED_1261_364 | LOC440335 | 0.966141 |
| XLOC_004515 | APOM | 0.95592038 | RefSeq_3361_394 | LOC440335 | 0.966517 |
| XLOC_004515 | APOM | 0.958618947 | ENCODE_1230_628 | LOC553137 | 0.962464 |
| ENST00000507582 | APOM | 0.96387256 | XLOC_003629 | LOC645277 | 0.951665 |
| ENST00000507582 | APOM | 0.951581104 | XLOC_001387 | LOC731656 | 0.962087 |
| ENST00000538369 | APOM | 0.958260748 | XLOC_002845 | LOC731656 | 0.953256 |
| ENST00000544018 | APOM | 0.957112113 | ENST00000507582 | LOC731656 | 0.96079 |
| ENST00000417121 | APOM | 0.9759854 | ENST00000538369 | LOC731656 | 0.964156 |
| ENST00000417121 | APOM | 0.960842694 | ENST00000376608 | LOC731656 | 0.974573 |
| ENST00000417851 | APOM | 0.965220355 | ENCODE_2110_447 | LOC731656 | 0.957619 |
| ENST00000417851 | APOM | 0.952612469 | UCSC_5722_1825 | LOC731656 | 0.973955 |
| ENST00000439524 | APOM | 0.989954126 | UCSC_11594_306 | LOC731656 | 0.95389 |
| ENST00000439524 | APOM | 0.967298572 | UCSC_3291_2491 | LOC732156 | 0.954318 |
| ENST00000414604 | APOM | 0.986623814 | H-InvDB_1963_311 | LOC96610 | 0.960293 |
| ENST00000414604 | APOM | 0.954632308 | XLOC_004515 | LPA | 0.950003 |
| ENST00000445096 | APOM | 0.977095354 | XLOC_004515 | LPA | 0.956845 |
| ENST00000445096 | APOM | 0.954764646 | ENCODE_2360_408 | LPA | 0.951311 |
| ENST00000545531 | APOM | 0.971804287 | RefSeq_1769_1968 | LPA | 0.960883 |
| ENCODE_1597_545 | APOM | 0.961632842 | XLOC_012693 | LPAL2 | 0.955139 |
| ENCODE_1597_545 | APOM | 0.958125494 | XLOC_004515 | LPAL2 | 0.952125 |
| RefSeq_532_3763 | APOM | 0.967332309 | ENCODE_2360_408 | LPAL2 | 0.950267 |
| RefSeq_1769_1968 | APOM | 0.953061345 | RefSeq_532_3763 | LPAL2 | 0.955331 |
| RefSeq_2351_1430 | APOM | 0.95990615 | RefSeq_1769_1968 | LPAL2 | 0.968124 |
| RefSeq_2866_939 | APOM | 0.954435193 | UCSC_8524_1151 | LPAL2 | 0.950016 |
| RefSeq_3051_754 | APOM | 0.950436447 | UCSC_11594_306 | LPAL2 | 0.957386 |
| UCSC_4189_2198 | APOM | 0.963624889 | XLOC_012693 | LRG1 | 0.971435 |
| UCSC_5210_1945 | APOM | 0.95044934 | XLOC_004515 | LRG1 | 0.959552 |
| UCSC_5722_1825 | APOM | 0.969102713 | ENST00000439524 | LRG1 | 0.959513 |
| UCSC_5722_1825 | APOM | 0.96723925 | ENST00000414604 | LRG1 | 0.953772 |
| UCSC_6534_1640 | APOM | 0.955358678 | RefSeq_532_3763 | LRG1 | 0.966877 |
| UCSC_11594_306 | APOM | 0.964098846 | RefSeq_1769_1968 | LRG1 | 0.955656 |
| CombinedLit_370_438 | APOM | 0.97808281 | UCSC_8524_1151 | LRG1 | 0.957914 |
| UCSC_10664_614 | AQP7P3 | 0.954277504 | UCSC_11594_306 | LRG1 | 0.955966 |
| RefSeq_869_3001 | AR | 0.950176768 | CombinedLit_370_438 | LRG1 | 0.962928 |
| XLOC_012693 | ARG1 | 0.994030134 | XLOC_012693 | MASP1 | 0.975988 |
| XLOC_012693 | ARG1 | 0.985735095 | XLOC_003572 | MASP1 | 0.956271 |
| XLOC_003572 | ARG1 | 0.953760968 | XLOC_004515 | MASP1 | 0.96444 |
| XLOC_004515 | ARG1 | 0.952209201 | ENST00000417121 | MASP1 | 0.968651 |
| XLOC_004515 | ARG1 | 0.963989097 | ENST00000417851 | MASP1 | 0.95599 |
| ENST00000544018 | ARG1 | 0.958748099 | ENST00000439524 | MASP1 | 0.965587 |
| ENST00000544018 | ARG1 | 0.951416007 | ENST00000414604 | MASP1 | 0.952419 |
| ENST00000417121 | ARG1 | 0.980170783 | ENST00000445096 | MASP1 | 0.970736 |
| ENST00000417121 | ARG1 | 0.975189672 | ENST00000545531 | MASP1 | 0.964774 |
| ENST00000417851 | ARG1 | 0.97245551 | lncRNAdb_60_500 | MASP1 | 0.962144 |
| ENST00000417851 | ARG1 | 0.96700758 | ENCODE_1597_545 | MASP1 | 0.953828 |
| ENST00000439524 | ARG1 | 0.984770767 | ENCODE_2110_447 | MASP1 | 0.950024 |
| ENST00000439524 | ARG1 | 0.975397599 | RefSeq_532_3763 | MASP1 | 0.964962 |
| ENST00000414604 | ARG1 | 0.978441932 | RefSeq_843_3032 | MASP1 | 0.952804 |
| ENST00000414604 | ARG1 | 0.961666333 | UCSC_3328_2475 | MASP1 | 0.952415 |
| ENST00000445096 | ARG1 | 0.982206465 | UCSC_4189_2198 | MASP1 | 0.959173 |
| ENST00000445096 | ARG1 | 0.971107955 | UCSC_5722_1825 | MASP1 | 0.951897 |
| ENST00000545531 | ARG1 | 0.975894637 | UCSC_5979_1768 | MASP1 | 0.957227 |
| ENST00000545531 | ARG1 | 0.967594778 | UCSC_8524_1151 | MASP1 | 0.960485 |
| lncRNAdb_60_500 | ARG1 | 0.968896511 | UCSC_11594_306 | MASP1 | 0.964407 |
| lncRNAdb_60_500 | ARG1 | 0.972540907 | CombinedLit_370_438 | MASP1 | 0.970022 |
| NRED_1255_416 | ARG1 | 0.957031254 | XLOC_012693 | MASP2 | 0.98577 |
| NRED_1255_416 | ARG1 | 0.959193248 | ENST00000417121 | MASP2 | 0.983559 |
| ENCODE_795_804 | ARG1 | 0.95123344 | ENST00000417851 | MASP2 | 0.975132 |
| ENCODE_1597_545 | ARG1 | 0.955176147 | ENST00000439524 | MASP2 | 0.987031 |
| ENCODE_2110_447 | ARG1 | 0.950522051 | ENST00000414604 | MASP2 | 0.981428 |
| ENCODE_2110_447 | ARG1 | 0.961254065 | ENST00000445096 | MASP2 | 0.981145 |
| RefSeq_532_3763 | ARG1 | 0.990299847 | ENST00000545531 | MASP2 | 0.973299 |
| RefSeq_532_3763 | ARG1 | 0.978725627 | lncRNAdb_60_500 | MASP2 | 0.95158 |
| RefSeq_1589_2127 | ARG1 | 0.960248417 | RefSeq_532_3763 | MASP2 | 0.985439 |
| RefSeq_1589_2127 | ARG1 | 0.967201236 | RefSeq_1589_2127 | MASP2 | 0.950354 |
| RefSeq_2351_1430 | ARG1 | 0.977433179 | RefSeq_2351_1430 | MASP2 | 0.972416 |
| RefSeq_2351_1430 | ARG1 | 0.966457788 | RefSeq_3051_754 | MASP2 | 0.95398 |
| UCSC_5722_1825 | ARG1 | 0.957500618 | UCSC_5722_1825 | MASP2 | 0.950032 |
| UCSC_5722_1825 | ARG1 | 0.960697022 | UCSC_6534_1640 | MASP2 | 0.966168 |
| UCSC_6534_1640 | ARG1 | 0.969122128 | UCSC_8524_1151 | MASP2 | 0.957433 |
| UCSC_6534_1640 | ARG1 | 0.96380954 | UCSC_11594_306 | MASP2 | 0.972458 |
| UCSC_8524_1151 | ARG1 | 0.967185045 | CombinedLit_370_438 | MASP2 | 0.988213 |
| UCSC_8524_1151 | ARG1 | 0.962564186 | XLOC_012693 | MAT1A | 0.978662 |
| UCSC_11594_306 | ARG1 | 0.972104966 | XLOC_003629 | MAT1A | 0.957489 |
| UCSC_11594_306 | ARG1 | 0.971467333 | XLOC_004515 | MAT1A | 0.975295 |
| CombinedLit_370_438 | ARG1 | 0.989824016 | ENST00000544018 | MAT1A | 0.958024 |
| CombinedLit_370_438 | ARG1 | 0.968966932 | ENST00000417121 | MAT1A | 0.968096 |
| ENST00000507582 | AS3MT | 0.957117163 | ENST00000417851 | MAT1A | 0.958117 |
| UCSC_5722_1825 | AS3MT | 0.951300793 | ENST00000439524 | MAT1A | 0.969015 |
| XLOC_012693 | ASGR1 | 0.980381101 | ENST00000414604 | MAT1A | 0.953635 |
| XLOC_003572 | ASGR1 | 0.954214849 | ENST00000445096 | MAT1A | 0.961693 |
| ENST00000417121 | ASGR1 | 0.972667386 | ENST00000545531 | MAT1A | 0.965378 |
| ENST00000417851 | ASGR1 | 0.964300749 | lncRNAdb_60_500 | MAT1A | 0.953482 |
| ENST00000439524 | ASGR1 | 0.976681195 | NRED_1255_416 | MAT1A | 0.953666 |
| ENST00000414604 | ASGR1 | 0.972757289 | ENCODE_1597_545 | MAT1A | 0.968332 |
| ENST00000445096 | ASGR1 | 0.976739162 | ENCODE_2110_447 | MAT1A | 0.950885 |
| ENST00000545531 | ASGR1 | 0.963120739 | RefSeq_532_3763 | MAT1A | 0.974383 |
| RefSeq_532_3763 | ASGR1 | 0.975538721 | RefSeq_1589_2127 | MAT1A | 0.964243 |
| RefSeq_2866_939 | ASGR1 | 0.955686273 | RefSeq_2351_1430 | MAT1A | 0.962545 |
| RefSeq_3051_754 | ASGR1 | 0.958585273 | UCSC_6534_1640 | MAT1A | 0.963899 |
| UCSC_11594_306 | ASGR1 | 0.965059545 | UCSC_8524_1151 | MAT1A | 0.963639 |
| CombinedLit_370_438 | ASGR1 | 0.982725627 | UCSC_11594_306 | MAT1A | 0.956731 |
| XLOC_012693 | ASGR2 | 0.95846546 | CombinedLit_370_438 | MAT1A | 0.961021 |
| XLOC_004515 | ASGR2 | 0.954970431 | XLOC_012693 | MBL2 | 0.991023 |
| ENST00000507582 | ASGR2 | 0.952307426 | ENST00000417121 | MBL2 | 0.978424 |
| lncRNAdb_60_500 | ASGR2 | 0.952275357 | ENST00000417851 | MBL2 | 0.970049 |
| RefSeq_532_3763 | ASGR2 | 0.965292607 | ENST00000439524 | MBL2 | 0.980482 |
| RefSeq_843_3032 | ASGR2 | 0.952049823 | ENST00000414604 | MBL2 | 0.975521 |
| RefSeq_1769_1968 | ASGR2 | 0.952856602 | ENST00000445096 | MBL2 | 0.980147 |
| UCSC_3328_2475 | ASGR2 | 0.953018708 | ENST00000545531 | MBL2 | 0.973534 |
| UCSC_5979_1768 | ASGR2 | 0.951488695 | lncRNAdb_60_500 | MBL2 | 0.973701 |
| UCSC_8524_1151 | ASGR2 | 0.951603515 | NRED_1255_416 | MBL2 | 0.960939 |
| UCSC_11594_306 | ASGR2 | 0.979904894 | ENCODE_2110_447 | MBL2 | 0.955146 |
| XLOC_012693 | ASPDH | 0.984045998 | RefSeq_532_3763 | MBL2 | 0.983354 |
| XLOC_003572 | ASPDH | 0.952643311 | RefSeq_1589_2127 | MBL2 | 0.950947 |
| ENST00000544018 | ASPDH | 0.950953776 | RefSeq_2351_1430 | MBL2 | 0.974407 |
| ENST00000417121 | ASPDH | 0.989528837 | UCSC_6534_1640 | MBL2 | 0.957934 |
| ENST00000417851 | ASPDH | 0.986212959 | UCSC_8524_1151 | MBL2 | 0.973158 |
| ENST00000439524 | ASPDH | 0.981215381 | UCSC_11594_306 | MBL2 | 0.971754 |
| ENST00000414604 | ASPDH | 0.970358804 | CombinedLit_370_438 | MBL2 | 0.990405 |
| ENST00000445096 | ASPDH | 0.985589054 | ENCODE_1156_661 | MED12L | 0.953714 |
| ENST00000545531 | ASPDH | 0.984255252 | UCSC_916_4223 | MMAB | 0.957428 |
| lncRNAdb_60_500 | ASPDH | 0.966725791 | UCSC_951_4161 | MMAB | 0.951541 |
| NRED_1255_416 | ASPDH | 0.968428723 | ENST00000507582 | MPPED1 | 0.961999 |
| ENCODE_2110_447 | ASPDH | 0.956947084 | UCSC_4189_2198 | MPPED1 | 0.954862 |
| ENCODE_2635_348 | ASPDH | 0.951930359 | UCSC_5722_1825 | MPPED1 | 0.961145 |
| RefSeq_532_3763 | ASPDH | 0.985766217 | XLOC_012693 | MST1 | 0.964263 |
| RefSeq_1589_2127 | ASPDH | 0.974883706 | XLOC_004515 | MST1 | 0.972929 |
| RefSeq_2351_1430 | ASPDH | 0.982168797 | ENST00000417121 | MST1 | 0.952793 |
| UCSC_5722_1825 | ASPDH | 0.951872831 | ENST00000439524 | MST1 | 0.979491 |
| UCSC_6534_1640 | ASPDH | 0.981200244 | ENST00000414604 | MST1 | 0.969928 |
| UCSC_8524_1151 | ASPDH | 0.964761215 | ENST00000445096 | MST1 | 0.958389 |
| UCSC_11594_306 | ASPDH | 0.962051901 | ENST00000545531 | MST1 | 0.953815 |
| CombinedLit_370_438 | ASPDH | 0.981672885 | ENCODE_1597_545 | MST1 | 0.960483 |
| XLOC_012693 | ATP2B2 | 0.971056299 | RefSeq_532_3763 | MST1 | 0.953129 |
| XLOC_012693 | ATP2B2 | 0.971209847 | RefSeq_1769_1968 | MST1 | 0.955006 |
| XLOC_002845 | ATP2B2 | 0.96164391 | UCSC_4189_2198 | MST1 | 0.957297 |
| XLOC_003572 | ATP2B2 | 0.95790767 | UCSC_6534_1640 | MST1 | 0.950048 |
| ENST00000507582 | ATP2B2 | 0.950550166 | CombinedLit_370_438 | MST1 | 0.954612 |
| ENST00000417121 | ATP2B2 | 0.980563681 | XLOC_002197 | MT1DP | 0.959162 |
| ENST00000417121 | ATP2B2 | 0.96803621 | UCSC_9337_977 | MT1L | 0.954101 |
| ENST00000417851 | ATP2B2 | 0.980318185 | UCSC_8286_1209 | MTHFD1 | 0.951058 |
| ENST00000417851 | ATP2B2 | 0.958918382 | XLOC_004515 | MTHFS | 0.951633 |
| ENST00000439524 | ATP2B2 | 0.968749863 | UCSC_4189_2198 | MTHFS | 0.957485 |
| ENST00000439524 | ATP2B2 | 0.969953714 | H-InvDB_14_1325 | MUC3B | 0.965375 |
| ENST00000414604 | ATP2B2 | 0.955877748 | H-InvDB_169_837 | MUC5AC | 0.991041 |
| ENST00000414604 | ATP2B2 | 0.964881902 | H-InvDB_837_492 | MUC5AC | 0.995079 |
| ENST00000445096 | ATP2B2 | 0.977995621 | H-InvDB_1632_341 | MUC5AC | 0.985683 |
| ENST00000445096 | ATP2B2 | 0.968392827 | XLOC_002845 | NAT8B | 0.95026 |
| ENST00000545531 | ATP2B2 | 0.970880752 | ENST00000417121 | NAT8B | 0.960384 |
| ENST00000545531 | ATP2B2 | 0.968861264 | ENST00000417851 | NAT8B | 0.951462 |
| lncRNAdb_60_500 | ATP2B2 | 0.969623062 | ENST00000439524 | NAT8B | 0.950609 |
| NRED_1255_416 | ATP2B2 | 0.973365059 | ENST00000445096 | NAT8B | 0.962358 |
| ENCODE_2018_465 | ATP2B2 | 0.951957257 | ENST00000545531 | NAT8B | 0.955901 |
| ENCODE_2110_447 | ATP2B2 | 0.971557311 | ENCODE_2110_447 | NAT8B | 0.963563 |
| ENCODE_2110_447 | ATP2B2 | 0.961586979 | UCSC_5722_1825 | NAT8B | 0.964655 |
| RefSeq_532_3763 | ATP2B2 | 0.959132158 | UCSC_10912_543 | NAT8B | 0.959508 |
| RefSeq_532_3763 | ATP2B2 | 0.956806942 | XLOC_008833 | NIPAL1 | 0.953568 |
| RefSeq_1589_2127 | ATP2B2 | 0.963258277 | XLOC_012693 | NR1I3 | 0.977517 |
| RefSeq_2351_1430 | ATP2B2 | 0.968079987 | XLOC_002019 | NR1I3 | 0.956914 |
| UCSC_5722_1825 | ATP2B2 | 0.963771074 | ENST00000507582 | NR1I3 | 0.950489 |
| UCSC_5722_1825 | ATP2B2 | 0.959589933 | ENST00000544018 | NR1I3 | 0.96356 |
| UCSC_6534_1640 | ATP2B2 | 0.956861102 | ENST00000417121 | NR1I3 | 0.977565 |
| UCSC_11594_306 | ATP2B2 | 0.966850395 | ENST00000417851 | NR1I3 | 0.973629 |
| UCSC_11594_306 | ATP2B2 | 0.952708862 | ENST00000417851 | NR1I3 | 0.950403 |
| CombinedLit_370_438 | ATP2B2 | 0.955473452 | ENST00000439524 | NR1I3 | 0.987963 |
| CombinedLit_370_438 | ATP2B2 | 0.967170707 | ENST00000414604 | NR1I3 | 0.982367 |
| UCSC_699_4625 | BCL2L15 | 0.980612055 | ENST00000445096 | NR1I3 | 0.974311 |
| XLOC_012693 | BHMT | 0.972319106 | ENST00000545531 | NR1I3 | 0.969143 |
| XLOC_004515 | BHMT | 0.961837608 | NRED_1255_416 | NR1I3 | 0.955718 |
| ENST00000507582 | BHMT | 0.964803569 | ENCODE_456_1286 | NR1I3 | 0.952459 |
| ENST00000538369 | BHMT | 0.95734914 | ENCODE_1597_545 | NR1I3 | 0.953025 |
| ENST00000544018 | BHMT | 0.954672915 | ENCODE_2110_447 | NR1I3 | 0.95784 |
| ENST00000376608 | BHMT | 0.955016748 | RefSeq_532_3763 | NR1I3 | 0.981713 |
| ENST00000417121 | BHMT | 0.957594759 | RefSeq_1589_2127 | NR1I3 | 0.962231 |
| ENST00000439524 | BHMT | 0.976784769 | RefSeq_2351_1430 | NR1I3 | 0.973802 |
| ENST00000414604 | BHMT | 0.970814745 | UCSC_5722_1825 | NR1I3 | 0.96356 |
| ENST00000445096 | BHMT | 0.956195304 | UCSC_6534_1640 | NR1I3 | 0.966595 |
| ENCODE_1597_545 | BHMT | 0.959588981 | UCSC_6534_1640 | NR1I3 | 0.952422 |
| ENCODE_2110_447 | BHMT | 0.952710689 | UCSC_11594_306 | NR1I3 | 0.968835 |
| RefSeq_532_3763 | BHMT | 0.98182354 | CombinedLit_370_438 | NR1I3 | 0.977648 |
| RefSeq_1589_2127 | BHMT | 0.954279592 | XLOC_012693 | NRAP | 0.952985 |
| RefSeq_1769_1968 | BHMT | 0.97380841 | ENST00000417121 | NRAP | 0.968299 |
| UCSC_5722_1825 | BHMT | 0.957941122 | ENST00000417851 | NRAP | 0.957331 |
| UCSC_8524_1151 | BHMT | 0.956096507 | ENST00000445096 | NRAP | 0.964588 |
| UCSC_11594_306 | BHMT | 0.983318625 | ENST00000545531 | NRAP | 0.957607 |
| CombinedLit_370_438 | BHMT | 0.973824593 | XLOC_002845 | NSUN6 | 0.957175 |
| ENST00000507582 | BHMT2 | 0.956978347 | ENST00000439524 | NSUN6 | 0.954252 |
| XLOC_002845 | BNIP3 | 0.958677395 | ENCODE_456_1286 | NSUN6 | 0.950574 |
| ENST00000417851 | BNIP3 | 0.95117261 | ENCODE_1597_545 | NSUN6 | 0.963336 |
| UCSC_5722_1825 | BNIP3 | 0.958462261 | UCSC_5722_1825 | NSUN6 | 0.968339 |
| UCSC_6534_1640 | BNIP3 | 0.952221913 | XLOC_012693 | OGDHL | 0.969649 |
| ENST00000544018 | C2 | 0.960182535 | XLOC_002845 | OGDHL | 0.958719 |
| ENST00000544018 | C2 | 0.959969843 | XLOC_003572 | OGDHL | 0.961837 |
| RefSeq_532_3763 | C2 | 0.955395841 | ENST00000507582 | OGDHL | 0.957547 |
| UCSC_11594_306 | C2 | 0.951810291 | ENST00000544018 | OGDHL | 0.978259 |
| ENST00000544018 | C4B | 0.95452762 | ENST00000417121 | OGDHL | 0.96589 |
| XLOC_012693 | C4BPA | 0.985011559 | ENST00000417851 | OGDHL | 0.964266 |
| ENST00000417121 | C4BPA | 0.964828821 | ENST00000439524 | OGDHL | 0.97309 |
| ENST00000439524 | C4BPA | 0.968662122 | ENST00000414604 | OGDHL | 0.971522 |
| ENST00000414604 | C4BPA | 0.963596372 | ENST00000445096 | OGDHL | 0.965135 |
| ENST00000445096 | C4BPA | 0.968062714 | ENST00000545531 | OGDHL | 0.961305 |
| ENST00000545531 | C4BPA | 0.966260065 | RefSeq_532_3763 | OGDHL | 0.97353 |
| lncRNAdb_60_500 | C4BPA | 0.954569831 | RefSeq_2351_1430 | OGDHL | 0.954752 |
| RefSeq_532_3763 | C4BPA | 0.967395873 | UCSC_5722_1825 | OGDHL | 0.969933 |
| RefSeq_2351_1430 | C4BPA | 0.961392307 | UCSC_6534_1640 | OGDHL | 0.961564 |
| UCSC_8524_1151 | C4BPA | 0.961433159 | CombinedLit_370_438 | OGDHL | 0.967135 |
| UCSC_11594_306 | C4BPA | 0.953169997 | ENST00000538369 | OIT3 | 0.950962 |
| CombinedLit_370_438 | C4BPA | 0.981955376 | ENST00000376608 | OIT3 | 0.951543 |
| XLOC_012693 | C5 | 0.97296471 | ENCODE_1931_477 | OIT3 | 0.952201 |
| XLOC_004515 | C5 | 0.952390629 | ENCODE_2461_388 | OIT3 | 0.955559 |
| ENST00000417121 | C5 | 0.952111428 | UCSC_11594_306 | OIT3 | 0.974284 |
| ENST00000439524 | C5 | 0.951037188 | UCSC_11757_206 | OR1E1 | 0.958877 |
| ENST00000445096 | C5 | 0.950431142 | XLOC_012693 | ORM1 | 0.975488 |
| lncRNAdb_60_500 | C5 | 0.962817796 | ENST00000417121 | ORM1 | 0.959364 |
| RefSeq_532_3763 | C5 | 0.975310414 | ENST00000439524 | ORM1 | 0.965474 |
| RefSeq_1589_2127 | C5 | 0.952031456 | ENST00000414604 | ORM1 | 0.95587 |
| UCSC_8524_1151 | C5 | 0.974312165 | ENST00000445096 | ORM1 | 0.953276 |
| UCSC_11594_306 | C5 | 0.965527806 | lncRNAdb_60_500 | ORM1 | 0.965955 |
| CombinedLit_370_438 | C5 | 0.967712632 | RefSeq_532_3763 | ORM1 | 0.981496 |
| NRED_1255_416 | C5orf33 | 0.95114287 | RefSeq_1589_2127 | ORM1 | 0.952149 |
| ENCODE_1597_545 | C5orf33 | 0.95911149 | RefSeq_2351_1430 | ORM1 | 0.963352 |
| ENCODE_2635_348 | C5orf33 | 0.962635611 | UCSC_8524_1151 | ORM1 | 0.980691 |
| RefSeq_1589_2127 | C5orf33 | 0.954566212 | UCSC_11594_306 | ORM1 | 0.971036 |
| ENST00000455853 | C6orf176 | 0.977545901 | CombinedLit_370_438 | ORM1 | 0.972646 |
| RefSeq_1922_1818 | C6orf176 | 0.954045431 | XLOC_012693 | ORM2 | 0.972092 |
| XLOC_012693 | C8A | 0.985247793 | XLOC_004515 | ORM2 | 0.95775 |
| ENST00000417121 | C8A | 0.958978558 | ENST00000417121 | ORM2 | 0.963016 |
| ENST00000439524 | C8A | 0.969218433 | ENST00000417851 | ORM2 | 0.952125 |
| ENST00000414604 | C8A | 0.962896842 | ENST00000439524 | ORM2 | 0.966604 |
| ENST00000445096 | C8A | 0.964558215 | ENST00000414604 | ORM2 | 0.953268 |
| ENST00000545531 | C8A | 0.954462972 | ENST00000445096 | ORM2 | 0.951385 |
| lncRNAdb_60_500 | C8A | 0.97110194 | lncRNAdb_60_500 | ORM2 | 0.957921 |
| ENCODE_2110_447 | C8A | 0.95125191 | NRED_1255_416 | ORM2 | 0.950109 |
| RefSeq_532_3763 | C8A | 0.985087186 | RefSeq_532_3763 | ORM2 | 0.981236 |
| RefSeq_1769_1968 | C8A | 0.958144261 | RefSeq_1589_2127 | ORM2 | 0.96203 |
| RefSeq_2351_1430 | C8A | 0.957141912 | RefSeq_2351_1430 | ORM2 | 0.96862 |
| UCSC_8524_1151 | C8A | 0.976432636 | UCSC_8524_1151 | ORM2 | 0.977037 |
| UCSC_11594_306 | C8A | 0.980340236 | UCSC_11594_306 | ORM2 | 0.972278 |
| CombinedLit_370_438 | C8A | 0.984267597 | CombinedLit_370_438 | ORM2 | 0.966096 |
| XLOC_012693 | C8B | 0.961518411 | ENCODE_430_1387 | PAH | 0.968176 |
| XLOC_004515 | C8B | 0.959973902 | ENCODE_791_805 | PAH | 0.965564 |
| ENST00000507582 | C8B | 0.956331123 | XLOC_012693 | PAQR9 | 0.95036 |
| ENST00000376608 | C8B | 0.954759577 | XLOC_012693 | PCK1 | 0.950723 |
| lncRNAdb_60_500 | C8B | 0.950989213 | RefSeq_2866_939 | PCK1 | 0.951313 |
| RefSeq_532_3763 | C8B | 0.959777526 | UCSC_3488_2413 | PCSK6 | 0.960803 |
| RefSeq_843_3032 | C8B | 0.959995315 | RefSeq_638_3498 | PDZK1 | 0.957815 |
| RefSeq_869_3001 | C8B | 0.952142553 | XLOC_002845 | PEBP1 | 0.956164 |
| RefSeq_1769_1968 | C8B | 0.973612644 | UCSC_6534_1640 | PECI | 0.950065 |
| UCSC_3328_2475 | C8B | 0.960442371 | RefSeq_843_3032 | PECR | 0.955261 |
| UCSC_8524_1151 | C8B | 0.958084646 | ENST00000538369 | PEMT | 0.953114 |
| UCSC_11594_306 | C8B | 0.973465429 | ENST00000544018 | PEMT | 0.951148 |
| CombinedLit_370_438 | C8B | 0.95260733 | ENCODE_2109_447 | PEMT | 0.955606 |
| asoverlaps_682_1255 | C8B | 0.952649966 | UCSC_6225_1713 | PEMT | 0.95117 |
| ENST00000417121 | C8G | 0.965953302 | ENCODE_2110_447 | PFKFB1 | 0.95972 |
| ENST00000417851 | C8G | 0.961447629 | RefSeq_1589_2127 | PFKFB1 | 0.952637 |
| ENST00000545531 | C8G | 0.952593561 | UCSC_5722_1825 | PFKFB1 | 0.959078 |
| UCSC_6534_1640 | C8G | 0.954981153 | RNAz_4014_229 | PGRMC1 | 0.952103 |
| UCSC_9299_985 | C8G | 0.952864563 | ENST00000538369 | PHYH | 0.954486 |
| UCSC_10912_543 | C8G | 0.957025608 | XLOC_012693 | PIPOX | 0.986724 |
| asoverlaps_653_1337 | C8G | 0.952631302 | XLOC_002845 | PIPOX | 0.955802 |
| XLOC_012693 | C9 | 0.973360869 | XLOC_003572 | PIPOX | 0.971827 |
| ENST00000376608 | C9 | 0.95176528 | XLOC_004515 | PIPOX | 0.958383 |
| ENST00000439524 | C9 | 0.960531274 | ENST00000507582 | PIPOX | 0.957896 |
| ENST00000414604 | C9 | 0.952007701 | ENST00000544018 | PIPOX | 0.963757 |
| lncRNAdb_60_500 | C9 | 0.962687737 | ENST00000417121 | PIPOX | 0.982252 |
| ENCODE_2110_447 | C9 | 0.951421507 | ENST00000417851 | PIPOX | 0.97853 |
| RefSeq_532_3763 | C9 | 0.978479342 | ENST00000439524 | PIPOX | 0.98011 |
| RefSeq_1769_1968 | C9 | 0.965856405 | ENST00000414604 | PIPOX | 0.972721 |
| UCSC_8524_1151 | C9 | 0.969836318 | ENST00000445096 | PIPOX | 0.983508 |
| UCSC_11594_306 | C9 | 0.986724101 | ENST00000545531 | PIPOX | 0.975841 |
| CombinedLit_370_438 | C9 | 0.968136012 | lncRNAdb_60_500 | PIPOX | 0.956063 |
| XLOC_012693 | CA5A | 0.957888313 | ENCODE_1597_545 | PIPOX | 0.959665 |
| ENST00000417121 | CA5A | 0.978855346 | ENCODE_2110_447 | PIPOX | 0.954346 |
| ENST00000417851 | CA5A | 0.981860516 | RefSeq_532_3763 | PIPOX | 0.979735 |
| ENST00000439524 | CA5A | 0.951727479 | RefSeq_2351_1430 | PIPOX | 0.964492 |
| ENST00000445096 | CA5A | 0.97152307 | RefSeq_2866_939 | PIPOX | 0.955126 |
| ENST00000545531 | CA5A | 0.981589501 | RefSeq_3051_754 | PIPOX | 0.951309 |
| RefSeq_2351_1430 | CA5A | 0.966445261 | UCSC_5722_1825 | PIPOX | 0.968476 |
| UCSC_6534_1640 | CA5A | 0.981979636 | UCSC_6534_1640 | PIPOX | 0.96895 |
| XLOC_012693 | CCL16 | 0.950819646 | UCSC_11594_306 | PIPOX | 0.964975 |
| ENST00000507582 | CCL16 | 0.972267041 | CombinedLit_370_438 | PIPOX | 0.980156 |
| ENST00000538369 | CCL16 | 0.968715214 | XLOC_012693 | PLG | 0.982905 |
| ENST00000376608 | CCL16 | 0.952535006 | ENST00000544018 | PLG | 0.958986 |
| ENST00000439524 | CCL16 | 0.960880952 | ENST00000417121 | PLG | 0.970641 |
| ENST00000414604 | CCL16 | 0.954661149 | ENST00000417851 | PLG | 0.95269 |
| ENCODE_795_804 | CCL16 | 0.953092177 | ENST00000439524 | PLG | 0.980914 |
| RefSeq_532_3763 | CCL16 | 0.960815928 | ENST00000414604 | PLG | 0.980225 |
| RefSeq_1769_1968 | CCL16 | 0.967459267 | ENST00000445096 | PLG | 0.971954 |
| UCSC_3328_2475 | CCL16 | 0.963508524 | ENST00000545531 | PLG | 0.971161 |
| UCSC_4189_2198 | CCL16 | 0.952399111 | RefSeq_532_3763 | PLG | 0.975581 |
| UCSC_5722_1825 | CCL16 | 0.951611098 | RefSeq_2351_1430 | PLG | 0.967199 |
| UCSC_11594_306 | CCL16 | 0.974267621 | RefSeq_3051_754 | PLG | 0.95789 |
| UCSC_5979_1768 | CFB | 0.950141572 | UCSC_5722_1825 | PLG | 0.952561 |
| ENST00000544018 | CFH | 0.950744388 | UCSC_6534_1640 | PLG | 0.950323 |
| XLOC_012693 | CFHR1 | 0.982318417 | UCSC_8524_1151 | PLG | 0.955416 |
| ENST00000439524 | CFHR1 | 0.963275525 | UCSC_11594_306 | PLG | 0.958297 |
| ENST00000414604 | CFHR1 | 0.959672658 | CombinedLit_370_438 | PLG | 0.987505 |
| ENST00000445096 | CFHR1 | 0.956523503 | ENST00000376608 | PLGLB1 | 0.952862 |
| lncRNAdb_60_500 | CFHR1 | 0.965647792 | RefSeq_1769_1968 | PLGLB1 | 0.952763 |
| RefSeq_532_3763 | CFHR1 | 0.980297468 | RefSeq_1769_1968 | PLGLB1 | 0.972493 |
| RefSeq_1769_1968 | CFHR1 | 0.959070629 | UCSC_3328_2475 | PLGLB1 | 0.959677 |
| UCSC_8524_1151 | CFHR1 | 0.976625 | UCSC_11594_306 | PLGLB1 | 0.961538 |
| UCSC_11594_306 | CFHR1 | 0.973494475 | UCSC_11594_306 | PLGLB1 | 0.957342 |
| CombinedLit_370_438 | CFHR1 | 0.981385721 | XLOC_012693 | PLIN5 | 0.962279 |
| XLOC_012693 | CFHR2 | 0.974909604 | XLOC_003572 | PLIN5 | 0.972414 |
| XLOC_003572 | CFHR2 | 0.95033754 | ENST00000445096 | PLIN5 | 0.955934 |
| ENST00000507582 | CFHR2 | 0.95880055 | ENST00000545531 | PLIN5 | 0.95559 |
| ENST00000439524 | CFHR2 | 0.95657564 | RefSeq_532_3763 | PLIN5 | 0.957288 |
| ENST00000414604 | CFHR2 | 0.950429082 | UCSC_3328_2475 | PLIN5 | 0.957518 |
| lncRNAdb_60_500 | CFHR2 | 0.955131169 | CombinedLit_370_438 | PLIN5 | 0.953098 |
| ENCODE_795_804 | CFHR2 | 0.951814575 | lncRNAdb_60_500 | PON1 | 0.952368 |
| RefSeq_532_3763 | CFHR2 | 0.976237106 | RefSeq_1769_1968 | PON1 | 0.9579 |
| RefSeq_1769_1968 | CFHR2 | 0.972127198 | UCSC_3328_2475 | PON1 | 0.952845 |
| UCSC_3328_2475 | CFHR2 | 0.966759313 | UCSC_8524_1151 | PON1 | 0.95259 |
| UCSC_8524_1151 | CFHR2 | 0.955391023 | UCSC_11594_306 | PON1 | 0.96118 |
| UCSC_11594_306 | CFHR2 | 0.986568445 | UCSC_8524_1151 | PON3 | 0.95636 |
| CombinedLit_370_438 | CFHR2 | 0.962967574 | UCSC_3488_2413 | POR | 0.95079 |
| ENST00000505626 | CFHR4 | 0.950406084 | XLOC_012693 | PROC | 0.979168 |
| RefSeq_843_3032 | CFHR4 | 0.950507123 | XLOC_012693 | PROC | 0.961326 |
| ENST00000505626 | CFHR5 | 0.952670241 | ENST00000417121 | PROC | 0.973185 |
| RefSeq_843_3032 | CFHR5 | 0.960497113 | ENST00000417121 | PROC | 0.96902 |
| RefSeq_869_3001 | CFHR5 | 0.953526994 | ENST00000417851 | PROC | 0.958565 |
| RefSeq_1769_1968 | CFHR5 | 0.952910779 | ENST00000417851 | PROC | 0.955495 |
| UCSC_3328_2475 | CFHR5 | 0.963794167 | ENST00000439524 | PROC | 0.984664 |
| XLOC_002845 | CIDEB | 0.953179592 | ENST00000439524 | PROC | 0.966085 |
| XLOC_012693 | CLEC1B | 0.98705464 | ENST00000414604 | PROC | 0.982779 |
| XLOC_003572 | CLEC1B | 0.958346035 | ENST00000414604 | PROC | 0.961643 |
| XLOC_004433 | CLEC1B | 0.964033101 | ENST00000445096 | PROC | 0.978946 |
| XLOC_004515 | CLEC1B | 0.951742646 | ENST00000445096 | PROC | 0.972318 |
| ENST00000544018 | CLEC1B | 0.955763008 | ENST00000545531 | PROC | 0.97947 |
| ENST00000417121 | CLEC1B | 0.972091845 | ENST00000545531 | PROC | 0.977411 |
| ENST00000417851 | CLEC1B | 0.96887813 | RefSeq_532_3763 | PROC | 0.961832 |
| ENST00000417851 | CLEC1B | 0.95046975 | RefSeq_2351_1430 | PROC | 0.959063 |
| ENST00000439524 | CLEC1B | 0.970334856 | RefSeq_3051_754 | PROC | 0.950437 |
| ENST00000414604 | CLEC1B | 0.962789528 | RefSeq_3051_754 | PROC | 0.950663 |
| ENST00000445096 | CLEC1B | 0.973000181 | UCSC_6534_1640 | PROC | 0.96492 |
| ENST00000545531 | CLEC1B | 0.968844145 | UCSC_6534_1640 | PROC | 0.956367 |
| lncRNAdb_60_500 | CLEC1B | 0.971189747 | CombinedLit_370_438 | PROC | 0.977127 |
| NRED_1255_416 | CLEC1B | 0.959033261 | CombinedLit_370_438 | PROC | 0.957522 |
| ENCODE_1597_545 | CLEC1B | 0.950295838 | XLOC_012693 | PRODH2 | 0.974625 |
| ENCODE_2110_447 | CLEC1B | 0.967371172 | ENST00000417121 | PRODH2 | 0.987573 |
| RefSeq_532_3763 | CLEC1B | 0.980856521 | ENST00000417851 | PRODH2 | 0.978956 |
| RefSeq_2351_1430 | CLEC1B | 0.969950426 | ENST00000439524 | PRODH2 | 0.982483 |
| RefSeq_2351_1430 | CLEC1B | 0.950252265 | ENST00000414604 | PRODH2 | 0.972886 |
| UCSC_5210_1945 | CLEC1B | 0.95157968 | ENST00000445096 | PRODH2 | 0.983656 |
| UCSC_5722_1825 | CLEC1B | 0.951456044 | ENST00000545531 | PRODH2 | 0.985152 |
| UCSC_6534_1640 | CLEC1B | 0.96244512 | NRED_1255_416 | PRODH2 | 0.950261 |
| UCSC_6534_1640 | CLEC1B | 0.953651051 | ENCODE_1597_545 | PRODH2 | 0.953789 |
| UCSC_8524_1151 | CLEC1B | 0.959522491 | ENCODE_2635_348 | PRODH2 | 0.958107 |
| UCSC_10583_636 | CLEC1B | 0.964658629 | RefSeq_532_3763 | PRODH2 | 0.96168 |
| UCSC_11594_306 | CLEC1B | 0.964933385 | RefSeq_1589_2127 | PRODH2 | 0.959384 |
| CombinedLit_370_438 | CLEC1B | 0.975170803 | RefSeq_2351_1430 | PRODH2 | 0.966443 |
| XLOC_002019 | CNDP1 | 0.955017095 | UCSC_5722_1825 | PRODH2 | 0.954885 |
| UCSC_6534_1640 | CNDP1 | 0.956526561 | UCSC_6534_1640 | PRODH2 | 0.977155 |
| XLOC_001387 | COLEC10 | 0.953618681 | CombinedLit_370_438 | PRODH2 | 0.966678 |
| XLOC_004515 | COLEC10 | 0.951195735 | XLOC_012693 | PROZ | 0.981538 |
| ENST00000439524 | COLEC10 | 0.953155639 | ENST00000544018 | PROZ | 0.965582 |
| NRED_1255_416 | COLEC10 | 0.960106364 | ENST00000417121 | PROZ | 0.973351 |
| RefSeq_1589_2127 | COLEC10 | 0.956850558 | ENST00000417851 | PROZ | 0.963751 |
| RefSeq_2351_1430 | COLEC10 | 0.950998209 | ENST00000439524 | PROZ | 0.980581 |
| UCSC_5722_1825 | COLEC10 | 0.964352381 | ENST00000414604 | PROZ | 0.980007 |
| XLOC_012693 | CPB2 | 0.98439986 | ENST00000445096 | PROZ | 0.97639 |
| XLOC_003572 | CPB2 | 0.953476823 | ENST00000545531 | PROZ | 0.975292 |
| ENST00000417121 | CPB2 | 0.964114958 | RefSeq_532_3763 | PROZ | 0.97167 |
| ENST00000417851 | CPB2 | 0.959391492 | RefSeq_2351_1430 | PROZ | 0.96998 |
| ENST00000439524 | CPB2 | 0.96463692 | RefSeq_2866_939 | PROZ | 0.956517 |
| ENST00000414604 | CPB2 | 0.956295888 | RefSeq_3051_754 | PROZ | 0.963258 |
| ENST00000445096 | CPB2 | 0.968605846 | UCSC_5722_1825 | PROZ | 0.952595 |
| ENST00000545531 | CPB2 | 0.960103409 | UCSC_6534_1640 | PROZ | 0.964658 |
| lncRNAdb_60_500 | CPB2 | 0.967297963 | CombinedLit_370_438 | PROZ | 0.983339 |
| NRED_1255_416 | CPB2 | 0.951466757 | XLOC_003572 | QPRT | 0.951949 |
| RefSeq_532_3763 | CPB2 | 0.981665834 | ENST00000544018 | QPRT | 0.961121 |
| RefSeq_2351_1430 | CPB2 | 0.967909491 | XLOC_012847 | RAB27B | 0.965026 |
| UCSC_6534_1640 | CPB2 | 0.955234573 | RefSeq_2565_1213 | RASSF3 | -0.96282 |
| UCSC_8524_1151 | CPB2 | 0.970526233 | ENCODE_2635_348 | RBKS | 0.95055 |
| UCSC_11594_306 | CPB2 | 0.955111743 | RefSeq_1589_2127 | RBKS | 0.953006 |
| CombinedLit_370_438 | CPB2 | 0.980079444 | XLOC_012693 | RBP4 | 0.971549 |
| XLOC_004515 | CPN1 | 0.952918676 | ENST00000507582 | RBP4 | 0.952535 |
| ENST00000538369 | CPN1 | 0.970188408 | ENST00000417121 | RBP4 | 0.951749 |
| ENST00000452481 | CPN1 | 0.955269411 | ENST00000439524 | RBP4 | 0.974366 |
| ENST00000439524 | CPN1 | 0.955586978 | ENST00000414604 | RBP4 | 0.97479 |
| RefSeq_1769_1968 | CPN1 | 0.950068023 | ENST00000445096 | RBP4 | 0.958549 |
| UCSC_4189_2198 | CPN1 | 0.963634475 | RefSeq_532_3763 | RBP4 | 0.971778 |
| UCSC_5210_1945 | CPN1 | 0.962697091 | RefSeq_1769_1968 | RBP4 | 0.962818 |
| UCSC_11594_306 | CPN1 | 0.960503364 | UCSC_11594_306 | RBP4 | 0.975421 |
| XLOC_012693 | CPN2 | 0.957475661 | CombinedLit_370_438 | RBP4 | 0.975437 |
| XLOC_002845 | CPN2 | 0.955749804 | ENCODE_800_800 | RDX | 0.950787 |
| XLOC_003572 | CPN2 | 0.950992454 | UCSC_6534_1640 | REEP6 | 0.956383 |
| ENST00000507582 | CPN2 | 0.967271636 | XLOC_001387 | RTP3 | 0.962899 |
| ENST00000439524 | CPN2 | 0.966812626 | ENST00000507582 | RTP3 | 0.970781 |
| ENST00000414604 | CPN2 | 0.960867396 | ENST00000439524 | RTP3 | 0.958864 |
| ENCODE_2110_447 | CPN2 | 0.971859269 | RefSeq_532_3763 | RTP3 | 0.950204 |
| RefSeq_532_3763 | CPN2 | 0.956777503 | UCSC_4189_2198 | RTP3 | 0.952911 |
| RefSeq_1589_2127 | CPN2 | 0.957958118 | UCSC_5722_1825 | RTP3 | 0.978413 |
| UCSC_5722_1825 | CPN2 | 0.977822279 | UCSC_5979_1768 | RTP3 | 0.951355 |
| UCSC_11594_306 | CPN2 | 0.967171034 | UCSC_11594_306 | RTP3 | 0.966984 |
| ENST00000439524 | CPS1 | 0.953626758 | XLOC_012693 | SAA4 | 0.979849 |
| RefSeq_954_2865 | CRAT | 0.988371574 | XLOC_003572 | SAA4 | 0.952335 |
| ENST00000507582 | CRHBP | 0.975912323 | ENST00000417121 | SAA4 | 0.956015 |
| ENST00000538369 | CRHBP | 0.969140313 | ENST00000439524 | SAA4 | 0.966048 |
| ENST00000376608 | CRHBP | 0.961968975 | ENST00000414604 | SAA4 | 0.964022 |
| UCSC_5722_1825 | CRHBP | 0.954845593 | ENST00000445096 | SAA4 | 0.960091 |
| XLOC_001387 | CRYAA | 0.955057909 | ENST00000545531 | SAA4 | 0.952195 |
| UCSC_5722_1825 | CRYAA | 0.975508814 | lncRNAdb_60_500 | SAA4 | 0.962812 |
| XLOC_012693 | CTH | 0.952949779 | RefSeq_532_3763 | SAA4 | 0.988813 |
| XLOC_004515 | CTH | 0.953364742 | RefSeq_2351_1430 | SAA4 | 0.955519 |
| ENST00000417121 | CTH | 0.954093274 | UCSC_8524_1151 | SAA4 | 0.969155 |
| ENST00000417851 | CTH | 0.954472327 | UCSC_11594_306 | SAA4 | 0.969773 |
| ENST00000439524 | CTH | 0.956259161 | CombinedLit_370_438 | SAA4 | 0.977165 |
| ENST00000445096 | CTH | 0.950642452 | XLOC_004398 | SARDH | 0.958489 |
| ENCODE_1597_545 | CTH | 0.954571102 | ENST00000507582 | SARDH | 0.959585 |
| UCSC_5722_1825 | CTH | 0.950767203 | ENST00000544018 | SARDH | 0.956708 |
| UCSC_6534_1640 | CTH | 0.960812634 | ENST00000376608 | SARDH | 0.95028 |
| RefSeq_1573_2136 | CXCR2P1 | 0.990353072 | ENST00000417851 | SARDH | 0.956848 |
| XLOC_004515 | CYB5A | 0.954519055 | UCSC_6534_1640 | SARDH | 0.956517 |
| ENST00000538369 | CYB5A | 0.962904406 | XLOC_001387 | SCP2 | 0.971828 |
| ENST00000452481 | CYB5A | 0.959110309 | XLOC_002845 | SCP2 | 0.969588 |
| ENST00000417851 | CYB5A | 0.950111265 | XLOC_004515 | SCP2 | 0.951748 |
| ENCODE_1597_545 | CYB5A | 0.950806858 | ENST00000507582 | SCP2 | 0.958468 |
| ENCODE_2635_348 | CYB5A | 0.956615542 | ENST00000544018 | SCP2 | 0.963404 |
| RefSeq_1589_2127 | CYB5A | 0.95022032 | ENST00000417121 | SCP2 | 0.952673 |
| UCSC_4189_2198 | CYB5A | 0.959717889 | ENST00000417851 | SCP2 | 0.955602 |
| UCSC_6534_1640 | CYB5A | 0.960600205 | ENST00000439524 | SCP2 | 0.956586 |
| UCSC_10539_647 | CYB5A | 0.950856937 | NRED_1255_416 | SCP2 | 0.955216 |
| XLOC_002845 | CYP27A1 | 0.971789872 | ENCODE_456_1286 | SCP2 | 0.952278 |
| ENCODE_456_1286 | CYP27A1 | 0.955286157 | ENCODE_1597_545 | SCP2 | 0.961168 |
| UCSC_5722_1825 | CYP27A1 | 0.959394811 | ENCODE_2110_447 | SCP2 | 0.950793 |
| XLOC_012693 | CYP2A13 | 0.953055734 | ENCODE_2635_348 | SCP2 | 0.965546 |
| XLOC_003572 | CYP2A13 | 0.957411621 | RefSeq_1589_2127 | SCP2 | 0.964429 |
| ENST00000233139 | CYP2A13 | 0.954773678 | UCSC_5722_1825 | SCP2 | 0.979172 |
| ENST00000417121 | CYP2A13 | 0.962712428 | UCSC_6534_1640 | SCP2 | 0.96731 |
| ENST00000417121 | CYP2A13 | 0.950259275 | UCSC_10912_543 | SCP2 | 0.955907 |
| ENST00000417851 | CYP2A13 | 0.952165786 | XLOC_012693 | SERPINA10 | 0.979566 |
| ENST00000439524 | CYP2A13 | 0.952891112 | XLOC_004515 | SERPINA10 | 0.970809 |
| ENST00000445096 | CYP2A13 | 0.961002651 | ENST00000507582 | SERPINA10 | 0.95629 |
| ENST00000545531 | CYP2A13 | 0.967267582 | ENST00000538369 | SERPINA10 | 0.957934 |
| ENCODE_1597_545 | CYP2A13 | 0.95640103 | ENST00000376608 | SERPINA10 | 0.956029 |
| ENCODE_2635_348 | CYP2A13 | 0.950057596 | ENST00000417121 | SERPINA10 | 0.964963 |
| ENCODE_2635_348 | CYP2A13 | 0.96798147 | ENST00000417851 | SERPINA10 | 0.957385 |
| RefSeq_1589_2127 | CYP2A13 | 0.951737241 | ENST00000439524 | SERPINA10 | 0.972921 |
| RefSeq_1589_2127 | CYP2A13 | 0.957050422 | ENST00000414604 | SERPINA10 | 0.959966 |
| UCSC_10912_543 | CYP2A13 | 0.959597796 | ENST00000445096 | SERPINA10 | 0.960015 |
| XLOC_012693 | CYP2A7 | 0.971073149 | ENST00000545531 | SERPINA10 | 0.951651 |
| XLOC_003572 | CYP2A7 | 0.968424472 | lncRNAdb_60_500 | SERPINA10 | 0.96554 |
| ENST00000507582 | CYP2A7 | 0.963479868 | NRED_1255_416 | SERPINA10 | 0.956195 |
| ENST00000417121 | CYP2A7 | 0.974188965 | ENCODE_1597_545 | SERPINA10 | 0.957156 |
| ENST00000417851 | CYP2A7 | 0.967359782 | ENCODE_2110_447 | SERPINA10 | 0.963555 |
| ENST00000439524 | CYP2A7 | 0.974766249 | RefSeq_532_3763 | SERPINA10 | 0.980253 |
| ENST00000414604 | CYP2A7 | 0.967338851 | RefSeq_1589_2127 | SERPINA10 | 0.965712 |
| ENST00000445096 | CYP2A7 | 0.976609401 | RefSeq_1769_1968 | SERPINA10 | 0.954858 |
| ENST00000545531 | CYP2A7 | 0.971321691 | RefSeq_2351_1430 | SERPINA10 | 0.95618 |
| NRED_1255_416 | CYP2A7 | 0.958650785 | UCSC_4189_2198 | SERPINA10 | 0.957444 |
| ENCODE_1597_545 | CYP2A7 | 0.962940023 | UCSC_5722_1825 | SERPINA10 | 0.964573 |
| ENCODE_2110_447 | CYP2A7 | 0.963160912 | UCSC_6534_1640 | SERPINA10 | 0.955244 |
| ENCODE_2635_348 | CYP2A7 | 0.965499844 | UCSC_8524_1151 | SERPINA10 | 0.965726 |
| RefSeq_532_3763 | CYP2A7 | 0.963751191 | UCSC_11594_306 | SERPINA10 | 0.981152 |
| RefSeq_1589_2127 | CYP2A7 | 0.960990547 | CombinedLit_370_438 | SERPINA10 | 0.964879 |
| RefSeq_2351_1430 | CYP2A7 | 0.953926665 | UCSC_5979_1768 | SERPINA4 | 0.957631 |
| UCSC_5722_1825 | CYP2A7 | 0.953552828 | XLOC_012693 | SERPINA7 | 0.963333 |
| UCSC_6534_1640 | CYP2A7 | 0.955349442 | ENST00000507582 | SERPINA7 | 0.954989 |
| UCSC_11594_306 | CYP2A7 | 0.958087797 | ENST00000439524 | SERPINA7 | 0.95809 |
| CombinedLit_370_438 | CYP2A7 | 0.973229283 | ENST00000414604 | SERPINA7 | 0.950582 |
| XLOC_012693 | CYP2A7P1 | 0.961514717 | RefSeq_532_3763 | SERPINA7 | 0.963768 |
| XLOC_001387 | CYP2A7P1 | 0.957405576 | RefSeq_843_3032 | SERPINA7 | 0.950738 |
| XLOC_003572 | CYP2A7P1 | 0.960827798 | RefSeq_1769_1968 | SERPINA7 | 0.969337 |
| XLOC_004515 | CYP2A7P1 | 0.952485288 | UCSC_3328_2475 | SERPINA7 | 0.954335 |
| ENST00000507582 | CYP2A7P1 | 0.972828088 | UCSC_11594_306 | SERPINA7 | 0.984635 |
| ENST00000417121 | CYP2A7P1 | 0.964474441 | CombinedLit_370_438 | SERPINA7 | 0.953595 |
| ENST00000417851 | CYP2A7P1 | 0.956633806 | XLOC_012693 | SERPINC1 | 0.982145 |
| ENST00000439524 | CYP2A7P1 | 0.972592138 | XLOC_003572 | SERPINC1 | 0.958036 |
| ENST00000414604 | CYP2A7P1 | 0.964014458 | ENST00000507582 | SERPINC1 | 0.969786 |
| ENST00000445096 | CYP2A7P1 | 0.964410645 | ENST00000417121 | SERPINC1 | 0.957071 |
| ENST00000545531 | CYP2A7P1 | 0.962772317 | ENST00000439524 | SERPINC1 | 0.977798 |
| NRED_1255_416 | CYP2A7P1 | 0.951124658 | ENST00000414604 | SERPINC1 | 0.975115 |
| ENCODE_1597_545 | CYP2A7P1 | 0.965564436 | ENST00000445096 | SERPINC1 | 0.965461 |
| ENCODE_2110_447 | CYP2A7P1 | 0.962934726 | ENST00000545531 | SERPINC1 | 0.950971 |
| ENCODE_2635_348 | CYP2A7P1 | 0.961153579 | lncRNAdb_60_500 | SERPINC1 | 0.958483 |
| RefSeq_532_3763 | CYP2A7P1 | 0.954354368 | ENCODE_795_804 | SERPINC1 | 0.951005 |
| RefSeq_1589_2127 | CYP2A7P1 | 0.955907347 | ENCODE_2110_447 | SERPINC1 | 0.962711 |
| RefSeq_2351_1430 | CYP2A7P1 | 0.951063653 | RefSeq_532_3763 | SERPINC1 | 0.983296 |
| UCSC_5722_1825 | CYP2A7P1 | 0.957742778 | RefSeq_1769_1968 | SERPINC1 | 0.973485 |
| UCSC_10912_543 | CYP2A7P1 | 0.958064906 | UCSC_3328_2475 | SERPINC1 | 0.958845 |
| UCSC_11594_306 | CYP2A7P1 | 0.951842711 | UCSC_5722_1825 | SERPINC1 | 0.953373 |
| CombinedLit_370_438 | CYP2A7P1 | 0.960497438 | UCSC_8524_1151 | SERPINC1 | 0.957416 |
| RefSeq_925_2910 | CYP2B6 | 0.985264259 | UCSC_11594_306 | SERPINC1 | 0.99282 |
| XLOC_012693 | CYP2C8 | 0.964176665 | CombinedLit_370_438 | SERPINC1 | 0.981651 |
| ENST00000417121 | CYP2C8 | 0.95516709 | XLOC_012693 | SERPIND1 | 0.987292 |
| ENST00000439524 | CYP2C8 | 0.961161766 | XLOC_003572 | SERPIND1 | 0.953348 |
| ENST00000414604 | CYP2C8 | 0.955048559 | ENST00000507582 | SERPIND1 | 0.956874 |
| ENST00000445096 | CYP2C8 | 0.959278164 | ENST00000417121 | SERPIND1 | 0.975922 |
| ENST00000545531 | CYP2C8 | 0.959272444 | ENST00000417851 | SERPIND1 | 0.962495 |
| NRED_1255_416 | CYP2C8 | 0.959700781 | ENST00000439524 | SERPIND1 | 0.985561 |
| RefSeq_2351_1430 | CYP2C8 | 0.953190522 | ENST00000414604 | SERPIND1 | 0.985428 |
| UCSC_6534_1640 | CYP2C8 | 0.965352942 | ENST00000445096 | SERPIND1 | 0.981552 |
| CombinedLit_370_438 | CYP2C8 | 0.958530924 | ENST00000545531 | SERPIND1 | 0.969814 |
| XLOC_012693 | CYP2E1 | 0.97032188 | RefSeq_532_3763 | SERPIND1 | 0.9809 |
| ENST00000544018 | CYP2E1 | 0.950369706 | RefSeq_1769_1968 | SERPIND1 | 0.958641 |
| ENST00000439524 | CYP2E1 | 0.962057554 | RefSeq_2351_1430 | SERPIND1 | 0.952012 |
| ENST00000414604 | CYP2E1 | 0.962096985 | RefSeq_2866_939 | SERPIND1 | 0.955357 |
| RefSeq_532_3763 | CYP2E1 | 0.97262547 | RefSeq_3051_754 | SERPIND1 | 0.954722 |
| RefSeq_2351_1430 | CYP2E1 | 0.958429986 | RefSeq_3175_631 | SERPIND1 | 0.955431 |
| UCSC_8524_1151 | CYP2E1 | 0.952348782 | UCSC_3328_2475 | SERPIND1 | 0.950975 |
| CombinedLit_370_438 | CYP2E1 | 0.969969314 | UCSC_5722_1825 | SERPIND1 | 0.950416 |
| RefSeq_2242_1543 | CYP2J2 | 0.951711948 | UCSC_6534_1640 | SERPIND1 | 0.951262 |
| ENCODE_2018_465 | CYP3A43 | 0.953071277 | UCSC_11594_306 | SERPIND1 | 0.975773 |
| ENCODE_2110_447 | CYP3A43 | 0.96045576 | CombinedLit_370_438 | SERPIND1 | 0.992452 |
| UCSC_10912_543 | CYP3A43 | 0.954867573 | ENST00000447298 | SERPINF2 | 0.989208 |
| H-InvDB_178_818 | CYP3A43 | 0.964082063 | XLOC_012693 | SERPINF2 | 0.965899 |
| XLOC_012693 | CYP4A11 | 0.987524517 | ENST00000544018 | SERPINF2 | 0.970688 |
| XLOC_001387 | CYP4A11 | 0.951896147 | ENST00000417121 | SERPINF2 | 0.956657 |
| XLOC_003572 | CYP4A11 | 0.953233432 | ENST00000439524 | SERPINF2 | 0.969534 |
| ENST00000505626 | CYP4A11 | 0.969439184 | ENST00000414604 | SERPINF2 | 0.972147 |
| ENST00000507582 | CYP4A11 | 0.9739229 | ENST00000445096 | SERPINF2 | 0.954619 |
| ENST00000538369 | CYP4A11 | 0.957316661 | ENST00000545531 | SERPINF2 | 0.957336 |
| ENST00000417121 | CYP4A11 | 0.977989548 | RefSeq_532_3763 | SERPINF2 | 0.977454 |
| ENST00000417851 | CYP4A11 | 0.977515164 | RefSeq_2351_1430 | SERPINF2 | 0.960376 |
| ENST00000439524 | CYP4A11 | 0.980258534 | UCSC_11594_306 | SERPINF2 | 0.950522 |
| ENST00000414604 | CYP4A11 | 0.976110154 | CombinedLit_370_438 | SERPINF2 | 0.974514 |
| ENST00000445096 | CYP4A11 | 0.981884021 | ENST00000447298 | SERPING1 | 0.980488 |
| ENST00000545531 | CYP4A11 | 0.971924504 | XLOC_009974 | SHMT1 | 0.95302 |
| lncRNAdb_60_500 | CYP4A11 | 0.964293905 | XLOC_012693 | SLC10A1 | 0.97879 |
| NRED_1255_416 | CYP4A11 | 0.968611044 | XLOC_001387 | SLC10A1 | 0.963888 |
| RefSeq_532_3763 | CYP4A11 | 0.981778304 | XLOC_002845 | SLC10A1 | 0.959878 |
| RefSeq_843_3032 | CYP4A11 | 0.973948262 | XLOC_003572 | SLC10A1 | 0.971683 |
| RefSeq_869_3001 | CYP4A11 | 0.968694534 | XLOC_004515 | SLC10A1 | 0.953936 |
| RefSeq_1589_2127 | CYP4A11 | 0.957944588 | ENST00000507582 | SLC10A1 | 0.982276 |
| RefSeq_1769_1968 | CYP4A11 | 0.974509824 | ENST00000544018 | SLC10A1 | 0.959024 |
| RefSeq_2351_1430 | CYP4A11 | 0.966450576 | ENST00000376608 | SLC10A1 | 0.950268 |
| UCSC_3328_2475 | CYP4A11 | 0.977275276 | ENST00000417121 | SLC10A1 | 0.969495 |
| UCSC_4189_2198 | CYP4A11 | 0.951427075 | ENST00000417851 | SLC10A1 | 0.963043 |
| UCSC_6534_1640 | CYP4A11 | 0.971555436 | ENST00000439524 | SLC10A1 | 0.980959 |
| UCSC_8524_1151 | CYP4A11 | 0.953034426 | ENST00000414604 | SLC10A1 | 0.976299 |
| UCSC_11594_306 | CYP4A11 | 0.958732293 | ENST00000445096 | SLC10A1 | 0.973769 |
| UCSC_11594_306 | CYP4A11 | 0.9596538 | ENST00000545531 | SLC10A1 | 0.965385 |
| CombinedLit_370_438 | CYP4A11 | 0.981677687 | lncRNAdb_60_500 | SLC10A1 | 0.954951 |
| XLOC_012693 | CYP4A22 | 0.981082144 | ENCODE_1597_545 | SLC10A1 | 0.953839 |
| XLOC_003572 | CYP4A22 | 0.954055077 | ENCODE_2110_447 | SLC10A1 | 0.97881 |
| XLOC_004515 | CYP4A22 | 0.961744466 | RefSeq_532_3763 | SLC10A1 | 0.972903 |
| ENST00000507582 | CYP4A22 | 0.951525699 | RefSeq_1589_2127 | SLC10A1 | 0.950248 |
| ENST00000544018 | CYP4A22 | 0.969522295 | RefSeq_1769_1968 | SLC10A1 | 0.956648 |
| ENST00000417121 | CYP4A22 | 0.982044542 | RefSeq_2351_1430 | SLC10A1 | 0.953469 |
| ENST00000417851 | CYP4A22 | 0.978808682 | UCSC_3328_2475 | SLC10A1 | 0.956774 |
| ENST00000439524 | CYP4A22 | 0.985019589 | UCSC_5722_1825 | SLC10A1 | 0.978918 |
| ENST00000414604 | CYP4A22 | 0.976576522 | UCSC_6534_1640 | SLC10A1 | 0.953766 |
| ENST00000445096 | CYP4A22 | 0.977092081 | UCSC_10912_543 | SLC10A1 | 0.951275 |
| ENST00000545531 | CYP4A22 | 0.977084686 | UCSC_11594_306 | SLC10A1 | 0.979278 |
| lncRNAdb_60_500 | CYP4A22 | 0.951803981 | CombinedLit_370_438 | SLC10A1 | 0.973464 |
| NRED_1255_416 | CYP4A22 | 0.971923178 | XLOC_012693 | SLC13A5 | 0.960849 |
| ENCODE_981_731 | CYP4A22 | 0.950613044 | XLOC_004515 | SLC13A5 | 0.978261 |
| ENCODE_1597_545 | CYP4A22 | 0.966429257 | ENST00000439524 | SLC13A5 | 0.961025 |
| ENCODE_2110_447 | CYP4A22 | 0.958342534 | ENCODE_1597_545 | SLC13A5 | 0.950501 |
| ENCODE_2635_348 | CYP4A22 | 0.96059519 | RefSeq_532_3763 | SLC13A5 | 0.961881 |
| RefSeq_532_3763 | CYP4A22 | 0.982300871 | UCSC_4189_2198 | SLC13A5 | 0.966463 |
| RefSeq_1589_2127 | CYP4A22 | 0.973506099 | UCSC_8524_1151 | SLC13A5 | 0.955462 |
| RefSeq_2351_1430 | CYP4A22 | 0.979098151 | XLOC_001387 | SLC17A1 | 0.966933 |
| UCSC_5722_1825 | CYP4A22 | 0.969292874 | XLOC_002845 | SLC17A1 | 0.973078 |
| UCSC_6534_1640 | CYP4A22 | 0.980374204 | XLOC_004515 | SLC17A1 | 0.962579 |
| UCSC_8524_1151 | CYP4A22 | 0.959413267 | ENST00000507582 | SLC17A1 | 0.964699 |
| UCSC_11594_306 | CYP4A22 | 0.95750891 | ENST00000538369 | SLC17A1 | 0.953994 |
| CombinedLit_370_438 | CYP4A22 | 0.974617679 | UCSC_4189_2198 | SLC17A1 | 0.963406 |
| RefSeq_2242_1543 | CYP4F2 | 0.957947488 | UCSC_5722_1825 | SLC17A1 | 0.981412 |
| RefSeq_2242_1543 | CYP4F2 | 0.981686878 | UCSC_10912_543 | SLC17A1 | 0.952386 |
| RefSeq_2242_1543 | CYP4F8 | 0.987846646 | XLOC_012693 | SLC17A2 | 0.963451 |
| XLOC_012693 | CYP8B1 | 0.951306949 | XLOC_001387 | SLC17A2 | 0.953199 |
| XLOC_001387 | CYP8B1 | 0.957964798 | XLOC_002845 | SLC17A2 | 0.963534 |
| ENST00000507582 | CYP8B1 | 0.979665748 | XLOC_003572 | SLC17A2 | 0.952326 |
| ENST00000538369 | CYP8B1 | 0.964138834 | XLOC_004515 | SLC17A2 | 0.972891 |
| ENST00000376608 | CYP8B1 | 0.951613677 | ENST00000507582 | SLC17A2 | 0.978889 |
| ENST00000439524 | CYP8B1 | 0.959476491 | ENST00000507582 | SLC17A2 | 0.958889 |
| ENST00000414604 | CYP8B1 | 0.958662746 | ENST00000538369 | SLC17A2 | 0.957946 |
| RefSeq_1769_1968 | CYP8B1 | 0.961621764 | ENST00000538369 | SLC17A2 | 0.959398 |
| UCSC_3328_2475 | CYP8B1 | 0.965094084 | ENST00000544018 | SLC17A2 | 0.967093 |
| UCSC_4189_2198 | CYP8B1 | 0.951785378 | ENST00000376608 | SLC17A2 | 0.96599 |
| UCSC_5722_1825 | CYP8B1 | 0.967740214 | ENST00000439524 | SLC17A2 | 0.96646 |
| UCSC_11594_306 | CYP8B1 | 0.969392599 | ENST00000414604 | SLC17A2 | 0.958091 |
| XLOC_012693 | DBH | 0.950445958 | ENCODE_795_804 | SLC17A2 | 0.953518 |
| ENST00000417121 | DBH | 0.956653638 | ENCODE_1597_545 | SLC17A2 | 0.967701 |
| ENST00000417851 | DBH | 0.95489207 | RefSeq_532_3763 | SLC17A2 | 0.952942 |
| ENST00000439524 | DBH | 0.951951663 | RefSeq_532_3763 | SLC17A2 | 0.963734 |
| ENST00000545531 | DBH | 0.953328596 | RefSeq_1589_2127 | SLC17A2 | 0.95142 |
| ENCODE_981_731 | DBH | 0.95078866 | RefSeq_1769_1968 | SLC17A2 | 0.97056 |
| RefSeq_2351_1430 | DBH | 0.969113116 | UCSC_3328_2475 | SLC17A2 | 0.969107 |
| XLOC_002019 | DCXR | 0.958152121 | UCSC_3328_2475 | SLC17A2 | 0.957746 |
| UCSC_6534_1640 | DCXR | 0.954964686 | UCSC_4189_2198 | SLC17A2 | 0.957918 |
| UCSC_5210_1945 | DEPDC7 | 0.95039482 | UCSC_5722_1825 | SLC17A2 | 0.959553 |
| XLOC_002845 | DHTKD1 | 0.955685966 | UCSC_5722_1825 | SLC17A2 | 0.971108 |
| ENST00000538369 | DHTKD1 | 0.95125203 | UCSC_6534_1640 | SLC17A2 | 0.961516 |
| ENCODE_2110_447 | DHTKD1 | 0.957122773 | UCSC_11594_306 | SLC17A2 | 0.972082 |
| UCSC_5722_1825 | DHTKD1 | 0.962130369 | ENST00000417851 | SLC17A3 | 0.952614 |
| XLOC_012693 | DIO1 | 0.978655437 | NRED_1255_416 | SLC17A3 | 0.966485 |
| XLOC_001387 | DIO1 | 0.954652836 | ENCODE_2110_447 | SLC17A3 | 0.968427 |
| XLOC_002845 | DIO1 | 0.961669929 | RefSeq_1589_2127 | SLC17A3 | 0.972376 |
| XLOC_003572 | DIO1 | 0.958100289 | RefSeq_2351_1430 | SLC17A3 | 0.951256 |
| ENST00000507582 | DIO1 | 0.973444795 | UCSC_5722_1825 | SLC17A3 | 0.96993 |
| ENST00000544018 | DIO1 | 0.961932274 | UCSC_11594_306 | SLC17A3 | 0.950323 |
| ENST00000417121 | DIO1 | 0.975731336 | XLOC_001387 | SLC1A2 | 0.955316 |
| ENST00000417851 | DIO1 | 0.966756594 | ENST00000507582 | SLC1A2 | 0.964394 |
| ENST00000439524 | DIO1 | 0.9877345 | ENST00000544018 | SLC1A2 | 0.960236 |
| ENST00000414604 | DIO1 | 0.986526943 | ENST00000376608 | SLC1A2 | 0.959537 |
| ENST00000445096 | DIO1 | 0.977737912 | UCSC_5722_1825 | SLC1A2 | 0.958396 |
| ENST00000545531 | DIO1 | 0.973430597 | XLOC_012693 | SLC22A1 | 0.95405 |
| ENCODE_2110_447 | DIO1 | 0.953486256 | XLOC_003572 | SLC22A1 | 0.95431 |
| RefSeq_532_3763 | DIO1 | 0.973092513 | ENST00000544018 | SLC22A1 | 0.951168 |
| RefSeq_1589_2127 | DIO1 | 0.954413897 | ENST00000417121 | SLC22A1 | 0.953881 |
| RefSeq_2351_1430 | DIO1 | 0.966869059 | ENST00000417851 | SLC22A1 | 0.952497 |
| RefSeq_2866_939 | DIO1 | 0.951201276 | ENST00000445096 | SLC22A1 | 0.950576 |
| UCSC_5722_1825 | DIO1 | 0.981203418 | ENCODE_1597_545 | SLC22A1 | 0.958679 |
| UCSC_6534_1640 | DIO1 | 0.958364876 | ENCODE_2635_348 | SLC22A1 | 0.96493 |
| UCSC_11594_306 | DIO1 | 0.965567204 | RefSeq_532_3763 | SLC22A1 | 0.95344 |
| CombinedLit_370_438 | DIO1 | 0.982740975 | UCSC_6534_1640 | SLC22A1 | 0.967123 |
| XLOC_012693 | DMGDH | 0.973051085 | XLOC_012693 | SLC22A10 | 0.956549 |
| XLOC_001387 | DMGDH | 0.95371384 | XLOC_012693 | SLC22A3 | 0.978544 |
| XLOC_002845 | DMGDH | 0.954573952 | ENST00000417121 | SLC22A3 | 0.963644 |
| XLOC_003572 | DMGDH | 0.967228654 | ENST00000439524 | SLC22A3 | 0.977021 |
| XLOC_004515 | DMGDH | 0.950610821 | ENST00000414604 | SLC22A3 | 0.979272 |
| ENST00000507582 | DMGDH | 0.967248619 | ENST00000445096 | SLC22A3 | 0.973452 |
| ENST00000544018 | DMGDH | 0.975029717 | ENST00000545531 | SLC22A3 | 0.97067 |
| ENST00000417121 | DMGDH | 0.958204302 | RefSeq_532_3763 | SLC22A3 | 0.957362 |
| ENST00000417851 | DMGDH | 0.955829954 | RefSeq_2866_939 | SLC22A3 | 0.961549 |
| ENST00000439524 | DMGDH | 0.96749594 | RefSeq_3051_754 | SLC22A3 | 0.963811 |
| ENST00000414604 | DMGDH | 0.964230096 | UCSC_3328_2475 | SLC22A3 | 0.951275 |
| ENST00000445096 | DMGDH | 0.95908639 | CombinedLit_370_438 | SLC22A3 | 0.978739 |
| ENST00000545531 | DMGDH | 0.957365537 | XLOC_012693 | SLC22A7 | 0.972614 |
| NRED_1255_416 | DMGDH | 0.955118576 | XLOC_003572 | SLC22A7 | 0.975949 |
| ENCODE_1597_545 | DMGDH | 0.959443433 | ENST00000417121 | SLC22A7 | 0.964763 |
| ENCODE_2110_447 | DMGDH | 0.96506559 | ENST00000417851 | SLC22A7 | 0.957905 |
| RefSeq_532_3763 | DMGDH | 0.978947245 | ENST00000439524 | SLC22A7 | 0.965995 |
| RefSeq_1589_2127 | DMGDH | 0.952785982 | ENST00000414604 | SLC22A7 | 0.964079 |
| RefSeq_2351_1430 | DMGDH | 0.952119291 | ENST00000445096 | SLC22A7 | 0.976638 |
| UCSC_5722_1825 | DMGDH | 0.965716056 | ENST00000545531 | SLC22A7 | 0.961168 |
| UCSC_6534_1640 | DMGDH | 0.958990404 | RefSeq_532_3763 | SLC22A7 | 0.953308 |
| UCSC_10583_636 | DMGDH | 0.952995256 | RefSeq_2866_939 | SLC22A7 | 0.952709 |
| UCSC_11594_306 | DMGDH | 0.964247778 | UCSC_3328_2475 | SLC22A7 | 0.968491 |
| CombinedLit_370_438 | DMGDH | 0.966019009 | UCSC_11594_306 | SLC22A7 | 0.951075 |
| RefSeq_2991_815 | DNAJB3 | 0.954706574 | CombinedLit_370_438 | SLC22A7 | 0.964323 |
| RefSeq_3121_678 | DNAJB3 | 0.956301974 | XLOC_002019 | SLC23A1 | 0.956657 |
| XLOC_012693 | DPYS | 0.9833413 | UCSC_6534_1640 | SLC23A1 | 0.9686 |
| XLOC_003572 | DPYS | 0.961345901 | ENST00000417121 | SLC25A18 | 0.956686 |
| XLOC_004515 | DPYS | 0.961684085 | ENST00000417851 | SLC25A18 | 0.952179 |
| ENST00000507582 | DPYS | 0.956834869 | ENST00000439524 | SLC25A18 | 0.95881 |
| ENST00000544018 | DPYS | 0.972955115 | ENCODE_2110_447 | SLC25A18 | 0.962966 |
| ENST00000417121 | DPYS | 0.970891302 | RefSeq_2351_1430 | SLC25A18 | 0.957591 |
| ENST00000417851 | DPYS | 0.964969665 | XLOC_012693 | SLC25A47 | 0.981966 |
| ENST00000439524 | DPYS | 0.979930139 | ENST00000544018 | SLC25A47 | 0.960539 |
| ENST00000414604 | DPYS | 0.974158533 | ENST00000417121 | SLC25A47 | 0.973804 |
| ENST00000445096 | DPYS | 0.969333923 | ENST00000417851 | SLC25A47 | 0.964439 |
| ENST00000545531 | DPYS | 0.965961268 | ENST00000439524 | SLC25A47 | 0.981008 |
| NRED_1255_416 | DPYS | 0.95471348 | ENST00000414604 | SLC25A47 | 0.977357 |
| ENCODE_1597_545 | DPYS | 0.965401982 | ENST00000445096 | SLC25A47 | 0.971155 |
| ENCODE_2110_447 | DPYS | 0.964780792 | ENST00000545531 | SLC25A47 | 0.969806 |
| RefSeq_532_3763 | DPYS | 0.98597628 | lncRNAdb_60_500 | SLC25A47 | 0.956162 |
| RefSeq_1589_2127 | DPYS | 0.951897577 | NRED_1255_416 | SLC25A47 | 0.969763 |
| RefSeq_1769_1968 | DPYS | 0.950046272 | ENCODE_2110_447 | SLC25A47 | 0.950059 |
| RefSeq_2351_1430 | DPYS | 0.964485834 | RefSeq_532_3763 | SLC25A47 | 0.982774 |
| UCSC_5722_1825 | DPYS | 0.955568881 | RefSeq_1589_2127 | SLC25A47 | 0.961274 |
| UCSC_6534_1640 | DPYS | 0.966571498 | RefSeq_2351_1430 | SLC25A47 | 0.98322 |
| UCSC_8524_1151 | DPYS | 0.958167928 | UCSC_5722_1825 | SLC25A47 | 0.950226 |
| UCSC_11594_306 | DPYS | 0.969684528 | UCSC_6534_1640 | SLC25A47 | 0.965588 |
| CombinedLit_370_438 | DPYS | 0.975015178 | UCSC_8524_1151 | SLC25A47 | 0.972661 |
| ENST00000417851 | ECHDC2 | 0.959637498 | UCSC_11594_306 | SLC25A47 | 0.959656 |
| UCSC_6534_1640 | ECHDC2 | 0.970373095 | CombinedLit_370_438 | SLC25A47 | 0.984595 |
| XLOC_002845 | ETNK2 | 0.959668924 | XLOC_012693 | SLC27A5 | 0.964787 |
| ENST00000544018 | ETNK2 | 0.976273151 | ENST00000544018 | SLC27A5 | 0.970431 |
| ENCODE_456_1286 | ETNK2 | 0.961530635 | ENST00000417121 | SLC27A5 | 0.966447 |
| ENCODE_1931_477 | ETNK2 | 0.953131418 | ENST00000417851 | SLC27A5 | 0.966595 |
| UCSC_5722_1825 | ETNK2 | 0.963417281 | ENST00000439524 | SLC27A5 | 0.96831 |
| UCSC_6534_1640 | ETNK2 | 0.95368933 | ENST00000414604 | SLC27A5 | 0.962993 |
| XLOC_012693 | F11 | 0.970083208 | ENST00000445096 | SLC27A5 | 0.963805 |
| XLOC_012693 | F11 | 0.977262321 | ENST00000545531 | SLC27A5 | 0.966512 |
| XLOC_003572 | F11 | 0.954269768 | RefSeq_532_3763 | SLC27A5 | 0.974199 |
| XLOC_004515 | F11 | 0.958080493 | RefSeq_1589_2127 | SLC27A5 | 0.956398 |
| ENST00000417121 | F11 | 0.958781918 | RefSeq_2351_1430 | SLC27A5 | 0.964629 |
| ENST00000417121 | F11 | 0.966128856 | UCSC_6534_1640 | SLC27A5 | 0.980175 |
| ENST00000417851 | F11 | 0.953994525 | CombinedLit_370_438 | SLC27A5 | 0.961348 |
| ENST00000417851 | F11 | 0.965391088 | XLOC_002013 | SLC28A1 | 0.952822 |
| ENST00000439524 | F11 | 0.964204201 | ENCODE_8_5492 | SLC28A1 | 0.951756 |
| ENST00000439524 | F11 | 0.968810077 | UCSC_11415_368 | SLC28A1 | 0.957297 |
| ENST00000414604 | F11 | 0.960159546 | ENST00000505626 | SLC2A2 | 0.95785 |
| ENST00000414604 | F11 | 0.961155672 | RefSeq_843_3032 | SLC2A2 | 0.9681 |
| ENST00000445096 | F11 | 0.96733701 | RefSeq_869_3001 | SLC2A2 | 0.963858 |
| ENST00000445096 | F11 | 0.969545111 | asoverlaps_682_1255 | SLC2A2 | 0.966356 |
| ENST00000545531 | F11 | 0.959357387 | RNAz_5553_200 | SLC2A2 | 0.951245 |
| ENST00000545531 | F11 | 0.961580209 | UCSC_5722_1825 | SLC2A9 | 0.966384 |
| ENCODE_1597_545 | F11 | 0.951986073 | ENST00000545531 | SLC37A4 | 0.954909 |
| ENCODE_1597_545 | F11 | 0.965622169 | UCSC_6534_1640 | SLC37A4 | 0.962375 |
| ENCODE_2110_447 | F11 | 0.954750298 | XLOC_012693 | SLC38A3 | 0.984502 |
| RefSeq_532_3763 | F11 | 0.959572535 | XLOC_003572 | SLC38A3 | 0.969607 |
| RefSeq_532_3763 | F11 | 0.967694129 | ENST00000544018 | SLC38A3 | 0.950446 |
| UCSC_6534_1640 | F11 | 0.967579626 | ENST00000417121 | SLC38A3 | 0.984883 |
| UCSC_6534_1640 | F11 | 0.973341769 | ENST00000417851 | SLC38A3 | 0.980359 |
| UCSC_11594_306 | F11 | 0.950599637 | ENST00000439524 | SLC38A3 | 0.983493 |
| CombinedLit_370_438 | F11 | 0.959456519 | ENST00000414604 | SLC38A3 | 0.980416 |
| CombinedLit_370_438 | F11 | 0.957696619 | ENST00000445096 | SLC38A3 | 0.991889 |
| XLOC_012693 | F13B | 0.977971952 | ENST00000545531 | SLC38A3 | 0.987522 |
| XLOC_003572 | F13B | 0.952931766 | RefSeq_532_3763 | SLC38A3 | 0.973354 |
| XLOC_004515 | F13B | 0.960429956 | RefSeq_2351_1430 | SLC38A3 | 0.971762 |
| ENST00000507582 | F13B | 0.96418852 | RefSeq_3051_754 | SLC38A3 | 0.952636 |
| ENST00000376608 | F13B | 0.954584309 | UCSC_6534_1640 | SLC38A3 | 0.962613 |
| ENST00000417121 | F13B | 0.958341501 | CombinedLit_370_438 | SLC38A3 | 0.986341 |
| ENST00000439524 | F13B | 0.97495478 | XLOC_012693 | SLC38A4 | 0.984042 |
| ENST00000414604 | F13B | 0.965038742 | XLOC_012693 | SLC38A4 | 0.982405 |
| ENST00000445096 | F13B | 0.962419132 | XLOC_004515 | SLC38A4 | 0.958891 |
| ENST00000545531 | F13B | 0.953113999 | XLOC_004515 | SLC38A4 | 0.963242 |
| lncRNAdb_60_500 | F13B | 0.967306388 | ENST00000417121 | SLC38A4 | 0.97866 |
| NRED_1255_416 | F13B | 0.958707657 | ENST00000417121 | SLC38A4 | 0.976018 |
| ENCODE_795_804 | F13B | 0.950966469 | ENST00000417851 | SLC38A4 | 0.969722 |
| ENCODE_1597_545 | F13B | 0.955584998 | ENST00000417851 | SLC38A4 | 0.965164 |
| ENCODE_2110_447 | F13B | 0.969591673 | ENST00000439524 | SLC38A4 | 0.976635 |
| RefSeq_532_3763 | F13B | 0.973795928 | ENST00000439524 | SLC38A4 | 0.983233 |
| RefSeq_1589_2127 | F13B | 0.964014088 | ENST00000414604 | SLC38A4 | 0.966268 |
| RefSeq_1769_1968 | F13B | 0.9744151 | ENST00000414604 | SLC38A4 | 0.974053 |
| UCSC_5722_1825 | F13B | 0.955299318 | ENST00000445096 | SLC38A4 | 0.973484 |
| UCSC_8524_1151 | F13B | 0.971179215 | ENST00000445096 | SLC38A4 | 0.971523 |
| UCSC_11594_306 | F13B | 0.980392798 | ENST00000545531 | SLC38A4 | 0.972485 |
| CombinedLit_370_438 | F13B | 0.969174351 | ENST00000545531 | SLC38A4 | 0.969586 |
| XLOC_012693 | F2 | 0.977905554 | lncRNAdb_60_500 | SLC38A4 | 0.953323 |
| ENST00000417121 | F2 | 0.963364885 | NRED_1255_416 | SLC38A4 | 0.950759 |
| ENST00000439524 | F2 | 0.962200516 | ENCODE_1597_545 | SLC38A4 | 0.950509 |
| ENST00000414604 | F2 | 0.951894476 | RefSeq_532_3763 | SLC38A4 | 0.985593 |
| ENST00000445096 | F2 | 0.962411488 | RefSeq_532_3763 | SLC38A4 | 0.98481 |
| ENST00000545531 | F2 | 0.951488005 | RefSeq_1589_2127 | SLC38A4 | 0.954596 |
| lncRNAdb_60_500 | F2 | 0.971479613 | RefSeq_1589_2127 | SLC38A4 | 0.966007 |
| RefSeq_532_3763 | F2 | 0.977249563 | RefSeq_2351_1430 | SLC38A4 | 0.969697 |
| RefSeq_2351_1430 | F2 | 0.952168095 | RefSeq_2351_1430 | SLC38A4 | 0.968246 |
| UCSC_8524_1151 | F2 | 0.979705587 | UCSC_5722_1825 | SLC38A4 | 0.955601 |
| UCSC_11594_306 | F2 | 0.976168025 | UCSC_6534_1640 | SLC38A4 | 0.963112 |
| CombinedLit_370_438 | F2 | 0.978709535 | UCSC_6534_1640 | SLC38A4 | 0.968784 |
| ENCODE_2110_447 | F7 | 0.953718426 | UCSC_8524_1151 | SLC38A4 | 0.969097 |
| H-InvDB_178_818 | F7 | 0.952610648 | UCSC_8524_1151 | SLC38A4 | 0.969255 |
| XLOC_012693 | F9 | 0.985862661 | UCSC_11594_306 | SLC38A4 | 0.957655 |
| XLOC_003572 | F9 | 0.954422813 | UCSC_11594_306 | SLC38A4 | 0.969904 |
| XLOC_004515 | F9 | 0.955876602 | CombinedLit_370_438 | SLC38A4 | 0.978947 |
| ENST00000507582 | F9 | 0.954537196 | CombinedLit_370_438 | SLC38A4 | 0.977254 |
| ENST00000417121 | F9 | 0.967838866 | ENCODE_1931_477 | SLC43A1 | 0.950596 |
| ENST00000417851 | F9 | 0.958316458 | RefSeq_843_3032 | SLC43A1 | 0.956644 |
| ENST00000439524 | F9 | 0.976270038 | XLOC_012693 | SLC47A1 | 0.970363 |
| ENST00000414604 | F9 | 0.969430258 | XLOC_002845 | SLC47A1 | 0.963255 |
| ENST00000445096 | F9 | 0.969858073 | XLOC_003572 | SLC47A1 | 0.951098 |
| ENST00000545531 | F9 | 0.957700953 | XLOC_003572 | SLC47A1 | 0.966512 |
| lncRNAdb_60_500 | F9 | 0.970705239 | XLOC_004515 | SLC47A1 | 0.9537 |
| ENCODE_1597_545 | F9 | 0.952002783 | ENST00000507582 | SLC47A1 | 0.966015 |
| ENCODE_2110_447 | F9 | 0.968355236 | ENST00000544018 | SLC47A1 | 0.979293 |
| RefSeq_532_3763 | F9 | 0.981049127 | ENST00000544018 | SLC47A1 | 0.951977 |
| RefSeq_1769_1968 | F9 | 0.962470915 | ENST00000417121 | SLC47A1 | 0.95615 |
| RefSeq_2351_1430 | F9 | 0.952292563 | ENST00000439524 | SLC47A1 | 0.963111 |
| UCSC_3328_2475 | F9 | 0.951525556 | ENST00000414604 | SLC47A1 | 0.955722 |
| UCSC_5722_1825 | F9 | 0.950820558 | ENST00000445096 | SLC47A1 | 0.961548 |
| UCSC_8524_1151 | F9 | 0.967206473 | ENST00000545531 | SLC47A1 | 0.953877 |
| UCSC_11594_306 | F9 | 0.987173347 | ENCODE_456_1286 | SLC47A1 | 0.951918 |
| CombinedLit_370_438 | F9 | 0.979997282 | RefSeq_532_3763 | SLC47A1 | 0.952189 |
| UCSC_8408_1181 | FAAH | 0.965436578 | RefSeq_532_3763 | SLC47A1 | 0.962791 |
| ENST00000417121 | FAH | 0.950095814 | UCSC_3328_2475 | SLC47A1 | 0.958464 |
| ENST00000417851 | FAH | 0.952340282 | UCSC_5722_1825 | SLC47A1 | 0.971528 |
| RefSeq_532_3763 | FAH | 0.950775625 | UCSC_11594_306 | SLC47A1 | 0.966263 |
| UCSC_6534_1640 | FAH | 0.95641393 | CombinedLit_370_438 | SLC47A1 | 0.965662 |
| XLOC_002845 | FAHD2A | 0.950984639 | XLOC_001387 | SLC6A12 | 0.956007 |
| XLOC_012693 | FAM176A | 0.954635486 | ENST00000507582 | SLC6A12 | 0.960742 |
| XLOC_003572 | FAM176A | 0.953586092 | ENST00000538369 | SLC6A12 | 0.960915 |
| ENST00000417851 | FAM176A | 0.9516837 | ENST00000376608 | SLC6A12 | 0.965421 |
| RefSeq_532_3763 | FAM176A | 0.963965273 | ENST00000507582 | SLCO1B1 | 0.953983 |
| RefSeq_532_3763 | FAM176A | 0.955247161 | ENST00000538369 | SLCO1B1 | 0.950041 |
| RefSeq_1589_2127 | FAM176A | 0.953671414 | ENST00000376608 | SLCO1B1 | 0.951727 |
| UCSC_8524_1151 | FAM176A | 0.9618287 | ENCODE_2110_447 | SLCO1B1 | 0.951352 |
| UCSC_11594_306 | FAM176A | 0.954680127 | ENCODE_2461_388 | SLCO1B1 | 0.951835 |
| XLOC_012693 | FAM99A | 0.973886055 | RefSeq_843_3032 | SLCO1B1 | 0.967529 |
| ENST00000417121 | FAM99A | 0.978311627 | RefSeq_869_3001 | SLCO1B1 | 0.959287 |
| ENST00000417851 | FAM99A | 0.97011741 | RefSeq_1769_1968 | SLCO1B1 | 0.956912 |
| ENST00000439524 | FAM99A | 0.970057799 | UCSC_4189_2198 | SLCO1B1 | 0.952573 |
| ENST00000414604 | FAM99A | 0.961448369 | UCSC_5210_1945 | SLCO1B1 | 0.957787 |
| ENST00000445096 | FAM99A | 0.973814262 | UCSC_11594_306 | SLCO1B1 | 0.958855 |
| ENST00000545531 | FAM99A | 0.979594091 | asoverlaps_682_1255 | SLCO1B1 | 0.95557 |
| lncRNAdb_60_500 | FAM99A | 0.950515455 | NRED_1255_416 | SLCO1B3 | 0.955209 |
| RefSeq_532_3763 | FAM99A | 0.968286354 | XLOC_012693 | SMOC1 | 0.959387 |
| RefSeq_2351_1430 | FAM99A | 0.997935165 | ENST00000417121 | SMOC1 | 0.950902 |
| UCSC_6534_1640 | FAM99A | 0.9678272 | ENST00000439524 | SMOC1 | 0.956363 |
| UCSC_8524_1151 | FAM99A | 0.959598046 | ENST00000414604 | SMOC1 | 0.950002 |
| CombinedLit_370_438 | FAM99A | 0.97807852 | ENST00000445096 | SMOC1 | 0.957712 |
| XLOC_012693 | FAM99B | 0.968833259 | CombinedLit_370_438 | SMOC1 | 0.951552 |
| ENST00000544018 | FAM99B | 0.964735809 | XLOC_012693 | SPP2 | 0.976507 |
| ENST00000417121 | FAM99B | 0.97323845 | ENST00000507582 | SPP2 | 0.965817 |
| ENST00000417851 | FAM99B | 0.968556779 | ENST00000417121 | SPP2 | 0.967372 |
| ENST00000439524 | FAM99B | 0.980648359 | ENST00000417851 | SPP2 | 0.954044 |
| ENST00000414604 | FAM99B | 0.975246993 | ENST00000439524 | SPP2 | 0.976032 |
| ENST00000445096 | FAM99B | 0.967540298 | ENST00000414604 | SPP2 | 0.966652 |
| ENST00000545531 | FAM99B | 0.97022427 | ENST00000445096 | SPP2 | 0.971695 |
| RefSeq_532_3763 | FAM99B | 0.967639834 | ENST00000545531 | SPP2 | 0.961997 |
| RefSeq_2351_1430 | FAM99B | 0.981573268 | lncRNAdb_60_500 | SPP2 | 0.964733 |
| UCSC_5722_1825 | FAM99B | 0.970162139 | NRED_1255_416 | SPP2 | 0.950805 |
| UCSC_6534_1640 | FAM99B | 0.964569503 | ENCODE_795_804 | SPP2 | 0.951809 |
| CombinedLit_370_438 | FAM99B | 0.966452367 | ENCODE_2110_447 | SPP2 | 0.964027 |
| XLOC_012693 | FCN2 | 0.968691294 | RefSeq_532_3763 | SPP2 | 0.966324 |
| XLOC_004515 | FCN2 | 0.964214283 | RefSeq_843_3032 | SPP2 | 0.954016 |
| ENST00000544018 | FCN2 | 0.953050819 | RefSeq_1589_2127 | SPP2 | 0.958163 |
| ENST00000417121 | FCN2 | 0.965013995 | RefSeq_1769_1968 | SPP2 | 0.962935 |
| ENST00000417851 | FCN2 | 0.963628329 | UCSC_5722_1825 | SPP2 | 0.957202 |
| ENST00000439524 | FCN2 | 0.96454386 | UCSC_5979_1768 | SPP2 | 0.954938 |
| ENST00000414604 | FCN2 | 0.950952326 | UCSC_8524_1151 | SPP2 | 0.956465 |
| ENST00000445096 | FCN2 | 0.952755732 | UCSC_11594_306 | SPP2 | 0.978194 |
| ENST00000545531 | FCN2 | 0.95469112 | CombinedLit_370_438 | SPP2 | 0.974203 |
| NRED_1255_416 | FCN2 | 0.966863715 | ENST00000417851 | STEAP3 | 0.953256 |
| ENCODE_981_731 | FCN2 | 0.959495434 | UCSC_3488_2413 | STEAP3 | 0.951453 |
| ENCODE_1597_545 | FCN2 | 0.951783989 | UCSC_6534_1640 | STEAP3 | 0.958314 |
| RefSeq_532_3763 | FCN2 | 0.977248197 | XLOC_012693 | SULT2A1 | 0.984877 |
| RefSeq_1589_2127 | FCN2 | 0.96441326 | ENST00000507582 | SULT2A1 | 0.96337 |
| RefSeq_2351_1430 | FCN2 | 0.970740889 | ENST00000417121 | SULT2A1 | 0.964798 |
| UCSC_6534_1640 | FCN2 | 0.974228934 | ENST00000417851 | SULT2A1 | 0.950166 |
| UCSC_8524_1151 | FCN2 | 0.962003055 | ENST00000439524 | SULT2A1 | 0.984271 |
| CombinedLit_370_438 | FCN2 | 0.954869726 | ENST00000414604 | SULT2A1 | 0.985498 |
| XLOC_012693 | FCN3 | 0.954984161 | ENST00000445096 | SULT2A1 | 0.972445 |
| ENST00000417121 | FCN3 | 0.957678298 | ENST00000545531 | SULT2A1 | 0.960813 |
| ENST00000545531 | FCN3 | 0.95196098 | ENCODE_1597_545 | SULT2A1 | 0.951925 |
| lncRNAdb_60_500 | FCN3 | 0.956505183 | RefSeq_532_3763 | SULT2A1 | 0.973994 |
| RefSeq_532_3763 | FCN3 | 0.958803773 | RefSeq_1769_1968 | SULT2A1 | 0.964719 |
| RefSeq_2351_1430 | FCN3 | 0.966443159 | RefSeq_2866_939 | SULT2A1 | 0.953405 |
| UCSC_8524_1151 | FCN3 | 0.963751744 | RefSeq_3175_631 | SULT2A1 | 0.950976 |
| UCSC_11594_306 | FCN3 | 0.95166878 | UCSC_3328_2475 | SULT2A1 | 0.956071 |
| RefSeq_107_6579 | FER1L4 | 0.963344376 | UCSC_5722_1825 | SULT2A1 | 0.95416 |
| XLOC_012693 | FETUB | 0.953246396 | UCSC_11594_306 | SULT2A1 | 0.970126 |
| ENST00000417121 | FETUB | 0.958122696 | CombinedLit_370_438 | SULT2A1 | 0.984266 |
| ENST00000417851 | FETUB | 0.959650198 | XLOC_012693 | TAT | 0.960035 |
| ENST00000439524 | FETUB | 0.954535763 | XLOC_002845 | TAT | 0.950083 |
| ENST00000445096 | FETUB | 0.958225848 | XLOC_003572 | TAT | 0.950054 |
| ENST00000545531 | FETUB | 0.950221458 | ENST00000507582 | TAT | 0.966024 |
| lncRNAdb_60_500 | FETUB | 0.953842374 | ENST00000538369 | TAT | 0.955598 |
| NRED_1255_416 | FETUB | 0.959440116 | ENST00000417121 | TAT | 0.953623 |
| ENCODE_2635_348 | FETUB | 0.954347542 | ENST00000439524 | TAT | 0.976455 |
| XLOC_012693 | FGA | 0.957707453 | ENST00000414604 | TAT | 0.971407 |
| lncRNAdb_60_500 | FGA | 0.961437511 | ENST00000445096 | TAT | 0.957339 |
| RefSeq_532_3763 | FGA | 0.960029015 | ENCODE_2110_447 | TAT | 0.961967 |
| RefSeq_532_3763 | FGA | 0.953348693 | RefSeq_532_3763 | TAT | 0.959493 |
| RefSeq_532_3763 | FGA | 0.956106324 | RefSeq_1769_1968 | TAT | 0.965881 |
| RefSeq_1769_1968 | FGA | 0.967528188 | UCSC_3328_2475 | TAT | 0.957159 |
| UCSC_8524_1151 | FGA | 0.963644812 | UCSC_4189_2198 | TAT | 0.953259 |
| UCSC_8524_1151 | FGA | 0.953671746 | UCSC_5722_1825 | TAT | 0.970553 |
| UCSC_11594_306 | FGA | 0.982879785 | UCSC_11594_306 | TAT | 0.981605 |
| UCSC_11594_306 | FGA | 0.95606955 | CombinedLit_370_438 | TAT | 0.956291 |
| CombinedLit_370_438 | FGA | 0.952563517 | ENST00000447936 | TCP10L | 0.952259 |
| lncRNAdb_60_500 | FGB | 0.959187018 | ENCODE_1035_709 | TCP10L | 0.959631 |
| RefSeq_532_3763 | FGB | 0.958394318 | ENCODE_2110_447 | TCP10L | 0.951885 |
| UCSC_8524_1151 | FGB | 0.973688446 | ENCODE_795_804 | TF | 0.961143 |
| UCSC_11594_306 | FGB | 0.97299592 | XLOC_012693 | TFR2 | 0.974716 |
| CombinedLit_370_438 | FGB | 0.953082729 | XLOC_004515 | TFR2 | 0.950933 |
| XLOC_012693 | FGL1 | 0.96920012 | ENST00000417121 | TFR2 | 0.968694 |
| XLOC_004515 | FGL1 | 0.953090861 | ENST00000417851 | TFR2 | 0.953928 |
| ENST00000439524 | FGL1 | 0.95930015 | ENST00000439524 | TFR2 | 0.987063 |
| lncRNAdb_60_500 | FGL1 | 0.961934777 | ENST00000414604 | TFR2 | 0.984272 |
| NRED_1255_416 | FGL1 | 0.956822852 | ENST00000445096 | TFR2 | 0.972544 |
| ENCODE_981_731 | FGL1 | 0.951629856 | ENST00000545531 | TFR2 | 0.969197 |
| RefSeq_532_3763 | FGL1 | 0.979219653 | RefSeq_532_3763 | TFR2 | 0.964516 |
| RefSeq_1589_2127 | FGL1 | 0.958636515 | RefSeq_2351_1430 | TFR2 | 0.959309 |
| RefSeq_2351_1430 | FGL1 | 0.961385608 | RefSeq_3051_754 | TFR2 | 0.951674 |
| UCSC_8524_1151 | FGL1 | 0.986973894 | UCSC_6534_1640 | TFR2 | 0.95648 |
| UCSC_11594_306 | FGL1 | 0.964768872 | CombinedLit_370_438 | TFR2 | 0.976206 |
| CombinedLit_370_438 | FGL1 | 0.96166527 | XLOC_004398 | THPO | 0.954841 |
| RefSeq_1361_2328 | FLJ41200 | 0.957516236 | ENCODE_2018_465 | THPO | 0.967207 |
| XLOC_012693 | FMO3 | 0.950792302 | RefSeq_2351_1430 | THPO | 0.960781 |
| XLOC_002845 | FMO3 | 0.951554618 | UCSC_6534_1640 | THPO | 0.950526 |
| ENST00000507582 | FMO3 | 0.964429755 | ENST00000544018 | THRSP | 0.95379 |
| ENST00000376608 | FMO3 | 0.957888047 | XLOC_012693 | TMEM195 | 0.959073 |
| ENST00000417121 | FMO3 | 0.955569968 | ENST00000439524 | TMEM195 | 0.958659 |
| ENST00000439524 | FMO3 | 0.956322713 | lncRNAdb_60_500 | TMEM195 | 0.950782 |
| RefSeq_532_3763 | FMO3 | 0.950695525 | NRED_1255_416 | TMEM195 | 0.962709 |
| RefSeq_1589_2127 | FMO3 | 0.951477576 | ENCODE_2110_447 | TMEM195 | 0.963299 |
| RefSeq_2351_1430 | FMO3 | 0.950311125 | RefSeq_1589_2127 | TMEM195 | 0.951524 |
| UCSC_5722_1825 | FMO3 | 0.974852359 | RefSeq_1589_2127 | TMEM27 | 0.950718 |
| UCSC_11594_306 | FMO3 | 0.957376107 | XLOC_012693 | TMPRSS6 | 0.96396 |
| CombinedLit_370_438 | FMO3 | 0.954050824 | ENST00000417121 | TMPRSS6 | 0.973226 |
| XLOC_012693 | FTCD | 0.984339605 | ENST00000417851 | TMPRSS6 | 0.972487 |
| XLOC_012693 | FTCD | 0.986442375 | ENST00000445096 | TMPRSS6 | 0.974321 |
| XLOC_003572 | FTCD | 0.951438605 | ENST00000545531 | TMPRSS6 | 0.973228 |
| XLOC_003572 | FTCD | 0.955037415 | RefSeq_2351_1430 | TMPRSS6 | 0.951407 |
| ENST00000544018 | FTCD | 0.950036261 | UCSC_6534_1640 | TMPRSS6 | 0.956271 |
| ENST00000417121 | FTCD | 0.980560919 | CombinedLit_370_438 | TMPRSS6 | 0.959083 |
| ENST00000417121 | FTCD | 0.981998787 | XLOC_002013 | TPPP2 | 0.955483 |
| ENST00000417851 | FTCD | 0.975884352 | RefSeq_843_3032 | TRPM8 | 0.953876 |
| ENST00000417851 | FTCD | 0.975827228 | RefSeq_869_3001 | TRPM8 | 0.951601 |
| ENST00000439524 | FTCD | 0.983256581 | XLOC_004515 | TTPA | 0.955353 |
| ENST00000439524 | FTCD | 0.98270511 | ENST00000505626 | TTPA | 0.958756 |
| ENST00000414604 | FTCD | 0.980339096 | ENST00000507582 | TTPA | 0.95376 |
| ENST00000414604 | FTCD | 0.979192051 | RefSeq_843_3032 | TTPA | 0.971998 |
| ENST00000445096 | FTCD | 0.982952427 | RefSeq_869_3001 | TTPA | 0.961084 |
| ENST00000445096 | FTCD | 0.985465117 | RefSeq_1769_1968 | TTPA | 0.965788 |
| ENST00000545531 | FTCD | 0.973888917 | UCSC_3328_2475 | TTPA | 0.958075 |
| ENST00000545531 | FTCD | 0.980563375 | UCSC_4189_2198 | TTPA | 0.951504 |
| ENCODE_1597_545 | FTCD | 0.952049239 | UCSC_11594_306 | TTPA | 0.9564 |
| RefSeq_532_3763 | FTCD | 0.982755317 | asoverlaps_682_1255 | TTPA | 0.963174 |
| RefSeq_532_3763 | FTCD | 0.980443714 | XLOC_012693 | TTR | 0.969675 |
| RefSeq_2351_1430 | FTCD | 0.961199918 | ENST00000417121 | TTR | 0.962113 |
| RefSeq_2351_1430 | FTCD | 0.966660332 | ENST00000417851 | TTR | 0.964119 |
| UCSC_6534_1640 | FTCD | 0.96721039 | ENST00000439524 | TTR | 0.965509 |
| UCSC_6534_1640 | FTCD | 0.971116458 | ENST00000414604 | TTR | 0.961992 |
| UCSC_8524_1151 | FTCD | 0.950908648 | ENST00000445096 | TTR | 0.965596 |
| UCSC_11594_306 | FTCD | 0.956153964 | ENST00000545531 | TTR | 0.956092 |
| CombinedLit_370_438 | FTCD | 0.985359006 | NRED_1255_416 | TTR | 0.960347 |
| CombinedLit_370_438 | FTCD | 0.985345865 | RefSeq_532_3763 | TTR | 0.967883 |
| RefSeq_843_3032 | G6PC | 0.950729714 | RefSeq_2351_1430 | TTR | 0.954411 |
| UCSC_3328_2475 | G6PC | 0.965007657 | UCSC_6534_1640 | TTR | 0.970347 |
| XLOC_012693 | GAMT | 0.965666086 | CombinedLit_370_438 | TTR | 0.964903 |
| XLOC_003572 | GAMT | 0.950271058 | UCSC_6631_1619 | UBXN8 | 0.961822 |
| ENST00000544018 | GAMT | 0.955510187 | UCSC_8286_1209 | UBXN8 | 0.983638 |
| ENST00000417121 | GAMT | 0.965542672 | XLOC_012693 | UGT2B10 | 0.963297 |
| ENST00000417851 | GAMT | 0.9638268 | XLOC_003572 | UGT2B10 | 0.950471 |
| ENST00000439524 | GAMT | 0.95166026 | XLOC_004515 | UGT2B10 | 0.955725 |
| ENST00000445096 | GAMT | 0.96337582 | ENST00000417121 | UGT2B10 | 0.965021 |
| ENST00000545531 | GAMT | 0.966458451 | ENST00000417851 | UGT2B10 | 0.965981 |
| RefSeq_532_3763 | GAMT | 0.972998962 | ENST00000439524 | UGT2B10 | 0.960268 |
| RefSeq_1589_2127 | GAMT | 0.951173119 | ENST00000445096 | UGT2B10 | 0.96606 |
| RefSeq_2351_1430 | GAMT | 0.959685456 | ENST00000545531 | UGT2B10 | 0.963259 |
| UCSC_6534_1640 | GAMT | 0.963832018 | NRED_1255_416 | UGT2B10 | 0.958617 |
| CombinedLit_370_438 | GAMT | 0.965182636 | ENCODE_1597_545 | UGT2B10 | 0.964971 |
| ENST00000450346 | GBA3 | 0.962919024 | ENCODE_2635_348 | UGT2B10 | 0.969559 |
| RefSeq_260_4911 | GBA3 | 0.954169896 | RefSeq_1589_2127 | UGT2B10 | 0.954605 |
| XLOC_001387 | GBP7 | 0.955423228 | UCSC_5722_1825 | UGT2B10 | 0.957303 |
| ENST00000505626 | GBP7 | 0.966698799 | UCSC_6534_1640 | UGT2B10 | 0.964817 |
| ENST00000507582 | GBP7 | 0.964632997 | UCSC_10912_543 | UGT2B10 | 0.951121 |
| RefSeq_843_3032 | GBP7 | 0.974694765 | ENST00000505626 | UGT2B11 | 0.951704 |
| RefSeq_869_3001 | GBP7 | 0.966011152 | XLOC_012693 | UGT2B4 | 0.958349 |
| UCSC_3328_2475 | GBP7 | 0.951673991 | ENST00000547552 | UGT2B4 | 0.951697 |
| UCSC_4189_2198 | GBP7 | 0.95443355 | lncRNAdb_60_500 | UGT2B4 | 0.952041 |
| UCSC_10912_543 | GBP7 | 0.952234942 | RefSeq_532_3763 | UGT2B4 | 0.952925 |
| UCSC_11415_368 | GCAT | 0.950619645 | RefSeq_843_3032 | UGT2B4 | 0.958986 |
| XLOC_012693 | GCGR | 0.987823182 | RefSeq_869_3001 | UGT2B4 | 0.95531 |
| XLOC_003572 | GCGR | 0.950759117 | RefSeq_1769_1968 | UGT2B4 | 0.965017 |
| ENST00000544018 | GCGR | 0.950033259 | UCSC_3328_2475 | UGT2B4 | 0.959937 |
| ENST00000417121 | GCGR | 0.981939805 | UCSC_8524_1151 | UGT2B4 | 0.955352 |
| ENST00000417851 | GCGR | 0.972762366 | UCSC_11594_306 | UGT2B4 | 0.966374 |
| ENST00000439524 | GCGR | 0.985393158 | asoverlaps_682_1255 | UGT2B4 | 0.955657 |
| ENST00000414604 | GCGR | 0.984563488 | RefSeq_843_3032 | UGT2B7 | 0.958832 |
| ENST00000445096 | GCGR | 0.987159976 | UCSC_5210_1945 | UGT2B7 | 0.951419 |
| ENST00000545531 | GCGR | 0.979894736 | asoverlaps_682_1255 | UGT2B7 | 0.952198 |
| lncRNAdb_60_500 | GCGR | 0.951380902 | XLOC_012693 | UGT3A1 | 0.961965 |
| RefSeq_532_3763 | GCGR | 0.977952061 | XLOC_007658 | UGT3A1 | 0.951192 |
| RefSeq_2351_1430 | GCGR | 0.97138367 | ENST00000417121 | UGT3A1 | 0.961713 |
| RefSeq_3051_754 | GCGR | 0.950259802 | ENST00000417851 | UGT3A1 | 0.962789 |
| UCSC_5722_1825 | GCGR | 0.950799959 | ENST00000439524 | UGT3A1 | 0.963612 |
| UCSC_6534_1640 | GCGR | 0.961930851 | ENST00000414604 | UGT3A1 | 0.952115 |
| UCSC_11594_306 | GCGR | 0.954119346 | ENST00000445096 | UGT3A1 | 0.960725 |
| CombinedLit_370_438 | GCGR | 0.991047006 | ENST00000545531 | UGT3A1 | 0.954232 |
| XLOC_001387 | GCH1 | 0.953822721 | lncRNAdb_60_500 | UGT3A1 | 0.965148 |
| UCSC_5722_1825 | GCH1 | 0.950582057 | NRED_1255_416 | UGT3A1 | 0.964991 |
| XLOC_012693 | GCKR | 0.968055491 | ENCODE_2110_447 | UGT3A1 | 0.958207 |
| ENST00000417121 | GCKR | 0.970282353 | RefSeq_1589_2127 | UGT3A1 | 0.966436 |
| ENST00000417851 | GCKR | 0.960104378 | RefSeq_2351_1430 | UGT3A1 | 0.957454 |
| ENST00000439524 | GCKR | 0.969141142 | UCSC_5210_1945 | UGT3A1 | 0.951876 |
| ENST00000414604 | GCKR | 0.962479964 | UCSC_5722_1825 | UGT3A1 | 0.967569 |
| ENST00000445096 | GCKR | 0.968276906 | UCSC_6534_1640 | UGT3A1 | 0.959428 |
| ENST00000545531 | GCKR | 0.957608941 | RNAz_5553_200 | UGT3A1 | 0.951386 |
| RefSeq_532_3763 | GCKR | 0.953650046 | XLOC_012693 | UPB1 | 0.982542 |
| RefSeq_2866_939 | GCKR | 0.963998619 | XLOC_003572 | UPB1 | 0.97647 |
| RefSeq_3051_754 | GCKR | 0.955981512 | XLOC_004515 | UPB1 | 0.952654 |
| RefSeq_3175_631 | GCKR | 0.962836111 | ENST00000507582 | UPB1 | 0.957602 |
| UCSC_5722_1825 | GCKR | 0.951000564 | ENST00000544018 | UPB1 | 0.954834 |
| UCSC_6534_1640 | GCKR | 0.954876705 | ENST00000417121 | UPB1 | 0.974213 |
| UCSC_11594_306 | GCKR | 0.951220037 | ENST00000417851 | UPB1 | 0.964469 |
| CombinedLit_370_438 | GCKR | 0.966018889 | ENST00000417851 | UPB1 | 0.956778 |
| XLOC_012693 | GDF2 | 0.954369549 | ENST00000439524 | UPB1 | 0.974959 |
| XLOC_004515 | GDF2 | 0.957112202 | ENST00000414604 | UPB1 | 0.967603 |
| ENST00000376608 | GDF2 | 0.953586356 | ENST00000445096 | UPB1 | 0.979836 |
| NRED_1255_416 | GDF2 | 0.961317376 | ENST00000545531 | UPB1 | 0.973698 |
| ENCODE_981_731 | GDF2 | 0.9673542 | ENCODE_1597_545 | UPB1 | 0.96173 |
| ENCODE_2110_447 | GDF2 | 0.951839421 | ENCODE_2110_447 | UPB1 | 0.953732 |
| RefSeq_532_3763 | GDF2 | 0.953008809 | RefSeq_532_3763 | UPB1 | 0.975555 |
| RefSeq_1589_2127 | GDF2 | 0.951136147 | RefSeq_1769_1968 | UPB1 | 0.957736 |
| RefSeq_2351_1430 | GDF2 | 0.956479422 | UCSC_3328_2475 | UPB1 | 0.961664 |
| UCSC_5722_1825 | GDF2 | 0.95989489 | UCSC_6534_1640 | UPB1 | 0.965082 |
| UCSC_8524_1151 | GDF2 | 0.953046698 | UCSC_11594_306 | UPB1 | 0.964441 |
| UCSC_11594_306 | GDF2 | 0.953923448 | CombinedLit_370_438 | UPB1 | 0.975089 |
| ENST00000417121 | GEMC1 | 0.956610131 | asoverlaps_653_1337 | UPB1 | 0.952069 |
| ENST00000417851 | GEMC1 | 0.960728771 | ENST00000417121 | UROC1 | 0.962504 |
| ENST00000545531 | GEMC1 | 0.952818056 | ENST00000417851 | UROC1 | 0.965133 |
| ENCODE_2110_447 | GEMC1 | 0.95968311 | ENST00000545531 | UROC1 | 0.956566 |
| RefSeq_1040_2724 | GEMC1 | 0.961248951 | RefSeq_2351_1430 | UROC1 | 0.964354 |
| RefSeq_2351_1430 | GEMC1 | 0.957196158 | UCSC_6534_1640 | UROC1 | 0.963222 |
| UCSC_10912_543 | GEMC1 | 0.952753157 | XLOC_012847 | VSIG1 | 0.960856 |
| UCSC_6631_1619 | GGH | 0.954451165 | XLOC_012693 | VTN | 0.99334 |
| UCSC_8286_1209 | GGH | 0.950954361 | XLOC_003572 | VTN | 0.950889 |
| XLOC_004515 | GLS2 | 0.955926881 | ENST00000417121 | VTN | 0.98062 |
| ENST00000417121 | GLS2 | 0.9595908 | ENST00000417851 | VTN | 0.971074 |
| ENST00000417851 | GLS2 | 0.954911531 | ENST00000439524 | VTN | 0.979749 |
| ENST00000545531 | GLS2 | 0.956405252 | ENST00000414604 | VTN | 0.973122 |
| UCSC_6534_1640 | GLS2 | 0.959884931 | ENST00000445096 | VTN | 0.985087 |
| ENST00000417121 | GLTPD2 | 0.973502387 | ENST00000545531 | VTN | 0.976868 |
| ENST00000417851 | GLTPD2 | 0.97137393 | lncRNAdb_60_500 | VTN | 0.969663 |
| ENST00000445096 | GLTPD2 | 0.9636662 | RefSeq_532_3763 | VTN | 0.983555 |
| ENST00000545531 | GLTPD2 | 0.975941984 | RefSeq_2351_1430 | VTN | 0.969485 |
| RefSeq_2351_1430 | GLTPD2 | 0.970195372 | UCSC_6534_1640 | VTN | 0.96172 |
| UCSC_6534_1640 | GLTPD2 | 0.96128451 | UCSC_8524_1151 | VTN | 0.963049 |
| XLOC_012693 | GLYAT | 0.980856976 | UCSC_11594_306 | VTN | 0.964431 |
| XLOC_003572 | GLYAT | 0.965778841 | CombinedLit_370_438 | VTN | 0.989602 |
| XLOC_004515 | GLYAT | 0.950890081 | RefSeq_3326_473 | WBSCR26 | 0.977009 |
| ENST00000507582 | GLYAT | 0.959194108 | XLOC_001387 | WNK3 | 0.955925 |
| ENST00000544018 | GLYAT | 0.981199254 | XLOC_002845 | WNK3 | 0.962047 |
| ENST00000417121 | GLYAT | 0.96782129 | XLOC_002845 | WNK3 | 0.960225 |
| ENST00000417851 | GLYAT | 0.960211462 | ENST00000507582 | WNK3 | 0.9677 |
| ENST00000439524 | GLYAT | 0.98151771 | ENCODE_2110_447 | WNK3 | 0.964349 |
| ENST00000414604 | GLYAT | 0.978960354 | UCSC_5722_1825 | WNK3 | 0.965044 |
| ENST00000445096 | GLYAT | 0.971075657 | UCSC_5722_1825 | WNK3 | 0.976888 |
| ENST00000545531 | GLYAT | 0.9719627 | UCSC_11594_306 | WNK3 | 0.952544 |
